# BoolForge: controlled generation and analysis of Boolean functions and networks

**DOI:** 10.1093/bioinformatics/btag576

**Published:** 2026-07-31

**Authors:** Claus Kadelka, Benjamin Coberly

**Affiliations:** Department of Mathematics, Iowa State University, Ames, IA 50011, United States; Department of Mathematics, Iowa State University, Ames, IA 50011, United States; Department of Computer Science, Iowa State University, Ames, IA 50011, United States

## Abstract

**Motivation:**

Boolean networks are a widely used modeling framework in systems biology for studying gene regulation, signal transduction, and cellular decision-making. Empirical studies indicate that biological Boolean networks exhibit a high degree of canalization, a property of Boolean update rules that stabilizes dynamics and constrains state transitions. Despite its central role, existing software packages provide limited support for the systematic generation of Boolean functions and networks with prescribed canalization properties.

**Results:**

We present BoolForge, a Python toolbox for the random generation and analysis of Boolean functions and networks, with a particular focus on canalization. BoolForge enables users to (i) generate random Boolean functions with specified canalizing depth, layer structure, and related constraints; (ii) construct Boolean networks with tunable topological and functional properties; and (iii) analyze structural and dynamical features including canalization measures, robustness, modularity, and attractor structure. By enabling controlled generation alongside analysis, BoolForge facilitates ensemble-based investigations of structure-dynamics relationships, benchmarking of theoretical predictions, and construction of biologically informed null models for Boolean network studies.

**Availability and implementation:**

BoolForge is implemented in Python (≥3.10) and can be installed via pip install boolforge. Source code and documentation are available at https://github.com/ckadelka/BoolForge. A comprehensive tutorial compendium is available as Supplementary Material at *Bioinformatics* online.

## 1 Introduction

Boolean network (BN) models are widely used to study qualitative aspects of regulation, decision-making, and information processing in biological systems (Wang *et al.* 2012, Hemedan *et al.* 2022). From the early work of Kauffman and Thomas on genetic regulatory circuits (Kauffman 1969, Thomas 1973) to recent large-scale and data-driven applications (Zerrouk *et al.* 2024), Boolean networks provide an interpretable and computationally tractable representation of complex dynamical systems.

Empirical studies of biological Boolean network models consistently reveal strong *canalization* in their update rules—a hierarchical and redundancy-rich organization of input variables (Daniels *et al.* 2018, Gates *et al.* 2021, Kadelka *et al.* 2024). Canalization is thought to contribute to the stability, robustness, and evolvability of living systems. Theoretical advances have characterized canalizing depth, layer structure, collective canalization, and input redundancy (He and Macauley 2016, Kadelka *et al.* 2017, Dimitrova *et al.* 2022), providing a detailed mathematical understanding of this functional architecture. These developments motivate the need for computational tools capable of systematically generating and analyzing Boolean functions and networks with prescribed canalization properties.

Most existing tools for Boolean network analysis, including BoolNet (Müssel *et al.* 2010), PyBoolNet (Klarner *et al.* 2017), Cyclone (Dimitrova *et al.* 2023), and biobalm (Trinh *et al.* 2025), and tools from the CoLoMoTo ecosystem (Noël *et al.* 2025), such as ViSiBool (Schwab *et al.* 2017), primarily focus on simulation, attractor identification, and dynamical analysis. BoolNet provides generators for canalizing and nested canalizing Boolean functions, while CANA supports analysis of collective canalization and redundancy in Boolean functions and networks. However, support for function-uniform generation of Boolean functions and networks under precisely specified structural constraints—such as canalizing depth, canalizing layer structure, or bias—remains limited. Yet the ability to generate controlled ensembles of Boolean functions and networks is essential for hypothesis testing, benchmarking, and investigating how structural features such as canalization, bias, and connectivity shape network behavior. Such ensembles provide biologically informed null models against which structural and dynamical properties of curated regulatory networks can be evaluated.

To address this gap, we introduce BoolForge, a Python toolbox for the controlled generation and analysis of Boolean functions and networks, with an emphasis on canalization and ensemble-based investigations. BoolForge provides (i) flexible function-uniform sampling algorithms—in which all Boolean functions satisfying specified constraints are sampled with equal probability—for generating Boolean functions with user-defined structural and functional properties, including canalizing depth, layer structure, bias, and redundancy; (ii) methods for constructing Boolean networks with tunable wiring diagrams and rule distributions; and (iii) tools for analyzing structural, modular, and dynamical features. By integrating controlled generation with analysis in a single framework, BoolForge enables systematic ensemble studies, biologically informed null-model construction, and rapid prototyping of Boolean network models. In the following sections, we describe the architecture and core functionality of BoolForge and illustrate its capabilities through representative ensemble-based applications.

## 2 Architecture and core functionality

**Figure 1:**
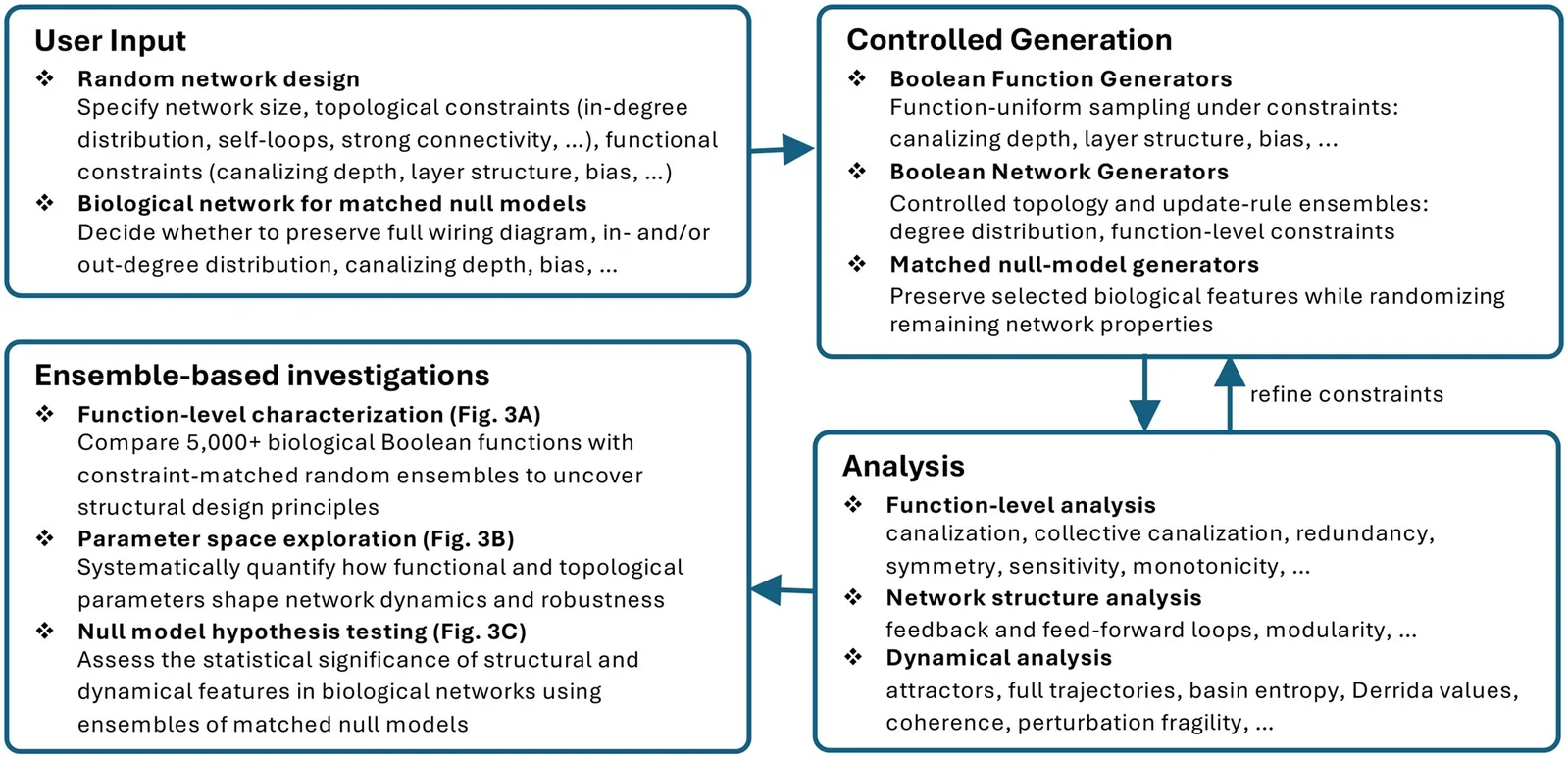
Overview of the BoolForge framework. Starting from either a random network design or a biological reference network, user-defined constraints guide the controlled, function-uniform generation of Boolean functions, networks, and matched null models. Integrated analysis tools quantify structural and dynamical properties at both the function and network level. The generation–analysis cycle supports iterative refinement of constraints and enables ensemble-based investigations including functional characterization, parameter space exploration, and null-model hypothesis testing (examples in Fig. 3).

BoolForge is designed to support systematic, ensemble-based investigation of Boolean functions and networks. Its architecture separates rule-level structure from network topology and integrates controlled random generation with structural and dynamical analysis. The framework centers on two primary abstractions, BooleanFunction and BooleanNetwork, enabling independent manipulation of update rules and wiring diagrams while maintaining interoperability with existing software (Fig. 1).

### 2.1 Design and architecture

BoolForge is organized around three guiding design principles. First, rule structure and network topology are treated as conceptually distinct objects. A BooleanFunction represents a local update rule independently of its network context, while a BooleanNetwork combines such functions with an explicit wiring diagram. For example, a Boolean function can be instantiated directly from a logical expression:

from boolforge import BooleanFunction

f = BooleanFunction(“x1 & !x2 | x3”)

Boolean networks are then constructed by combining such functions with a wiring diagram, which can either be explicitly specified or imported from a networkx. DiGraph object. This separation enables controlled experiments in which functional and topological constraints can be varied independently.

Second, random generation and analysis are integrated within a single framework. Sampling algorithms are implemented alongside structural and dynamical metrics, allowing users to generate constrained ensembles and immediately evaluate their properties without switching software environments.

Third, the package emphasizes extensibility and interoperability. Models can be constructed from truth tables or logical expressions, converted from external formats, and exported for downstream analysis in complementary tools.

### 2.2 Controlled generation and analysis of Boolean functions

While generators for some basic function classes are available in related software packages such as CANA (Marcus *et al.* 2025) and PyBoolNet (Klarner *et al.* 2017), BoolForge provides function-uniform generators for several structurally constrained function families, including non-degenerate Boolean functions, linear and affine functions (Chandrasekhar *et al.* 2023), functions with specified canalizing depth (He and Macauley 2016), nested canalizing functions (Li *et al.* 2013), functions with prescribed canalizing layer structure (Dimitrova *et al.* 2022), and functions with fixed Hamming weight or prescribed bias (Shmulevich and Kauffman 2004). Here, “function-uniform” means that every Boolean function within the specified constraint class is sampled with equal probability, rather than uniformly over parameterizations, which can substantially bias the resulting ensemble (Ghosh and Kadelka 2026). For example, random_function(n = 5, depth = 2, exact_depth = True) generates a random 5-input Boolean function with exact canalizing depth 2, while random_function(n = 4, layer_structure = [1,3]) generates a random nested canalizing function with canalizing layer structure (1, 3). These generators enable controlled sampling within precisely defined structural classes, facilitating hypothesis testing about the role of canalization, bias, or redundancy in network behavior.

In addition to generation, BoolForge provides comprehensive structural analysis of Boolean functions. It identifies essential and non-essential variables, monotonicity properties, and input symmetries, and computes measures such as average sensitivity. Canalization is quantified through multiple complementary frameworks, including extended monomial representations revealing canalizing layer structure (He and Macauley 2016, Dimitrova *et al.* 2022), canalizing strength (Kadelka *et al.* 2023a), and input redundancy and effective degree (Gates *et al.* 2021, Marcus *et al.* 2025). Together, these tools provide a detailed characterization of the functional building blocks underlying Boolean networks.

### 2.3 Controlled generation of Boolean networks and null models

A BooleanNetwork consists of a collection of BooleanFunction objects together with a wiring diagram specifying regulatory interactions. Networks can be constructed manually, imported from standard text-based formats such as .bnet, converted from CANA (Marcus *et al.* 2025) network objects, or generated randomly.

Random network generation proceeds in two steps. First, a wiring diagram is constructed. Users may fix in-degrees, sample from specified in-degree distributions, enforce strong connectivity, allow or disallow self-loops, or provide custom wiring diagrams (e.g. from existing biological Boolean network models). Second, update rules are assigned to nodes using any of the constrained function generators described above. This two-step construction separates topological and functional constraints, allowing their independent manipulation and systematic study.

BoolForge also provides routines for generating biologically informed null models to support statistical hypothesis testing. These null ensembles preserve selected features of a reference network—such as degree distribution, canalizing depth, layer structure, or bias—while randomizing other aspects. For example, random_null_model (bn, wiring_diagram=’fixed’, preserve_canalizing_depth = True) generates randomized networks that preserve both the wiring diagram of the reference network bn and the canalizing depth of each update rule, while randomizing other functional details. Such matched ensembles enable rigorous comparison of observed structural or dynamical properties against expectations under controlled constraints.

### 2.4 Structural and dynamical analysis of Boolean networks

Once networks are constructed, BoolForge provides integrated tools for structural and dynamical analysis. Structural routines identify canonical motifs, including feedback and feed-forward loops (Alon 2007, Kadelka *et al.* 2024), and support quantitative assessment of modular organization (Kadelka *et al.* 2023b).

For dynamical analysis, BoolForge provides numba-accelerated exact and approximate methods for analyzing dynamics under synchronous and asynchronous update schemes, as well as a variety of robustness measures, including Derrida values, perturbation fragility, and network, basin, and attractor coherence measures (Derrida and Pomeau 1986, Willadsen *et al.* 2008, Park *et al.* 2023, Bavisetty *et al.* 2026). For example, a random Boolean network can be generated and analyzed using the following commands:

from boolforge import random_network

bn = random_network(N = 20, n = 3)

info = bn.get_attractors_and_robustness_synchronous_exact()

These tools enable investigation of how canalization, bias, and topology influence long-term dynamics and robustness properties. Random networks generated with BoolForge can further be exported in standard Boolean-network formats, such as.bnet, enabling seamless interoperability with tools such as biobalm (Trinh *et al.* 2025), Cubewalkers (Park *et al.* 2023), and pystablemotifs (Rozum *et al.* 2022). By combining controlled generation with systematic analysis, BoolForge facilitates ensemble-based exploration of structure–dynamics relationships in Boolean networks.

### 2.5 Computational considerations

Computational complexity in Boolean network analysis arises from two primary sources. First, Boolean functions with *n* inputs are represented by truth tables of size $2^{n}$, implying exponential scaling in the in-degree. In practice, however, this exponential dependence on in-degree is typically not restrictive because regulatory functions in curated biological Boolean network models typically have small in-degrees (often n≤4) (Kadelka *et al.* 2024). Second, a Boolean network with *N* nodes has a state space of size $2^{N}$, which fundamentally limits exhaustive dynamical analysis to moderate network sizes.

**Figure 2:**
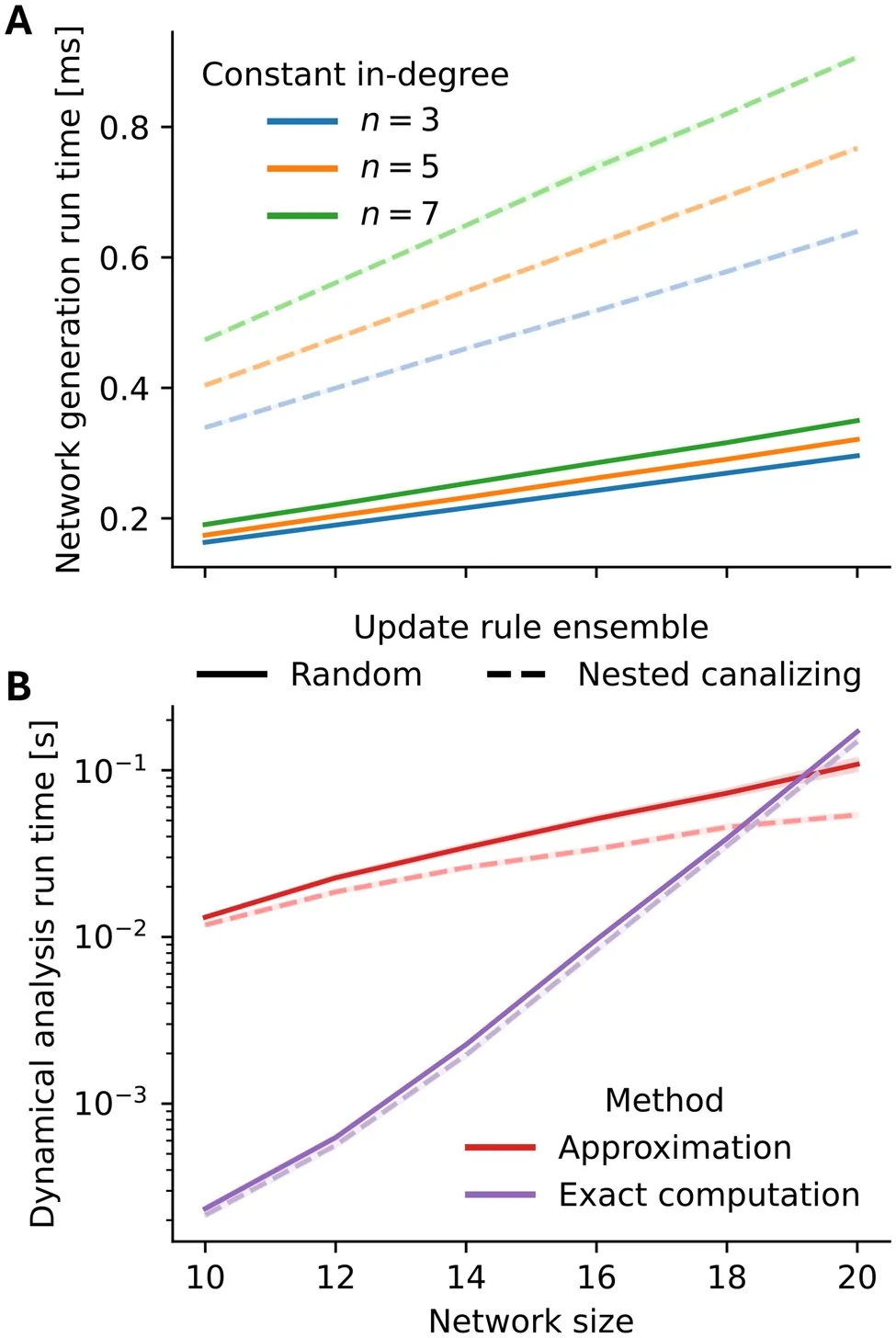
Runtime benchmarks for representative generation and analysis tasks in BoolForge. (A) Average runtime for generating random Boolean networks with constant in-degree n∈{3,5,7}, governed by random or nested canalizing update rules. (B) Average runtime for dynamical analysis of synchronously updated Boolean networks with constant in-degree n=3, using either exact or trajectory-based approximate computation of attractors and robustness. Approximate computations used the default BoolForge setting of 500 random initial conditions. In both panels, lines show averages over 100 independent replicates per network size and shaded regions indicate 95% confidence intervals. Exact dynamical analysis exhibits exponential scaling with network size due to state-space enumeration, whereas approximate methods remain computationally tractable for larger networks.

To accommodate different computational regimes, BoolForge provides both exact algorithms and trajectory-based approximation methods for dynamical analysis. Structural analysis and controlled network generation scale efficiently to large ensembles of networks, whereas exhaustive attractor and robustness computations become increasingly expensive with growing network size. Trajectory-based approximation methods estimate dynamical quantities such as attractor statistics and robustness measures from randomly sampled initial conditions by iteratively updating trajectories until attractors are reached, thereby avoiding exhaustive state-space enumeration. Figure 2 illustrates the computational scaling of representative generation and analysis tasks implemented in BoolForge. In practice, exact dynamical analysis is typically feasible for networks with up to approximately N≈25 nodes, while approximation methods enable scalable analysis for larger systems.

## 3 Applications

**Figure 3:**
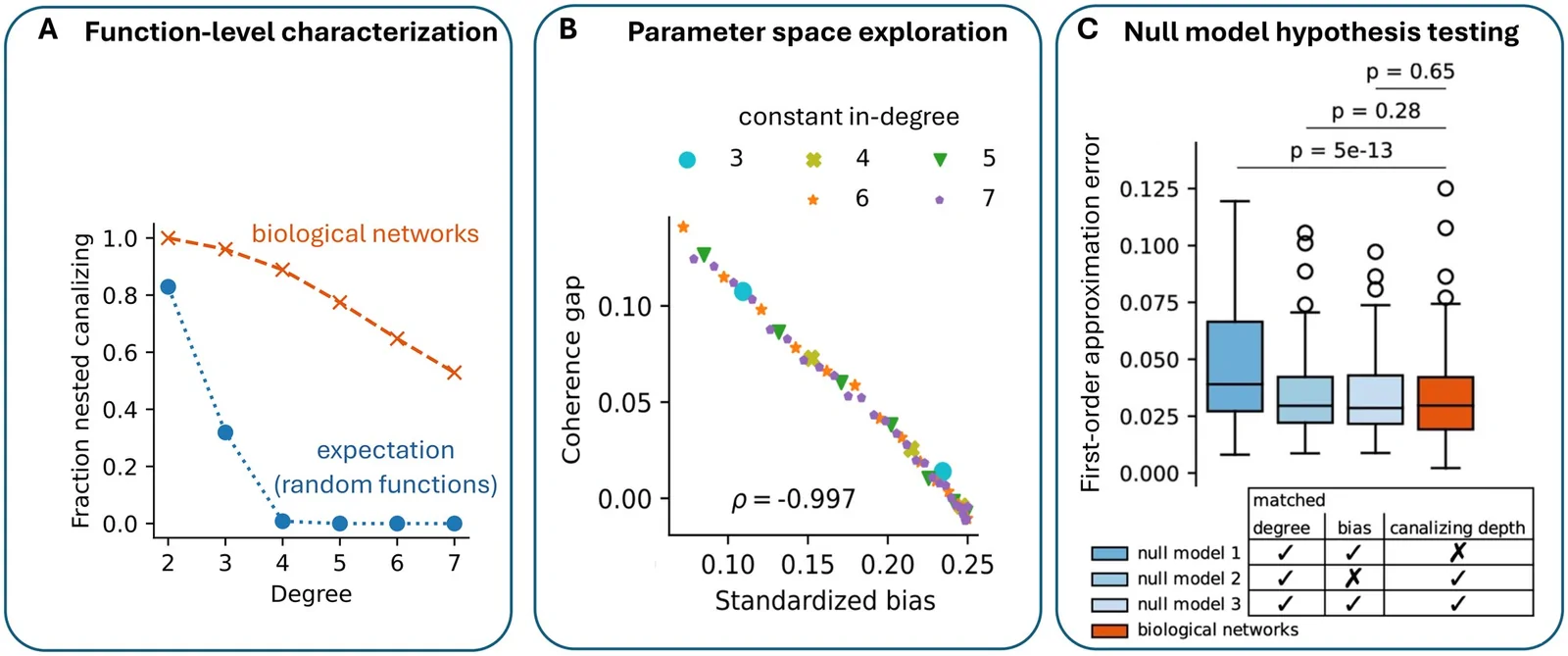
Representative ensemble analyses enabled by BoolForge. (A) Proportion of nested canalizing functions among 5112 Boolean functions from 122 curated biological models compared with constraint-matched random expectations (Kadelka *et al*. 2024). (B) Coherence gap (difference between basin and attractor coherence) as a function of standardized bias across ensembles of 10 000 randomly generated nested canalizing networks with fixed layer structure and in-degree (Bavisetty *et al*. 2026). (C) First-order approximation error in 122 biological networks compared with three types of matched null models generated using BoolForge (100 realizations per network and null model type) (Kadelka and Murrugarra 2024).

To illustrate the capabilities of BoolForge, we present representative ensemble-based analyses enabled by the framework (Fig. 3). The analyses in panels A and B can be reproduced using tutorial notebooks distributed with the package, while panel C illustrates how BoolForge can generate matched null ensembles for evaluating dynamical approximations (Kadelka and Murrugarra 2024). These examples highlight how controlled random generation and biologically informed null models support the investigation of regulatory design principles in curated Boolean network models.

### 3.1 Functional enrichment in biological update rules

Empirical studies of curated biological Boolean network models reveal strong enrichment for nested canalizing functions and related hierarchical structures (Kadelka *et al.* 2024). Using BoolForge, one can uniformly sample constraint-matched random functions with identical in-degree or bias and compare the observed prevalence of nested canalizing functions to null expectations (Fig. 3A). Such analyses require function-uniform sampling within restricted function classes (Ghosh and Kadelka 2026), including classes with prescribed canalizing depth or canalizing layer structure, capabilities largely absent from existing dynamical-analysis-focused tools. The resulting comparisons indicate that biological update rules are significantly more canalizing than expected under matched random ensembles.

### 3.2 Disentangling bias and canalization in network stability

Canalization is known to influence network stability and robustness, but its dynamical effects are often mediated through bias or effective connectivity. By generating large ensembles of networks with fixed in-degree and specified canalizing layer structures, BoolForge enables systematic exploration of these relationships. For example, ensemble experiments show that, in synchronously updated Boolean networks, the difference between basin coherence and attractor coherence (the coherence gap) is strongly associated with the standardized bias (Fig. 3B) (Bavisetty *et al.* 2026). This example illustrates how controlled ensembles can disentangle correlated structural properties of Boolean network models.

### 3.3 Evaluating approximation frameworks with matched null models

Matched null models are also useful for evaluating theoretical approximations of Boolean network dynamics. Using BoolForge, null networks can be generated that preserve selected structural features of a reference network, such as degree distribution or canalization properties. In studies of first-order dynamical approximations, such matched ensembles revealed systematic differences in approximation error that diminish when canalization is incorporated (Fig. 3C) (Kadelka and Murrugarra 2024). This example illustrates how biologically informed null models can identify which structural features are necessary to reproduce observed dynamical behavior.

Together, these examples illustrate how BoolForge enables systematic and reproducible ensemble analyses of Boolean network models. By integrating controlled random generation with structural and dynamical analysis, the framework supports hypothesis-driven exploration of structure–dynamics relationships beyond the capabilities of traditional simulation-centered tools. These workflows generalize naturally to any curated Boolean network model, enabling functional characterization of empirical update rules, parameter space exploration across structurally defined ensembles, and rigorous null-model-based hypothesis testing.

## Supplementary Material

btag576_Supplementary_Data

## Data Availability

BoolForge is implemented in Python (≥3.10), is platform independent, and is distributed under the MIT License. The package can be installed via pip install boolforge. Source code, documentation, and tutorial notebooks are available at https://github.com/ckadelka/BoolForge and archived at https://doi.org/10.5281/zenodo.20657329. A comprehensive tutorial compendium is also provided as Supplementary Material, available as supplementary data at *Bioinformatics* online. The authors commit to maintaining the repository for at least two years following publication.
