# Supplementary material for: BoolForge: controlled generation and analysis of Boolean functions and networks
Source: Bioinformatics. 2026 Jul 31;42(8):btag576. doi: 10.1093/bioinformatics/btag576 (PMC13456963; doi:10.1093/bioinformatics/btag576)
Supplement: Supplementary file 1 [file btag576_supplementary_data.pdf]

Boolean Network Modeling in Systems Biology: A Hands-On Tutorial with BoolForge

Claus Kadelka

June 10, 2026

Contents

Abstract 5

Preface 6

Structure of the tutorials 7

Introduction 8

Positioning within the literature 8

1 Working with Boolean Functions 10

1.1 What you will learn 10
1.2 Setup 10
1.3 Create a Boolean function 10
1.3.1 Create Boolean functions from a truth table 10
1.3.2 Create Boolean functions from text 11
1.3.3 Combining BooleanFunction objects 11
1.4 Attributes of BooleanFunction 12
1.5 Basic properties of Boolean functions 13
1.6 Logical and polynomial representations 14
1.7 Summary 15
1.7.1 Boolean functions 15
1.7.2 Representations of Boolean functions 15
1.7.3 Variable names and ordering 15
1.7.4 Basic properties of Boolean functions 15
1.7.5 Conversions and interoperability 15
1.8 Frequently Asked Questions 15
1.8.1 Why does the order of variables matter? 15
1.8.2 How do I choose between defining a function via a truth table or via an expression? 16
1.8.3 What is the difference between `get_type_of_inputs()` and monotonicity? 16
1.8.4 Quick Reference 16

2 Advanced Concepts for Boolean Functions 17

2.1 What you will learn 17
2.2 Setup 17
2.3 Symmetries in Boolean functions 17
2.4 Degenerate functions 18
2.5 Activities and sensitivities 19
2.5.1 Activity 19
2.5.2 Average sensitivity 19
2.5.3 Exact vs Monte Carlo computation 19
2.5.4 Computing activities and sensitivities 20
2.6 Summary 21

3 Canalization 22

3.1 What you will learn 22
3.2 Setup 22
3.3 Canalizing variables and layers 22
3.3.1 Canalizing depth and nested canalization 23
3.3.2 Canalizing layer structure 24
3.4 Collective canalization 24
3.4.1 Canalizing strength 25
3.4.2 Distribution of canalizing strength 26
3.5 Canalization as a measure of input redundancy 27
3.6 Summary 28

4 Random Boolean function generation **29**
4.1 What you will learn . . . 29
4.2 Setup . . . 29
4.3 Generating random Boolean functions . . . 29
4.4 Parity functions . . . 30
4.5 Functions with prescribed canalizing properties . . . 30
4.5.1 Functions with prescribed canalizing layer structure . . . 30
4.5.2 Functions with prescribed canalizing depth . . . 31
4.6 Allowing degenerate functions . . . 32
4.7 Functions with prescribed Hamming weight . . . 34
4.8 Biased and absolutely biased functions . . . 35
4.9 Summary . . . 37
4.10 Common pitfalls . . . 37

5 Ensemble experiments with random Boolean functions **39**
5.1 What you will learn . . . 39
5.2 Setup . . . 39
5.3 Prevalence of canalization . . . 39
5.3.1 Restricting to canalizing functions . . . 41
5.4 Collective canalization vs degree . . . 42
5.4.1 Stratification by canalizing depth . . . 44
5.4.2 Correlation between canalizing strength and input redundancy . . . 46
5.5 Correlation between canalization and bias . . . 47
5.5.1 Degeneracy vs bias . . . 49
5.6 Analyzing functions with specific canalizing layer structure . . . 50
5.7 Summary . . . 52

6 Working with Boolean Networks **53**
6.1 What you will learn . . . 53
6.2 Setup . . . 53
6.3 Boolean network theory . . . 53
6.4 Wiring diagrams . . . 53
6.4.1 Example with constants and unequal degrees . . . 54
6.5 Creating Boolean networks . . . 56
6.5.1 Creating networks from strings . . . 57
6.5.2 Interoperability with CANA . . . 58
6.6 Types of nodes in Boolean networks . . . 58
6.7 Boolean network properties . . . 60
6.8 Manipulation and control of Boolean networks . . . 62
6.8.1 Node controls . . . 63
6.8.2 Edge controls . . . 64
6.9 Outlook . . . 65

7 Dynamics of Boolean Networks **66**
7.1 What you will learn . . . 66
7.2 Setup . . . 66
7.3 State space of a Boolean network . . . 66
7.4 Dynamics of synchronous Boolean networks . . . 67
7.4.1 Exact computation . . . 67
7.4.2 Exhaustive attractor computation . . . 68
7.4.3 Monte Carlo simulation . . . 69
7.5 Dynamics of asynchronous Boolean networks . . . 70
7.5.1 Attractors under general asynchronous update . . . 71
7.5.2 Monte Carlo approximation . . . 72

7.5.3 Sampling from a fixed initial condition . . . 72
7.6 Summary . . . 73

8 Perturbation and sensitivity analysis of Boolean networks 74
8.1 What you will learn . . . 74
8.2 Setup . . . 74
8.3 Exact attractors and robustness measures . . . 74
8.4 Network-, basin- and attractor-level robustness in synchronous networks . . . 75
8.5 Network-, basin- and attractor-level robustness in asynchronous networks . . . 76
8.6 Approximate robustness for larger synchronous networks . . . 77
8.7 Derrida value: dynamical sensitivity . . . 77
8.8 Summary . . . 77

9 Random Boolean network generation 78
9.1 What you will learn . . . 78
9.2 Setup . . . 78
9.3 Generating random wiring diagrams . . . 78
9.3.1 Allowing self-regulation . . . 79
9.3.2 Poisson in-degree distributions . . . 80
9.3.3 Avoiding output nodes . . . 81
9.3.4 Strong connectedness . . . 81
9.3.5 Fixed wiring diagrams . . . 81
9.4 Specifying functional constraints . . . 82
9.4.1 Parity functions . . . 82
9.4.2 Canalizing functions . . . 82
9.4.3 Biased functions . . . 83
9.5 Summary . . . 84

10 Ensemble experiments with random Boolean networks 85
10.1 What you will learn . . . 85
10.2 Setup . . . 85
10.3 NK Kauffman networks . . . 85
10.4 BoolForge philosophy: regulatory functions are non-degenerate . . . 86
10.5 Random networks with prescribed canalization . . . 88
10.6 Summary and outlook . . . 89

11 Curated biological Boolean networks and null models 90
11.1 What you will learn . . . 90
11.2 Setup . . . 90
11.3 Loading model repositories . . . 90
11.4 Analyzing model repositories . . . 91
11.5 Null models . . . 91
11.5.1 Randomization of the wiring diagram . . . 92
11.5.2 Randomization of the update functions . . . 93
11.6 Example use case: high coherence of biological networks . . . 94
11.7 Summary . . . 96

References 98

Abstract

Boolean networks are a widely used modeling framework for studying complex dynamical systems in systems biology, including gene regulatory and signaling networks. Despite their conceptual simplicity, practical challenges – such as constructing biologically meaningful models, analyzing dynamical behavior, and generating appropriate null models – often limit their accessibility, reproducibility, and systematic use.

We present a comprehensive, hands-on tutorial to Boolean network modeling and analysis, accompanied by the Python package BoolForge. This tutorial is designed for researchers and students with diverse backgrounds and provides a step-by-step introduction ranging from basic concepts to advanced topics. Covered topics include Boolean functions and their representations, canalization, random function generation under structural constraints, construction of Boolean networks and wiring diagrams, synchronous and asynchronous dynamics, attractor and basin analysis, robustness and coherence measures, and the generation of controlled null models for hypothesis testing.

Emphasis is placed on reproducible computational workflows and the systematic investigation of structure–dynamics relationships. Through a series of progressively developed examples and executable code snippets, readers learn how to construct, analyze, and interpret Boolean network models, as well as how to generate ensembles of randomized networks that preserve key structural features such as degree distribution, bias, and canalizing depth. Code and tutorials are openly available at https://github.com/ckadelka/BoolForge.

This tutorial serves both as a practical introduction for new users and as a reference for advanced researchers seeking to apply Boolean network methods in systems biology. By integrating theory, algorithms, and software implementation, it aims to facilitate the adoption of rigorous and reproducible modeling practices in the study of complex biological systems.

Preface

This tutorial is designed as a hands-on, computational introduction to Boolean network modeling using BoolForge.

Rather than focusing purely on theory, we emphasize executable workflows and reproducible experiments. The goal is to enable readers to explore structure-dynamics relationships directly through code.

To illustrate the type of analyses enabled by BoolForge, we begin with a simple example reproducing Derrida's classical phase transition (see Derrida and Pomeau [1986]) in a few lines of BoolForge code.

```
import boolforge as bf
import matplotlib.pyplot as plt

N = 100          # network size
ks = range(1,5)  # constant in-degree
n_networks = 50  # ensemble size
p = 0.5          # bias p: probability of ones in truth table

derrida_values = []
for k in ks:
    derrida_values.append([])
    for _ in range(n_networks):
        bn = bf.random_network(N, k, bias = p, allow_degenerate_functions=True)
        derrida_values[-1].append( bn.get_derrida_value(exact=True) )

plt.boxplot(derrida_values, positions=list(ks))
plt.axhline(1, linestyle="--", color="gray", label="critical value")
plt.plot(ks, [2*k*p*(1-p) for k in ks], "o-", label=r"$2kp(1-p)$ (annealed theory)")
plt.xlabel("Constant in-degree k")
plt.ylabel("Derrida value")
plt.legend(frameon=False);
```

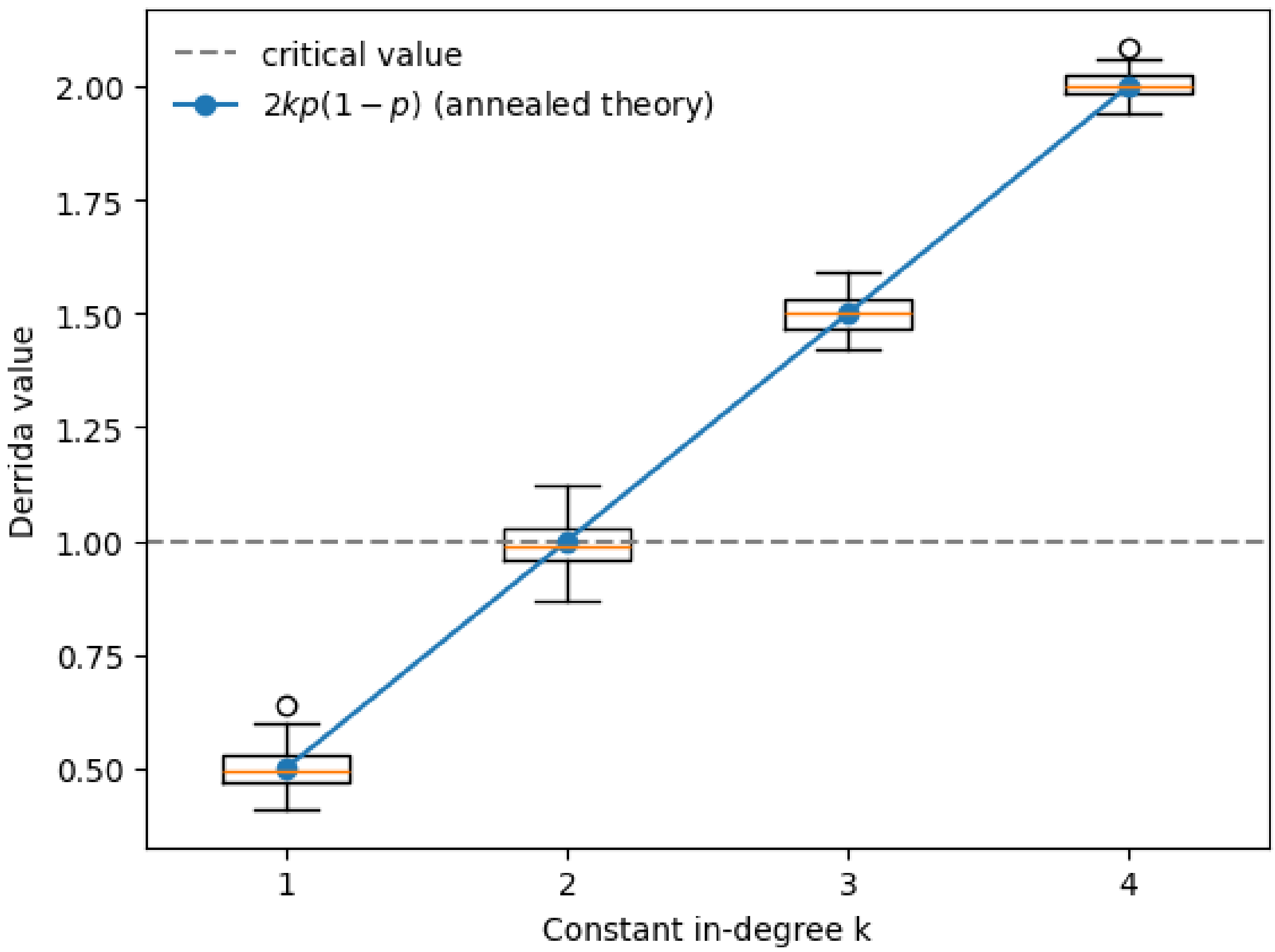

The Derrida value measures the average number of nodes affected by a single-bit random perturbation after one synchronous update of the network.

Structure of the tutorials

The tutorials gradually introduce the main concepts and tools provided by BoolForge, moving from individual Boolean functions to full Boolean network models and their dynamical analysis.

- *Boolean functions:* representation and structural analysis
- *Canalization:* redundancy and robustness of regulatory rules
- *Random function generation:* sampling functions with prescribed properties
- *Boolean networks:* construction and wiring diagrams
- *Network dynamics:* attractors and state transition graphs
- *Stability and robustness:* sensitivity to perturbations
- *Random network ensembles:* statistical analysis of network dynamics
- *Biological models:* analysis of curated regulatory networks

Each tutorial contains executable code examples illustrating how these ideas can be explored using BoolForge. Corresponding Jupyter notebook (ipynb) files can be found at https://github.com/ckadelka/BoolForge/tree/main/tutorials. Readers are encouraged to run the code cells and modify the examples to explore their own Boolean functions and networks.

Introduction

Boolean networks have emerged as a central modeling framework for studying complex dynamical systems in systems biology, including gene regulatory, signaling, and cellular decision-making networks [Kauffman, 1969]. In a Boolean network, each component is represented by a binary variable, and its dynamics are governed by logical update rules that capture regulatory interactions. Despite their conceptual simplicity, Boolean networks are capable of reproducing rich dynamical behavior such as multistability, oscillations, and robustness to perturbations, making them a widely used tool for qualitative modeling when detailed kinetic information is unavailable.

Over the past decades, Boolean network models have been successfully applied to a broad range of biological systems, from cell cycle regulation to developmental processes and disease-related signaling pathways. At the same time, theoretical advances have deepened our understanding of how structural properties—such as network topology, regulatory logic, and canalization—shape dynamical behavior. In particular, concepts such as attractors, basins of attraction, robustness, and sensitivity have become standard tools for analyzing the long-term behavior of these systems.

However, despite this progress, practical challenges remain. Constructing biologically meaningful Boolean network models, analyzing their dynamics, and interpreting results in a reproducible and systematic way can be difficult, especially for researchers new to the field. Moreover, many commonly used approaches rely on ad hoc randomization or lack appropriate null models, making it challenging to disentangle the effects of network structure from those of the update rules. As a result, there is a growing need for tools and tutorials that integrate theoretical concepts with practical, reproducible workflows.

This tutorial addresses these challenges by providing a comprehensive, hands-on introduction to Boolean network modeling and analysis, accompanied by the Python package BoolForge. The goal is to bridge the gap between theory and practice by guiding the reader from fundamental concepts to advanced applications, with an emphasis on reproducibility, clarity, and methodological rigor.

We begin by introducing Boolean functions and their representations, including truth tables and logical expressions, and discuss key structural properties such as bias, essential variables, and canalization. We then present methods for generating random Boolean functions under structural constraints, including k-canalizing functions and nested canalizing functions, which play a central role in biological modeling. Building on these foundations, we introduce Boolean networks and wiring diagrams, and demonstrate how to construct and manipulate models programmatically.

A central focus of this tutorial is the analysis of network dynamics. We cover both synchronous and asynchronous update schemes, and describe methods for identifying attractors, computing basins of attraction, and quantifying dynamical properties such as robustness, fragility, and coherence. In addition, we emphasize the importance of ensemble-based approaches and controlled null models, which allow systematic investigation of how structural features—such as degree distribution, bias, and canalizing depth—affect network behavior.

Throughout the tutorial, we provide executable code examples and reproducible workflows that enable readers to directly apply the presented methods to their own research questions. By integrating theory, algorithms, and software implementation, this tutorial aims to make Boolean network modeling more accessible, transparent, and rigorous.

This document is intended for a broad audience, including students, experimentalists, and computational researchers. Readers new to Boolean networks will find a step-by-step introduction to the key concepts and methods, while more experienced users may use this tutorial as a reference for advanced topics such as constrained random function generation and null model construction.

Positioning within the literature

While numerous introductions to Boolean networks exist, they are often fragmented across textbooks, review articles, and software-specific documentation, and typically emphasize either theoretical aspects or particular applications. In contrast, this tutorial provides a unified, end-to-end treatment that integrates structural theory, dynamical analysis, and reproducible computational workflows within a single framework. A distinguishing

feature is the systematic treatment of constrained random function and network generation, including canalization-controlled ensembles and null models that preserve key structural properties. This enables rigorous investigation of structure–dynamics relationships beyond traditional random network paradigms. By combining these methodological advances with practical implementation in BoolForge, this work is positioned not merely as an introduction, but as a comprehensive reference for modern Boolean network analysis in systems biology.

Citation note. If you use BoolForge in your research, please cite the accompanying software paper [Kadelka and Coberly, 2025]. Further, if this tutorial contributed to your modeling, analysis, or interpretation, please cite our work.

1 Working with Boolean Functions

Boolean functions are the building blocks of Boolean network models used to represent gene regulatory networks, signaling pathways, and other biological control systems. Understanding how to create and analyze individual Boolean functions is essential before studying network-level dynamics.

In this tutorial, we explore the `BooleanFunction` class — the foundation of BoolForge. Boolean functions form the regulatory rules in Boolean network models of gene regulation, so understanding their structure is essential before studying networks.

1.1 What you will learn

In this tutorial you will:

- create Boolean functions from truth tables and from textual expressions,
- inspect core attributes such as degree, variable names, and stored properties,
- compute basic structural properties (essential variables, Hamming weight, bias),
- convert Boolean functions into logical and polynomial representations,
- and interface with CANA objects.

1.2 Setup

```
import boolforge as bf
```

1.3 Create a Boolean function

Boolean functions can be described in logical form, as polynomials, or as truth tables. BoolForge treats Boolean functions as binary vectors of length 2^n, where n is the number of inputs. The vectors describe the *right side* of the truth table. The left side of the truth table is not stored because it is the same for any function with n inputs. For example, the function

$$f(A, B) = A \wedge B$$

is stored as `[0, 0, 0, 1]`, corresponding to:

A	B	f(A,B)
0	0	0
0	1	0
1	0	0
1	1	1

1.3.1 Create Boolean functions from a truth table

A `BooleanFunction` object can be generated by specifying the right side of the truth table, i.e., by providing a binary vector of length 2^n for any $n \geq 0$. For example, to create the AND function above, we can write

```
f = bf.BooleanFunction([0, 0, 0, 1], name="f_AND") #name is optional
print("f:", f)
print("Truth table of f:\n", f.to_truth_table().to_string())
```

```
f: [0 0 0 1]
Truth table of f:
    x0  x1  f_AND
0   0   0      0
1   0   1      0
```

```
2   1   0       0
3   1   1       1
```

Any Boolean function is stored as right side of the truth table. That is, the outputs are ordered by the binary representation of inputs:

- Position 0 –> (A,B) = (0,0)
- Position 1 –> (A,B) = (0,1)
- Position 2 –> (A,B) = (1,0)
- Position 3 –> (A,B) = (1,1)

1.3.2 Create Boolean functions from text

Boolean functions can also be created from textual expressions. For example, to define the same function as f, we can write

```
f2 = bf.BooleanFunction("A and B")
print("f2:", f2)
```

```
f2: [0 0 0 1]
```

The text processor is fairly versatile. For example, we can define the same function as f also by writing

```
f3 = bf.BooleanFunction("A + B > 1")
print("f3:", f3)
```

```
f3: [0 0 0 1]
```

Some examples of more complicated functions include:

```
g = bf.BooleanFunction("(A AND B) OR (NOT A AND C)")
h = bf.BooleanFunction("(x + y + z) % 2 == 0")
k = bf.BooleanFunction("(-1) * x + y + z > 0")

labels = ["g", "h", "k"]
bf.display_truth_table(g, h, k, labels=labels)
```

```
x0  x1  x2  |   g   h   k
-------------------------------------------------
0   0   0   |   0   1   0
0   0   1   |   1   0   1
0   1   0   |   0   0   1
0   1   1   |   1   1   1
1   0   0   |   0   0   0
1   0   1   |   0   1   0
1   1   0   |   1   1   0
1   1   1   |   1   0   1
```

1.3.3 Combining BooleanFunction objects

New Boolean functions can be constructed by combining existing ones using Boolean algebra operations. This is useful when building larger rules from simpler components.

Supported operations include:

- ~ NOT
- & AND

- | OR
- ^ XOR

```
a = bf.BooleanFunction("X + Y == 1")
b = bf.BooleanFunction("X OR Y")

not_a = ~a
a_and_b = a & b
a_or_b = a | b
a_xor_b = a ^ b

labels = ["a", "b", "~a", "a&b", "a|b", "a^b"]
bf.display_truth_table(a, b, not_a, a_and_b, a_or_b, a_xor_b, labels=labels)
```

```
x0   x1   |    a    b    ~a   a&b  a|b  a^b
-------------------------------------------------------------------
0    0    |    0    0    1    0    0    0
0    1    |    1    1    0    1    1    0
1    0    |    1    1    0    1    1    0
1    1    |    0    1    1    0    1    1
```

1.4 Attributes of BooleanFunction

Each `BooleanFunction` object has the following attributes:

attribute	type	description
`f`	`np.ndarray`	truth table (right side)
`n`	`int`	number of variables
`variables`	`np.ndarray`	variable names
`name`	`str`	optional name
`properties`	`dict`	cached properties

```
print("f.f:", f.f)
print("f.n:", f.n)
print("f.variables:", f.variables)
print("f.name:", f.name)
print("f.properties:", f.properties)
```

```
f.f: [0 0 0 1]
f.n: 2
f.variables: ['x0' 'x1']
f.name: f_AND
f.properties: {}
```

When a function is created from a truth table, variable names default to `x0, x1, ...`. When created from text, variable names are inferred.

```
print("f2.variables:", f2.variables)
print("f3.variables:", f3.variables)
print("g.variables:", g.variables)
print("h.variables:", h.variables)
```

```
f2.variables: ['A' 'B']
f3.variables: ['A' 'B']
```

```
g.variables: ['A' 'B' 'C']
h.variables: ['x' 'y' 'z']
```

The variable order is determined by first occurrence in the expression. See e.g.,

```
print(bf.BooleanFunction("(x + y + z) % 2 == 0").variables)
print(bf.BooleanFunction("(y + z + x) % 2 == 0").variables)
```

```
['x' 'y' 'z']
['y' 'z' 'x']
```

The variable order determines how the truth table is indexed. For example, if variables are sorted as [x,y,z], the entry in position i corresponds to the binary expansion of i over (x,y,z). E.g., row $i = 4$ corresponds to $x = 1, y = 0, z = 0$. Therefore, the same expression with a different variable order results in a different truth table ordering. This becomes important when combining functions inside networks or importing networks from text files. That said, it is all handled internally by BoolForge.

1.5 Basic properties of Boolean functions

We can inspect various properties of a Boolean function. The degree, i.e., the number of inputs, is readily available via 'f.n'. Other properties can be computed.

- 'is_constant()' checks if the function is constant,
- 'is_degenerate()' checks if the function contains non-essential variables,
- 'get_essential_variables()' provides the indices (Python: starting at 0!) of the essential variables,
- 'get_type_of_inputs()' describes the type of each input ('positive', 'negative', 'conditional', or 'non-essential').
- The Hamming weight is the number of 1s in the right side of the truth table.
- The bias is $\#\text{ones}/2^n$. It equals 0.5 for unbiased functions.
- The absolute bias is $|\#\text{ones} - \#\text{zeros}|/2^n$. It equals 1 for constant functions and 0 for unbiased functions.

```
print("Number of variables:", f.n)
print("Is constant?", f.is_constant())
print("Is degenerate?", f.is_degenerate())
print("Essential variables:", f.get_essential_variables())
print("Type of inputs:", f.get_type_of_inputs())
print("Hamming weight:", f.hamming_weight)
print("Bias:", f.bias)
print("Absolute bias:", f.absolute_bias)
```

```
Number of variables: 2
Is constant? False


Is degenerate? False
Essential variables: [0 1]
Type of inputs: ['positive' 'positive']
Hamming weight: 1
Bias: 0.25
Absolute bias: 0.5
```

You may repeat this for g and observe how the properties differ.

Conveniently, the .summary() method prints a human-readable overview of basic properties.

```
f = bf.BooleanFunction("(A and B) OR NOT C")
print(f.summary())
```

```
BooleanFunction
---------------
Number of variables:       3
Hamming Weight:            5
Bias:                      0.625
Absolute bias:             0.250
Variables:                 ['A', 'B', 'C']
```

If more advanced properties have already been computed, e.g., by `get_layer_structure()` or `get_type_of_inputs()`, they are also displayed. This is also the case if the optional keyword `compute_all` is set to True; default is False to avoid potentially time-consuming computations.

```
print(f.summary(compute_all=True)) #or simply print(f.summary(True))
```

```
BooleanFunction
---------------
Number of variables:       3
Hamming Weight:            5
Bias:                      0.625
Absolute bias:             0.250
Variables:                 ['A', 'B', 'C']
Activities:                ['0.250', '0.250', '0.750']
Average sensitivity:       0.417
InputTypes:                ['positive' 'positive' 'negative']
CanalizingDepth:           3
NumberOfLayers:            2
CanalizingInputs:          [0 0 0]
CanalizedOutputs:          [1 0 0]
CoreFunction:              [1]
OrderOfCanalizingVariables:[2 0 1]
LayerStructure:            [1, 2]
```

The more advanced properties displayed here (e.g., all properties related to canalization) are the subject of later tutorials.

1.6 Logical and polynomial representations

While Boolean functions are stored as truth tables, they can be expressed in logical and polynomial format.

```
print(f"Logical form of {f.name}:", f.to_logical(and_op=" \wedge ", or_op=" \vee ", not_op=" \neg")
    )
print(f"Polynomial form of {f.name}:", f.to_polynomial())
```

```
Logical form of : (( \negC)) \vee (A \wedge B)
Polynomial form of : (1 - A) * (1 - B) * (1 - C) + (1 - A) * B * (1 - C) + A * (1 - B) * (1 - C) +
    A * B * (1 - C) + A * B * C
```

In addition, a `BooleanFunction` object can be turned into `BooleanNode` object from the CANA package. This requires the optional `CANA` package to be installed.

```
cana_object = f.to_cana()
print(type(cana_object))
```

```
<class 'cana.boolean_node.BooleanNode'>
```

1.7 Summary

Before moving on to more advanced topics, here is a short summary of the fundamental ideas introduced in this tutorial:

1.7.1 Boolean functions

A Boolean function maps a set of binary inputs (0/1) to a single binary output. BoolForge represents Boolean functions internally by their truth table, i.e., the list of outputs in lexicographic order of the input combinations.

1.7.2 Representations of Boolean functions

Boolean functions can be created from:

- a truth table (a sequence of 0s and 1s of length 2^n for some n),
- a logical expression written in Python syntax,
- algebraic combinations of existing BooleanFunction objects using operations such as `+` (OR), `*` (AND), `^` (XOR), and other supported Boolean operations.

Each representation produces an equivalent internal truth-table-based object.

1.7.3 Variable names and ordering

BoolForge automatically infers variable names from the order of first appearance in expressions.
This order determines the indexing of the truth table and therefore affects how the function interacts with larger Boolean networks.

1.7.4 Basic properties of Boolean functions

BoolForge can compute structural properties, including:

- the number of variables (`n`),
- the Hamming weight (number of 1s in the truth table),
- absolute bias (imbalance between 0s and 1s),
- essential and non-essential variables,
- positive/negative influence of each input.

These properties help characterize the function's behavior and are used throughout later tutorials.

1.7.5 Conversions and interoperability

BoolForge supports conversion between representations (truth table, polynomial, and logical form) and is compatible with external packages such as CANA for advanced analysis.
This makes it easy to move between analytical frameworks and reuse models.

Together, these concepts provide the foundation for understanding canalization, random Boolean function generation, and eventually the construction and analysis of full Boolean networks.

1.8 Frequently Asked Questions

1.8.1 Why does the order of variables matter?

The order in which variables appear determines the ordering of the truth table. For a function with variables `[A, B, C]`, the entry at position $i \in \{0, 1, \ldots, 2^n - 1\}$ corresponds to the binary representation of i over `(A, B, C)`. For example, row 4 (i.e., the fifth row since Python starts indexing at 0) corresponds to $A = 1, B = 0, C = 0$.

If two equivalent expressions list variables in different orders, their truth tables will be indexed differently. See, for example,

```
print(bf.BooleanFunction('A and not B'))
print(bf.BooleanFunction('not B and A'))
```

```
[0 0 1 0]
[0 1 0 0]
```

To ensure reproducibility, always use consistent variable names and ordering.

1.8.2 How do I choose between defining a function via a truth table or via an expression?

Short answer: It does not matter. Both methods produce identical internal representations.

Slightly longer answer: Use a *textual expression* if:

- you know the natural logical description of your function (e.g., `A and B`),
- the function is part of a Boolean network stored in some text file.

Use a *truth table* if:

- you generated the table programmatically (e.g., using `bf.random_function`).

1.8.3 What is the difference between `get_type_of_inputs()` and monotonicity?

The method `get_type_of_inputs()` classifies each input variable individually, i.e., it describes how an increase in the variable can affect the function output:

- positive: the function value increases at least sometimes but never decreases,
- negative: the function value decreases at least sometimes but never increases,
- conditional: both positive and negative,
- non-essential: the function value never changes.

Monotonicity, by contrast, is a *global property* of the Boolean function. A function is monotone if *none* of its essential variables are conditional.

A function can therefore be non-monotone even if some individual inputs affect it in a monotone manner.

1.8.4 Quick Reference

Task	Example
Create from truth table	`BooleanFunction([0, 0, 0, 1])`
Create from expression	`BooleanFunction("A and B")`
Combine with operations	f & g, f | g, ~f, f ˆ g
Check properties	`f.n`, `f.is_constant()`, `f.is_degenerate()`
Get variable names	`f.variables`
Convert representations	`f.to_logical()`, `f.to_polynomial()`

2 Advanced Concepts for Boolean Functions

Understanding the structure of a Boolean function is essential for analyzing the behavior of the Boolean networks they define. In this tutorial, we move beyond the basics of `BooleanFunction` and explore three core concepts:

- symmetries among inputs
- activities of inputs
- average sensitivity of a Boolean function

These quantities are tied to redundancy, robustness, and dynamical behavior – concepts that will play a central role in later tutorials on canalization and network dynamics.

2.1 What you will learn

In this tutorial you will learn how to:

- identify symmetry groups of Boolean functions,
- compute activities and sensitivities,
- choose between exact computation and Monte Carlo estimation,
- interpret these quantities in terms of robustness and redundancy.

2.2 Setup

```
import boolforge as bf
import numpy as np
```

2.3 Symmetries in Boolean functions

In gene regulation, symmetric variables might represent redundant transcription factor binding sites or functionally equivalent repressors. Identifying symmetries can:

- Reduce model complexity
- Suggest evolutionary mechanisms (gene duplication)
- Identify potential drug targets (symmetric inputs may compensate)

A symmetry of a Boolean function is a permutation of input variables that does *not* change its output.

- Inputs in the same symmetry group can be swapped freely.
- Inputs in different groups cannot.

The following three Boolean functions exhibit full, partial, and no symmetry.

```

# Fully symmetric (parity / XOR)
f = bf.BooleanFunction("(x0 + x1 + x2) % 2")


# Partially symmetric
g = bf.BooleanFunction("x0 | (x1 & x2)")


# No symmetry
h = bf.BooleanFunction("x0 | (x1 & ~x2)")

labels = ["f", "g", "h"]
bf.display_truth_table(f, g, h, labels=labels)

for func, label in zip([f, g, h], labels):
```

```
    print(f"Symmetry groups of {label}:")
    for group in func.get_symmetry_groups():
        print("  ", func.variables[np.array(group)])
    print()
```

```
x0  x1  x2  |   f   g   h
-------------------------------------------------
0   0   0   |   0   0   0
0   0   1   |   1   0   0
0   1   0   |   1   0   1
0   1   1   |   0   1   0
1   0   0   |   1   1   1
1   0   1   |   0   1   1
1   1   0   |   0   1   1
1   1   1   |   1   1   1
Symmetry groups of f:
   ['x0' 'x1' 'x2']

Symmetry groups of g:
   ['x0']
   ['x1' 'x2']

Symmetry groups of h:
   ['x0']
   ['x1']
   ['x2']
```

Interpretation:

- `f` is fully symmetric: all variables are interchangeable.
- `g` has partial symmetry: `x1` and `x2` are equivalent but `x0` is distinct.
- `h` has no symmetries: all inputs play unique roles.

These patterns foreshadow the concepts of canalization, and specifically canalizing layers, explored in later tutorials.

2.4 Degenerate functions

A function is *degenerate* if one or more inputs do not matter at all.

```
print("f.is_degenerate()", f.is_degenerate())
k = bf.BooleanFunction("(x AND y) OR x")
print("k.is_degenerate()", k.is_degenerate())
```

```
f.is_degenerate() False
k.is_degenerate() True
```

Detecting degeneracy is NP-hard in general. However even at relatively low degree, such functions are extremely rare unless intentionally created.

We can also identify the specific variables that cause a function to be degenerate.

```
nonessential = [
    str(v) for v, t in zip(k.variables, k.get_type_of_inputs())
    if t == "non-essential"
]

print("Non-essential variables:", nonessential)
```

```
Non-essential variables: ['y']
```

2.5 Activities and sensitivities

Activities and sensitivity quantify how much each input affects the output of a Boolean function.

2.5.1 Activity

The activity of input x_i is the probability that flipping x_i changes the function's output:

$$a(f, x_i) = \Pr[f(\mathbf{x}) \neq f(\mathbf{x} \oplus e_i)],$$

where $e_i = (0, \ldots, 0, 1, 0, \ldots, 0)$ is the ith unit vector.

- If $a = 1$: the variable always matters.
- If $a = 0$: the variable is irrelevant (degenerate).
- In large random Boolean functions, $a \approx 0.5$ for all variables.

2.5.2 Average sensitivity

The *average sensitivity* of a Boolean function describes how sensitive its output is to changes in its inputs, specifically to a random single-bit flip [Shmulevich and Kauffman, 2004]. The (unnormalized) average sensitivity is the sum of all its activities:

$$S(f) = \sum_i a(f, x_i).$$

Division by n yields the *normalized average sensitivity* $s(f)$, which can be readily compared between functions of different degree n:

$$s(f) = \frac{S(f)}{n}.$$

Interpretation:

In Boolean network theory, the mean normalized average sensitivity $s(f)$ determines how perturbations tend to propagate through the system.

- If $s(f) < 1$, perturbations tend to die out (*ordered regime*).
- If $s(f) > 1$, perturbations typically amplify (*chaotic regime*).
- The boundary $s(f) = 1$ defines the *critical regime*.

The critical regime is believed to characterize many biological networks (see later tutorials and also Daniels et al. [2018]). It represents a balance between order and chaos. Operating at this "edge of chaos" may optimize information processing and evolvability.

2.5.3 Exact vs Monte Carlo computation

- Exact (`exact=True`) computation enumerates all 2^n states; feasible for small n.
- Monte Carlo (`exact=False`, default) simulation approximates using random samples; scalable to large n.

Computational cost guide:

- Exact methods: $O(2^n)$ time and space, where n = number of inputs.
- Monte Carlo: $O(k)$ time, where k = number of samples.

Recommendation:

- $n \leq 10$: Use exact methods (fast, deterministic)
- $10 < n \leq 20$: Use exact if possible, Monte Carlo if repeated computation needed
- n > 20: Use Monte Carlo (exact is infeasible)

2.5.4 Computing activities and sensitivities

To investigate how to compute the activities and the average sensitivity in `BoolForge`, we work with the linear function `f` from above, as well as with the function `g`.

```
exact = True
normalized = True

print("Activities of f:", f.get_activities(exact=exact))
print("Activities of g:", g.get_activities(exact=exact))

print("Normalized average sensitivity of f:", f.get_average_sensitivity(exact=exact,
                                                                normalized=normalized))
print("Normalized average sensitivity of g:", g.get_average_sensitivity(exact=exact,
                                                                normalized=normalized))
```

```
Activities of f: [1. 1. 1.]
Activities of g: [0.75 0.25 0.25]
Normalized average sensitivity of f: 1.0
Normalized average sensitivity of g: 0.4166666666666667
```

Interpretation:

- For `f` (XOR), flipping any input always flips the output, so $s(f) = 1$.
- For `g`, x_0 influences the output more often than x_1 or x_2. 75% of x_0 flips and 25% of x_1 or x_2 flips change the output of `g`. Thus, the normalized average sensitivity of `g` is $\frac{1}{3} * 75\% + \frac{2}{3}25\% = \frac{5}{12}$.

This unequal influence is a precursor to canalization, a property investigated in depth in the next tutorial.

Exact computation is infeasible for large n, so Monte Carlo simulation must be used.

When generating such a large function randomly (see Tutorial 4), it is not recommended to require that all inputs are essential, as (i) this is almost certainly the case anyways (the probability that an n-input function does not depend on input x_i is given $1/2^{n-1}$), and (ii) checking for input degeneracy is NP-hard (i.e., very computationally expensive). In this specific case, we thus suggest diverging from BoolForge's default and setting `allow_degenerate_functions=True`. You find more on this and the `random_function` method in Tutorial 4.

```
exact = False
n = 25

h = bf.random_function(n=n, allow_degenerate_functions=True)

activities = h.get_activities(exact=exact)
print(f"Mean activity: {np.mean(activities):.4f}")
print(
    f"Normalized average sensitivity: {h.get_average_sensitivity(exact=exact):.4f}"
)
```

```
Mean activity: 0.4998
Normalized average sensitivity: 0.5006
```

Interpretation:

Random Boolean functions satisfy:

- mean activity ≈ 0.5,
- normalized average sensitivity ≈ 0.5.

Thus, the results for `h` align with known theoretical results. More generally, random Boolean function results define the typical behavior against which biological functions can be compared (see Tutorial 5).

2.6 Summary

In this tutorial you learned:

- how to compute symmetry groups,
- how to test for input degeneracy,
- how to compute activities and sensitivities,
- how these quantities relate to robustness and structure.

These concepts provide essential foundations for understanding

- canalization, the core concept of Tutorial 3,
- and the robustness of Boolean networks, explored in Tutorial 8.

3 Canalization

Canalization is a key property of biological Boolean functions that confers robustness: when a canalizing variable takes its canalizing value, the output is determined regardless of other inputs. This "buffering" mechanism is thought to protect organisms from genetic and environmental perturbations.

First described by C.H. Waddington in 1942 in developmental biology [Waddington, 1942], canalization has since been formalized in Boolean network theory [Kauffman, 1974], and found to be abundantly prevalent in empirically-derived gene regulatory networks [Kadelka et al., 2024].

3.1 What you will learn

In this tutorial you will:

- determine if a Boolean function is canalizing, k-canalizing, and nested canalizing,
- compute the canalizing layer structure of any Boolean function,
- compute properties related to collective canalization, such as canalizing strength, effective degree and input redundancy.

3.2 Setup

```
import boolforge as bf
import matplotlib.pyplot as plt
```

3.3 Canalizing variables and layers

A Boolean function $f(x_1, \dots, x_n)$ is *canalizing* if there exists at least one *canalizing variable* x_i and a *canalizing input value* $a \in \{0, 1\}$ such that

$$f(x_1, \dots, x_i = a, \dots, x_n) = b,$$

where $b \in \{0, 1\}$ is a constant, the *canalized output* [Kauffman, 1974].

A Boolean function is *k-canalizing* if it has at least k conditionally canalizing variables. This is checked recursively: after fixing a canalizing variable x_i to its non-canalizing input value $\bar{a}$, the subfunction $f(x_1, \dots, x_{i-1}, x_{i+1}, \dots, x_n)$ must itself contain another canalizing variable, and so on. For a given function, the maximal possible value of k is defined as its *canalizing depth* [Layne et al., 2012]. If all variables are conditionally canalizing (i.e., if the canalizing depth is n), the function is called a *nested canalizing* function (*NCF*) [He and Macauley, 2016]. Biological networks are heavily enriched for NCFs as we explore in Tutorial 11.

Per He and Macauley [2016], any Boolean function can be decomposed into a unique standard monomial form by recursively identifying and removing all conditionally canalizing variables (this set of variables is called a *canalizing layer*). Each variable of a Boolean function appears in exactly one layer, or (if it is not conditionally canalizing) it is part of the non-canalizing core function that has to be evaluated only if all conditionally canalizing variables receive their non-canalizing input value. The *canalizing layer structure* $[k_1, \dots, k_r]$ describes the number of variables in each canalizing layer [Dimitrova et al., 2022]. We thus have $r \geq 0$, $k_i \geq 1$ and $k_1 + \cdots + k_r$.

In the following code, we define four 3-input functions with different canalizing properties.

```

# Non-canalizing XOR function
f = bf.BooleanFunction("(x0 + x1 + x2) % 2")


# 1-canalizing function
```

```
g = bf.BooleanFunction("(x0 | (x1 & x2 | !x1 & !x2)) % 2")


# Nested canalizing function with all variables in one layer
h = bf.BooleanFunction("~x0 & x1 & x2")


# Nested canalizing function with two canalizing layers
k = bf.BooleanFunction("x0 | (x1 & x2)")

labels = ["f", "g", "h", "k"]
bf.display_truth_table(f, g, h, k, labels=labels)
```

```
x0  x1  x2  |   f   g   h   k
---------------------------------------------------------
0   0   0   |   0   1   0   0
0   0   1   |   1   0   0   0
0   1   0   |   1   0   0   0
0   1   1   |   0   1   1   1
1   0   0   |   1   1   0   1
1   0   1   |   0   1   0   1
1   1   0   |   0   1   0   1
1   1   1   |   1   1   0   1
```

3.3.1 Canalizing depth and nested canalization

For each function, we can determine whether it is canalizing and/or nested canalizing. This is determined by the canalizing depth (the number of conditionally canalizing variables), which we can also directly compute. As a reminder, an n-input function is canalizing if its canalizing depth is non-zero and nested canalizing if its canalizing depth equals n.

```
for func, label in zip([f, g, h, k], labels):
    depth = func.get_canalizing_depth()
    print(f"Canalizing depth of {label}: {depth}")

    print(f"{label} is canalizing:", func.is_canalizing())
    print(f"{label} is nested canalizing:", func.is_k_canalizing(k=func.n))
    print()
```

```
Canalizing depth of f: 0
f is canalizing: False
f is nested canalizing: False

Canalizing depth of g: 1
g is canalizing: True
g is nested canalizing: False

Canalizing depth of h: 3
h is canalizing: True
h is nested canalizing: True

Canalizing depth of k: 3
k is canalizing: True
k is nested canalizing: True
```

3.3.2 Canalizing layer structure

The full canalizing layer structure includes canalizing input values, canalized output values, the order of canalizing variables, the layer structure, and the remaining non-canalizing core function.

```
for func, label in zip([f, g, h, k], labels):
    info = func.get_layer_structure()
    print(f"Canalizing input values of {label}: {info['CanalizingInputs']}")
    print(f"Canalized output values of {label}: {info['CanalizedOutputs']}")
    print(f"Order of canalizing variables of {label}: {info['OrderOfCanalizingVariables']}")
    print(f"Layer structure of {label}: {info['LayerStructure']}")
    print(f"Number of layers of {label}: {info['NumberOfLayers']}")
    print(f"Core function of {label}: {info['CoreFunction']}")
    print()
```

```
Canalizing input values of f: []
Canalized output values of f: []
Order of canalizing variables of f: []
Layer structure of f: []
Number of layers of f: 0
Core function of f: [0 1 1 0 1 0 0 1]

Canalizing input values of g: [1]
Canalized output values of g: [1]
Order of canalizing variables of g: [0]
Layer structure of g: [1]
Number of layers of g: 1
Core function of g: [1 0 0 1]

Canalizing input values of h: [1 0 0]
Canalized output values of h: [0 0 0]
Order of canalizing variables of h: [0 1 2]
Layer structure of h: [3]
Number of layers of h: 1
Core function of h: [1]

Canalizing input values of k: [1 0 0]
Canalized output values of k: [1 0 0]
Order of canalizing variables of k: [0 1 2]
Layer structure of k: [1, 2]
Number of layers of k: 2
Core function of k: [1]
```

Consider, for example, the output for `k`. The canalizing input values corresponding to x_0, x_1, x_2 are $1, 0, 0$, respectively, with the same canalized outputs. That is,

- Layer 1 contains x_0 (if $x_0 = 1$, then $k = 1$, regardless of x_1 and x_2)
- Layer 2 contains x_1 and x_2 (given $x_0 = 0$, if $x_1 = 0$ or $x_2 = 0$, then $k = 0$)

3.4 Collective canalization

Collective canalization treats canalization as a property of the function rather than individual variables [Reichhardt and Bassler, 2007]. Individual canalization asks: "Which *single* variables can determine output?" Collective canalization asks: "Which *sets* of variables can determine output?"

A Boolean function is *k-set canalizing* if there exists a set of k variables whose fixed values determine the output irrespective of the remaining inputs.

Consider, for example, the function $k(x_0, x_1, x_2) = x_0 \vee (x_1 \wedge x_2)$, defined above. This function is 2-set canalizing because

- $\{x_0, x_1\}$ can determine the output: if $(x_0, x_1) = (1, 0)$, $k = 1$ (x_2 irrelevant), or
- $\{x_1, x_2\}$ can determine the output: if $(x_1, x_2) = (1, 1)$, $k = 1$ (x_0 irrelevant)

The proportion of such k-sets, the *k-set canalizing proportion* denoted $P_k(f)$, is an important summary statistic. It is fairly obvious that

- nested canalizing functions of a single layer such as `h` are the non-degenerate functions with largest k-set canalizing proportion $P_k(f) = 1 - 1/2^k$, and
- $P_{k-1}(f) \leq P_k(f)$, i.e., more knowledge about a function's inputs cannot result in less knowledge about its output,
- the $n-1$-set canalizing proportion $P_{n-1}(f)$ is 1 minus the function's normalized average sensitivity.

We can compute the k-set canalizing proportions for the four 3-input functions:

```
for func, label in zip([f, g, h, k], labels):
    print(f"1-set canalizing proportion of {label}: {func.get_kset_canalizing_proportion(k=1)}")
    print(f"2-set canalizing proportion of {label}: {func.get_kset_canalizing_proportion(k=2)}")
    print(f"Normalized average sensitivity of {label}:"
          f"{func.get_average_sensitivity(exact=True, normalized=True)}")
    print(f"3-set canalizing proportion of {label}: {func.get_kset_canalizing_proportion(k=3)}")
    print()
```

```
1-set canalizing proportion of f: 0.0
2-set canalizing proportion of f: 0.0
Normalized average sensitivity of f:1.0
3-set canalizing proportion of f: 1.0

1-set canalizing proportion of g: 0.16666666666666666
2-set canalizing proportion of g: 0.5
Normalized average sensitivity of g:0.5
3-set canalizing proportion of g: 1.0

1-set canalizing proportion of h: 0.5
2-set canalizing proportion of h: 0.75
Normalized average sensitivity of h:0.25
3-set canalizing proportion of h: 1.0

1-set canalizing proportion of k: 0.16666666666666666
2-set canalizing proportion of k: 0.5833333333333334
Normalized average sensitivity of k:0.4166666666666667
3-set canalizing proportion of k: 1.0
```

3.4.1 Canalizing strength

The *canalizing strength* summarizes collective canalization as a weighted average of the k-set canalizing proportions [Kadelka et al., 2023]. It ranges from:

- 1 for maximally canalizing non-degenerate functions (namely, nested canalizing functions of a single canalizing layer such as `h`),
- 0 for linear functions such as `f`,

For all other non-degenerate Boolean functions it is within $(0, 1)$.

It helps to consider the canalizing strength as a probability: Given that I know a random number of function inputs (drawn uniformly at random from $1, \ldots, n-1$), how likely am I to already know the function output?

```
for func, label in zip([f, g, h, k], labels):
    strength = func.get_canalizing_strength()
    print(f"Canalizing strength of {label}: {strength}")
    print()
```

```
Canalizing strength of f: 0.0

Canalizing strength of g: 0.5

Canalizing strength of h: 1.0

Canalizing strength of k: 0.5555555555555556
```

3.4.2 Distribution of canalizing strength

An enumeration of all non-degenerate 3-input Boolean functions reveals the distribution of the canalizing strength. Note that this brute-force code can also run (in less than a minute) for all $2^{2^4} = 2^{16} = 65,536$ 4-input functions but will take days for all $2^{2^5} = 2^{32} = 4,294,967,296$ 5-input functions.

```
n = 3
all_functions = bf.get_left_side_of_truth_table(2 n)

canalizing_strengths = []
for binary_vector in all_functions:
    func = bf.BooleanFunction(f=binary_vector)
    if not func.is_degenerate():
        canalizing_strengths.append(func.get_canalizing_strength())

fig, ax = plt.subplots()
ax.hist(canalizing_strengths, bins=50)
ax.set_xlabel("Canalizing strength")
ax.set_ylabel("Count")
plt.show()
```

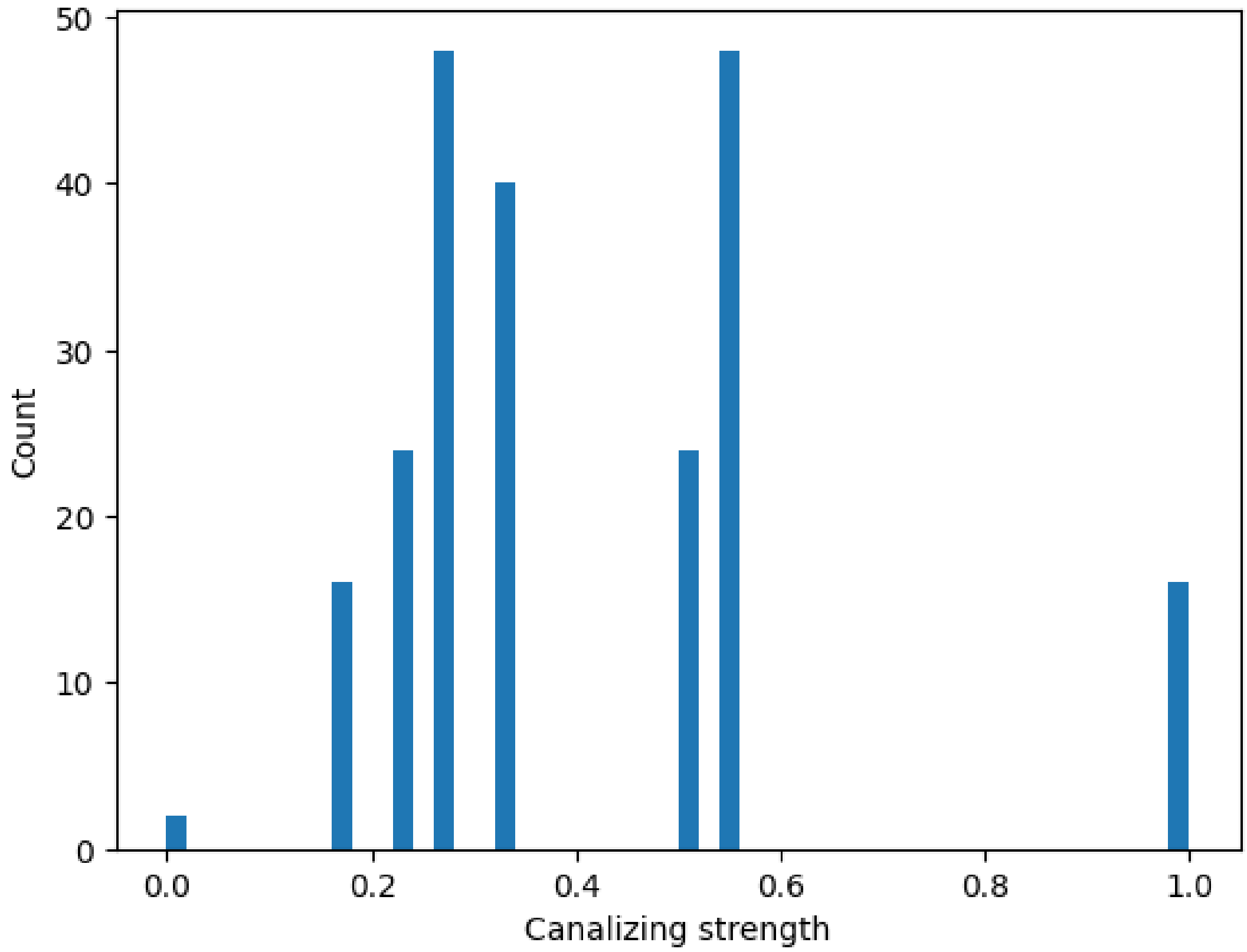

3.5 Canalization as a measure of input redundancy

Canalization, symmetry and redundancy are related concepts. A highly symmetry Boolean function with few (e.g., one) symmetry groups exhibits high input redundancy and is on average more canalizing, irrespective of the measure of canalization. Recently, it was shown that almost all Boolean functions (except the linear functions) exhibit some level of *input redundancy* [Gates et al., 2021]. The input redundancy of a variable is defined as 1 minus its *edge effectiveness*, which describes the proportion of times that this variable is needed to determine the output of the function. Edge effectiveness is very similar to the activity of a variable but is not the same (the difference is defined as *excess canalization*). The sum of all edge effectiveness values of the inputs of a function is known as its *effective degree*. The average input redundancy serves as a measure of the canalization in a function.

`BoolForge` can compute all these quantities. To use this functionality, the optional `CANA` package must be installed (`pip install cana` or `pip install boolforge[cana]`). To exemplify this, reconsider the four 3-input functions from above.

```
for func, label in zip([f, g, h, k], labels):
    edge_eff = func.get_edge_effectiveness()
    activities = func.get_activities()
    effective_degree = func.get_effective_degree()
    input_redundancy = func.get_input_redundancy()

    print(f"Edge effectiveness of {label}: {edge_eff}")
    print(f"Activities of {label}: {activities}")
    print(f"Excess canalization of {label}: {edge_eff - activities}")
    print(f"Effective degree of {label}: {effective_degree}")
    print(f"Average edge effectiveness of {label}: {effective_degree / func.n}")
    print(f"Normalized input redundancy of {label}: {input_redundancy}")
    print()
```

```
Edge effectiveness of f: [1.0, 1.0, 1.0]
Activities of f: [1. 1. 1.]
Excess canalization of f: [0. 0. 0.]
Effective degree of f: 3.0
Average edge effectiveness of f: 1.0
Normalized input redundancy of f: 0.0

Edge effectiveness of g: [0.625, 0.625, 0.625]
Activities of g: [0.5 0.5 0.5]
Excess canalization of g: [0.125 0.125 0.125]
Effective degree of g: 1.875
Average edge effectiveness of g: 0.625
Normalized input redundancy of g: 0.375

Edge effectiveness of h: [0.41666666666666663, 0.41666666666666663, 0.41666666666666663]
Activities of h: [0.25 0.25 0.25]
Excess canalization of h: [0.16666667 0.16666667 0.16666667]
Effective degree of h: 1.25
Average edge effectiveness of h: 0.4166666666666667
Normalized input redundancy of h: 0.5833333333333334

Edge effectiveness of k: [0.8125, 0.375, 0.375]
Activities of k: [0.75 0.25 0.25]
Excess canalization of k: [0.0625 0.125  0.125 ]
Effective degree of k: 1.5625
Average edge effectiveness of k: 0.5208333333333334
Normalized input redundancy of k: 0.4791666666666667
```

3.6 Summary

In this tutorial you learned how to:

- compute canalizing depth and identify nested canalizing functions,
- compute the canalizing layer structure and interpret layers and core functions,
- quantify collective canalization via k-set canalizing proportions,
- summarize canalization via canalizing strength,
- relate canalization to redundancy-based measures such as edge effectiveness.

Canalization provides a structural explanation for why many biological Boolean rules are robust to perturbations.

Next steps: Subsequent tutorials will explore random Boolean functions with prescribed canalization properties and the impact of canalization on Boolean network dynamics and robustness.

4 Random Boolean function generation

This tutorial focuses on the random generation of Boolean functions with prescribed properties, enabling large-scale computational studies.

Controlled random Boolean function generation enables:

1. Null model comparisons: Are biological regulatory rules special?
2. Ensemble studies: How do structural properties affect dynamical properties?
3. Theoretical predictions: Derive expected values for function properties

4.1 What you will learn

In this tutorial you will learn how to generate random Boolean functions with:

- specified canalizing properties (depth, layer structure),
- bias, absolute bias, or a specific Hamming weight,
- linearity constraints,
- degeneracy constraints.

It is strongly recommended to complete the previous tutorials first.

4.2 Setup

```
import boolforge as bf
import numpy as np
import matplotlib.pyplot as plt
```

4.3 Generating random Boolean functions

The function `random_function(n, *args)` generates a random n-input Boolean function subject to optional constraints. By default, it generates a *non-degenerate* function, meaning that all variables are essential.

```
n = 3
f = bf.random_function(n)

bf.display_truth_table(f, labels="f_random_non_degenerate")

print("Is f degenerate?", f.is_degenerate())
print("Activities of f:", f.get_activities(exact=True))
print("Edge effectiveness of f:", f.get_edge_effectiveness())
```

```
x0  x1  x2  |   f_random_non_degenerate
-------------------------------------------------------
0   0   0   |   0
0   0   1   |   0
0   1   0   |   1
0   1   1   |   1
1   0   0   |   1
1   0   1   |   0
1   1   0   |   0
1   1   1   |   1
Is f degenerate? False
Activities of f: [0.5 1.  0.5]
Edge effectiveness of f: [0.625, 1.0, 0.625]
```

The rest of this tutorial describes the various constraints. Each constraint defines a specific family of n-input Boolean functions, from which `random_function(n,*args)` samples *uniformly at random*. That is, each function satisfying a given set of constraints is selected with equal probability.

4.4 Parity functions

Setting `parity=True` generates *parity* functions, also known as non-degenerate *linear* functions.

```
f = bf.random_function(n, parity=True)

bf.display_truth_table(f, labels="f_linear")

print("Activities:", f.get_activities(exact=True))
print("Edge effectiveness:", f.get_edge_effectiveness())
print("Normalized average sensitivity:", f.get_average_sensitivity(exact=True))
print("Canalizing strength:", f.get_canalizing_strength())
```

```
x0  x1  x2  |   f_linear
----------------------------------------
0   0   0   |   1
0   0   1   |   0
0   1   0   |   0
0   1   1   |   1
1   0   0   |   0
1   0   1   |   1
1   1   0   |   1
1   1   1   |   0
Activities: [1. 1. 1.]
Edge effectiveness: [1.0, 1.0, 1.0]
Normalized average sensitivity: 1.0
Canalizing strength: 0.0
```

Parity functions are the only Boolean functions with activity 1 (for all variables), normalized average sensitivity 1 and canalizing strength 0.

4.5 Functions with prescribed canalizing properties

If `parity=False` (default), canalizing properties can be specified via `layer_structure` and `depth`.

4.5.1 Functions with prescribed canalizing layer structure

The canalizing layer structure can be specified via `layer_structure`. This vector describes the number of conditionally canalizing variables in each layer of the randomly generated function.

- If the optional argument `exact_depth=True` (default is False), then `layer_structure` describes the *exact* layer structure, i.e., the core function cannot be canalizing.
- If `exact_depth=False` (the default), it is possible that the core function is canalizing (meaning that the last described layer in `layer_structure` may contain more conditionally canalizing variables, or that there are additional canalizing layers).

Before generating any random function, `random_function()` goes through a number of checks ensuring that the provided optional arguments make sense. For example, it checks that the provided layer structure $(k_1, \ldots, k_r)$ satisfies

- $k_i \geq 1$,
- $k_1 + \cdots + k_r \leq n$, and
- if $k_1 + \cdots + k_r = n$, then $k_r \geq 2$ because the last layer of a nested canalizing function must always contain two or more variables.

```
f = bf.random_function(n, layer_structure=[1])
g = bf.random_function(n, layer_structure=[1], exact_depth=True)
h = bf.random_function(n, layer_structure=[3])
k = bf.random_function(n, layer_structure=[1, 2])
```

```
labels = ["f", "g", "h", "k"]
bf.display_truth_table(f, g, h, k, labels=labels)

for func, label in zip([f, g, h, k], labels):
    info = func.get_layer_structure()
    print(f"Canalizing depth of {label}: {func.get_canalizing_depth()}")
    print(f"Layer structure of {label}: {info['LayerStructure']}")
    print(f"Number of layers of {label}: {info['NumberOfLayers']}")
    print(f"Core function of {label}: {info['CoreFunction']}")
    print()
```

```
x0  x1  x2  |   f   g   h   k
---------------------------------------------------------
0   0   0   |   1   1   1   1
0   0   1   |   1   0   1   1
0   1   0   |   0   0   1   1
0   1   1   |   1   1   1   1
1   0   0   |   1   1   1   0
1   0   1   |   1   1   0   1
1   1   0   |   1   1   1   0
1   1   1   |   1   1   1   0
Canalizing depth of f: 3
Layer structure of f: [3]
Number of layers of f: 1
Core function of f: [0]

Canalizing depth of g: 1
Layer structure of g: [1]
Number of layers of g: 1
Core function of g: [1 0 0 1]

Canalizing depth of h: 3
Layer structure of h: [3]
Number of layers of h: 1
Core function of h: [0]

Canalizing depth of k: 3
Layer structure of k: [1, 2]
Number of layers of k: 2
Core function of k: [1]
```

Repeated evaluation of this block of code shows that the canalizing depth of `f` is either 1 or 3 (note that a canalizing depth of $n-1$ is never possible for a non-degenerate function). On the contrary, the canalizing depth of `g` is always 1 because we set `exact_depth=True`. The 2-input core function of `g` is one of the two parity functions, each with 50% probability. Likewise, the core function for the other functions is simply [0] or [1], each with 50% probability. Functions `h` and `k` are nested canalizing, i.e., their canalizing depth is 3. Their layer structure is exactly as specified.

4.5.2 Functions with prescribed canalizing depth

If we do not care about the specific layer structure but only about the canalizing depth, we specify the optional argument `depth` instead of `layer_structure`.

```
# any function has at least canalizing depth 0 so this is the same as bf.random_function(n)
f = bf.random_function(n,depth=0)
```

```python
# a random non-canalizing function
g = bf.random_function(n,depth=0,exact_depth=True)


# a random canalizing function
h = bf.random_function(n,depth=1)


# a random nested canalizing function
k = bf.random_function(n,depth=n)

labels = ["f", "g", "h", "k"]
bf.display_truth_table(f, g, h, k, labels=labels)

for func, label in zip([f, g, h, k], labels):
    print(f"Canalizing depth of {label}: {func.get_canalizing_depth()}")
    print()
```

```
x0  x1  x2  |   f   g   h   k
---------------------------------------------------------
0   0   0   |   0   1   0   1
0   0   1   |   1   0   0   1
0   1   0   |   0   0   0   0
0   1   1   |   0   0   0   1
1   0   0   |   0   0   1   1
1   0   1   |   1   1   1   1
1   1   0   |   0   1   1   0
1   1   1   |   1   0   0   0
Canalizing depth of f: 3

Canalizing depth of g: 0

Canalizing depth of h: 3

Canalizing depth of k: 3
```

Repeated evaluation of this block of code shows that the canalizing depth of `f` can be 0, 1, or 3. Note that specifying `depth=0` without `exact_depth=True` does not restrict the space of functions at all. On the contrary, the canalizing depth of `g` is always 0 (i.e., g does not contain any canalizing variables) because we set `exact_depth=True`. Function `h` is canalizing and may be nested canalizing (because we specified that the minimal canalizing depth is 1), and `k` is always nested canalizing (i.e., it has canalizing depth $n = 3$).

We remember: If `exact_depth=True`, `depth` is interpreted as exact canalizing depth. Otherwise (default), `depth` is interpreted as minimal canalizing depth. For example,

- `depth=1`: "At least 1-canalizing" (could be 2,3,...,n-canalizing)
- `depth=1, exact_depth=True`: "Exactly 1-canalizing" (not 2,3,...,n-canalizing)

4.6 Allowing degenerate functions

It is possible that an n-input Boolean function does not depend on all its variables. For example, the function $f(x, y) = x$ depends on x but not on y. *By default, such degenerate functions are never generated by `random_function()`.* To enable the generation of possibly degenerate functions, we set `allow_degenerate_functions=True`. Although hardly of any practical value, we can even restrict the random generation to degenerate functions only, using `bf.generate.random_degenerate_function(n,*args)`.

Note: When generating random canalizing functions, the value of `allow_degenerate_functions` is ignored.

The non-canalizing core function is constructed to depend on all of its variables so that the number of essential variables equals the specified value. Otherwise degeneracy would reduce the number of essential variables and confound analyses of random Boolean networks, especially when the degree is small.

Since degenerate functions occur much more frequently at low degree, we set `n=2`, generate a large number of random, possibly degenerate functions and compare a histogram of the observed number of essential variables to the expected proportions.

```
n = 2
n_simulations = 10000

count_essential = np.zeros(n + 1, dtype=int)

for _ in range(n_simulations):
    f = bf.random_function(n, allow_degenerate_functions=True)
    count_essential[f.get_number_of_essential_variables()] += 1

expected = np.array([2 / 16, 4 / 16, 10 / 16])

x = np.arange(n + 1)
width = 0.4

fig, ax = plt.subplots()
ax.bar(x - width / 2, count_essential / n_simulations, width=width, label="observed")
ax.bar(x + width / 2, expected, width=width, label="expected")
ax.legend(frameon=False)
ax.set_xticks(x)
ax.set_xlabel("Number of essential variables")
ax.set_ylabel(f"Proportion of {n}-input functions")

print("Error:", count_essential / n_simulations - expected)
plt.show()
```

```
Error: [-0.0068 -0.0075  0.0143]
```

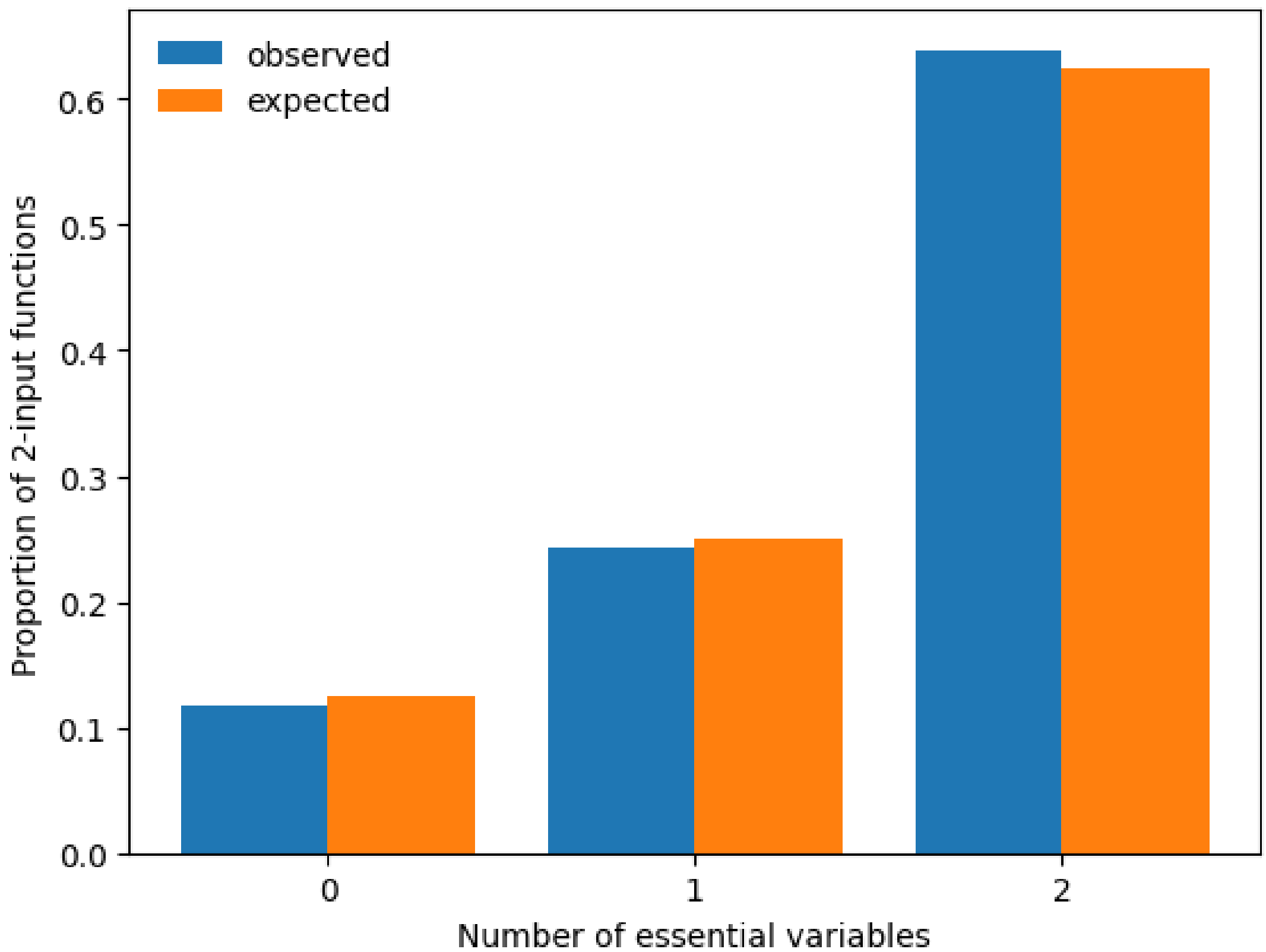

4.7 Functions with prescribed Hamming weight

The Hamming weight of a Boolean function is the number of ones in its truth table. BoolForge allows for the generation of random n-input functions with a specific Hamming weight $w \in \{0, 1, \ldots, 2^n\}$. The additional optional parameters `allow_degenerate_functions` and `exact_depth` specify whether degenerate and canalizing functions are allowed. By default, canalizing functions are allowed, while degenerate functions are not. Since all functions with Hamming weight $w \in \{0, 1, 2^n-1, 2^n\}$ are canalizing, we require $2 \leq w \leq 2^n-2$ whenever canalizing functions are not permissible (i.e., whenever `exact_depth=True`).

```
n = 3

f = bf.random_function(n, hamming_weight=5)
g = bf.random_function(n, hamming_weight=5, exact_depth=True)
h = bf.random_function(n, hamming_weight=2, allow_degenerate_functions=True)

labels = ["f", "g", "h"]
bf.display_truth_table(f, g, h, labels=labels)

for func, label in zip([f, g, h], labels):
    print(f"Hamming weight of {label}: {func.hamming_weight}")
    print(f"Canalizing depth of {label}: {func.get_canalizing_depth()}")
    print(f"Number of essential variables of {label}: {func.get_number_of_essential_variables()}")
    print()
```

```
x0  x1  x2  |   f   g   h
-------------------------------------------------
0   0   0   |   0   0   0
0   0   1   |   0   1   0
0   1   0   |   1   1   0
```

```
0   1   1   |   1   0   0
1   0   0   |   1   1   1
1   0   1   |   1   0   1
1   1   0   |   0   1   0
1   1   1   |   1   1   0
Hamming weight of f: 5
Canalizing depth of f: 0
Number of essential variables of f: 3

Hamming weight of g: 5
Canalizing depth of g: 0
Number of essential variables of g: 3

Hamming weight of h: 2
Canalizing depth of h: 2
Number of essential variables of h: 2
```

4.8 Biased and absolutely biased functions

While specifying the Hamming weight fixes the exact number of 1s in the truth table of a generated function, specifying the bias or absolute bias acts slightly differently. The bias p describes the probability of selecting a 1 at any position in the truth table and can be modified using the optional argument `bias`. Instead of specifying the bias, the absolute bias may also be specified. Unbiased functions contain an equal number of ones and zeros in their truth table and have an absolute bias of 0, the default. If, for example, we set `absolute_bias=0.5` and specify to use absolute bias (`use_absolute_bias=True`, default is False), the bias used to generate the function is either 0.25 or 0.75, both with probability 50%. Generally, if we set `use_absolute_bias=True; absolute_bias=a` for $a \in [0, 1]$, the bias is either $(1 + a)/2$ or $(1 - a)/2$, both with probability 50%.

To display these different modes, we repeatedly generate random Boolean functions under three different constraints (`f` with bias $p = 0.75$, `g` with absolute bias 0.5, and `h` an unbiased function, i.e., with bias $p = 0.5$), and compare the empirical Hamming weight distribution of the three families of functions.

```
n = 4
n_simulations = 10000

counts = np.zeros((3, 2 n + 1), dtype=int)

for _ in range(n_simulations):
    f = bf.random_function(n, bias=0.75)
    g = bf.random_function(n, absolute_bias=0.5, use_absolute_bias=True)
    h = bf.random_function(n, absolute_bias=0.5) #absolute_bias ignored!

    counts[0, f.hamming_weight] += 1
    counts[1, g.hamming_weight] += 1
    counts[2, h.hamming_weight] += 1

labels = ["bias = 0.75", "absolute bias = 0.5", "random (bias = 0.5)"]
x = np.arange(2 n + 1)
width = 0.3

fig, ax = plt.subplots()
for i in range(3):
    ax.bar(x - width + i * width, counts[i] / n_simulations, width=width, label=labels[i])

ax.legend(frameon=False)
ax.set_xticks(x)
ax.set_xlabel("Hamming weight")
```

```
ax.set_ylabel(f"Proportion of {n}-input functions")
plt.show()
```

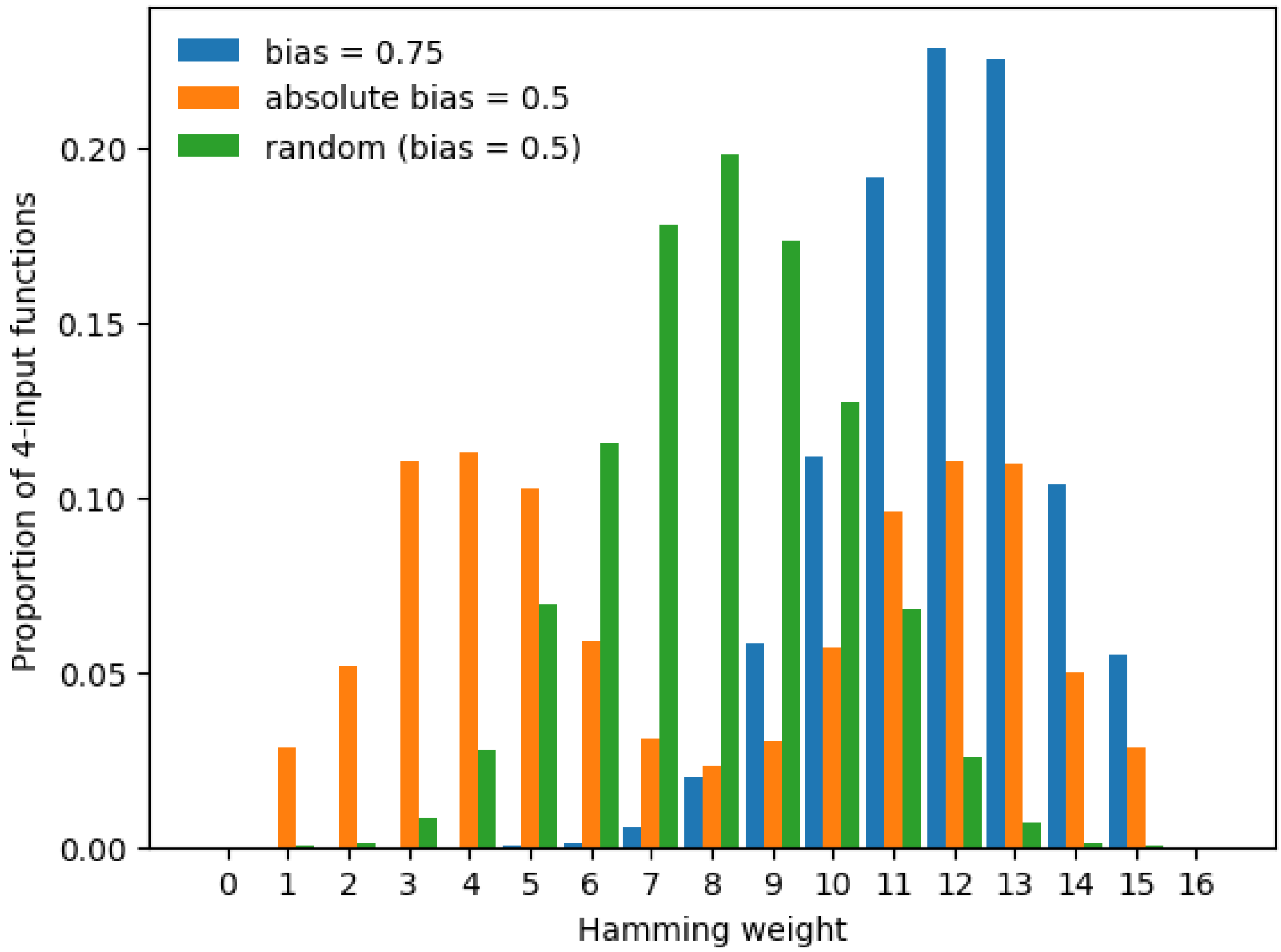

This plot exemplifies the difference between bias and absolute bias:

- Specifying the bias shifts the mode of the Hamming weight distribution to the value of `bias`.
- Specifying the absolute bias yields random functions with a bimodal Hamming weight distribution.

Note that `absolute_bias=0.5` is ignored in the generation of `h`. If the value of `absolute_bias` should be used, this must be specified via `use_absolute_bias=True`. By default, the value of `bias` (default 0.5) is used.

In the above plot, we notice a lack of functions with Hamming weight 0 and $16 = 2^n$. These constant functions are degenerate and thus not generated unless we set `allow_degenerate_functions=True`, which as we see below, slightly modifies the resulting Hamming weight distributions.

```
counts[:] = 0

for _ in range(n_simulations):
    f = bf.random_function(
        n, bias=0.75, allow_degenerate_functions=True
    )
    g = bf.random_function(
        n, absolute_bias=0.5, use_absolute_bias=True, allow_degenerate_functions=True
    )
    h = bf.random_function(
        n, allow_degenerate_functions=True
    )

    counts[0, f.hamming_weight] += 1
```

```
    counts[1, g.hamming_weight] += 1
    counts[2, h.hamming_weight] += 1

fig, ax = plt.subplots()
for i in range(3):
    ax.bar(x - width + i * width, counts[i] / n_simulations, width=width, label=labels[i])

ax.legend(frameon=False)
ax.set_xticks(x)
ax.set_xlabel("Hamming weight")
ax.set_ylabel(f"Proportion of {n}-input functions")
plt.show()
```

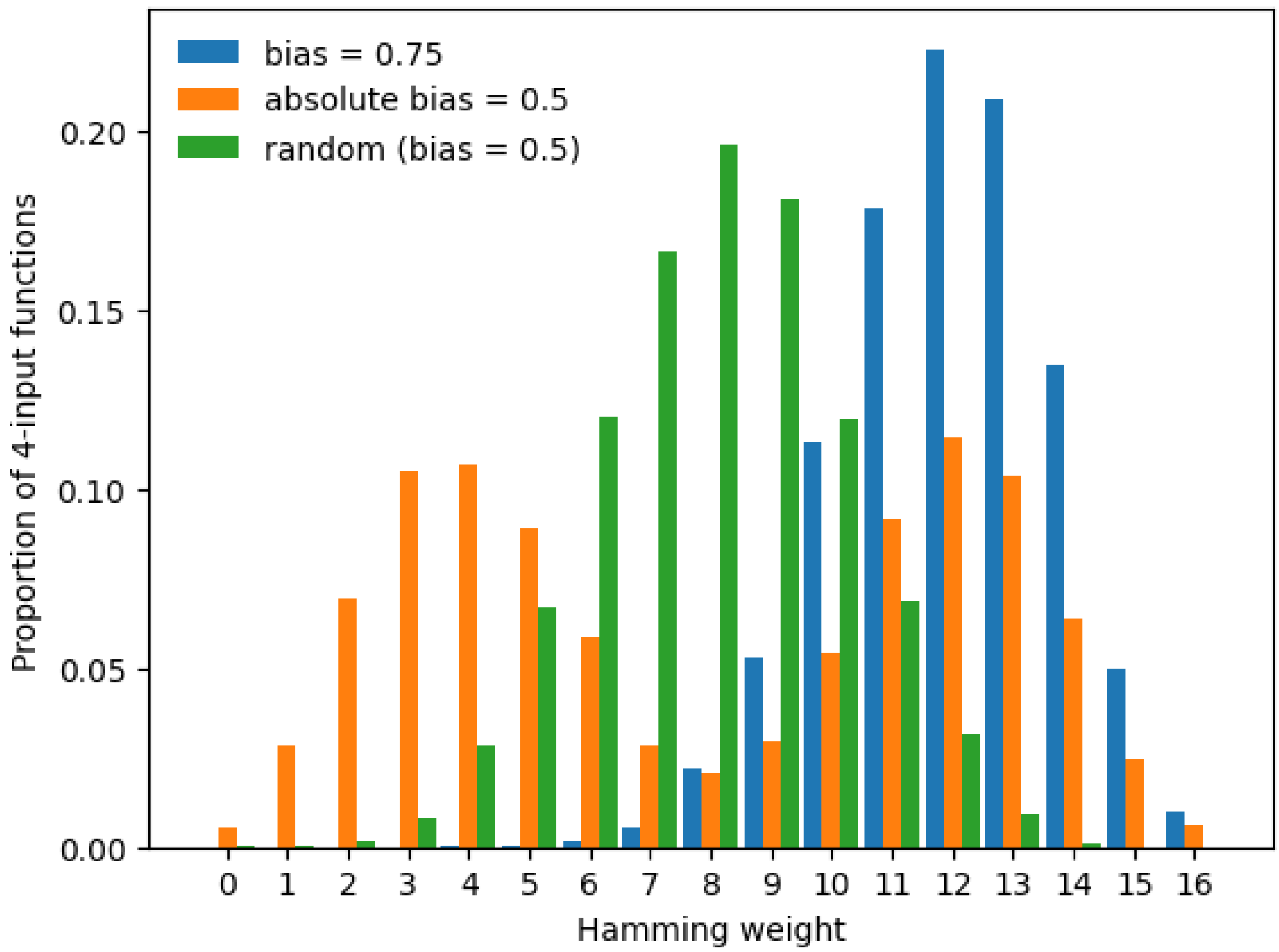

4.9 Summary

This tutorial demonstrated how BoolForge enables uniform random generation of Boolean functions under flexible constraints. Different constraints define fundamentally different ensembles, and being explicit about these choices is essential for a correct generation and interpretation of computational results.

Next steps: The next tutorial exemplifies how these function-level ensembles can be used to uncover new insights into biological regulatory networks, as well as the relationship between network structure and dynamics.

4.10 Common pitfalls

- `absolute_bias` has no effect unless `use_absolute_bias=True`.
- `depth=0` without `exact_depth=True` does not restrict the function space since any Boolean function is at least 0-canalizing.

- Constant functions and other degenerate functions that do not depend on all inputs are only generated if `allow_degenerate_functions=True`. If `layer_structure` or `depth>0` are provided, canalizing functions are generated, which always depend on all inputs. That is, in these cases a possible parameter choice of `allow_degenerate_functions=True` is ignored.
- For larger n (e.g., $n > 5$), set `allow_degenerate_functions=True` to avoid expensive degeneracy tests. Almost all functions in many variables are non-degenerate.

5 Ensemble experiments with random Boolean functions

In this tutorial, we explore how BoolForge's random Boolean function generator can be used to generate large ensembles of Boolean functions with prescribed structural properties, whose statistical and dynamical characteristics can then be studied.

5.1 What you will learn

In this tutorial you will learn how to:

- compute the prevalence of canalization, k-canalization, and nested canalization,
- determine distributions of canalizing strength and normalized input redundancy,
- investigate correlations between absolute bias and canalization,
- generate and analyze dynamically distinct nested canalizing functions.

It is strongly recommended to complete the previous tutorials first.

5.2 Setup

```
import boolforge as bf
import numpy as np
import matplotlib.pyplot as plt
import pandas as pd
import scipy.stats as stats
```

5.3 Prevalence of canalization

Using random sampling, we estimate how frequently Boolean functions of degree n exhibit a given canalizing depth.

```
n_simulations = 1000
ns = np.arange(2, 7)
canalizing_depths = np.arange(max(ns) + 1)

count_depths = np.zeros((len(ns), max(ns) + 1))

for _ in range(n_simulations):
    for i, n in enumerate(ns):
        f = bf.random_function(n)
        count_depths[i, f.get_canalizing_depth()] += 1

count_depths /= n_simulations

fig, ax = plt.subplots()
for i, depth in enumerate(canalizing_depths):
    ax.bar(
        ns,
        count_depths[:, i],
        bottom=np.sum(count_depths[:, :i], axis=1),
        label=str(depth),
    )

ax.legend(
    frameon=False,
    loc="center",
    bbox_to_anchor=(0.5, 1.1),
    ncol=8,
    title="canalizing depth",
```

```
)
ax.set_xticks(ns)
ax.set_xlabel("Number of essential variables")
ax.set_ylabel("Proportion of functions")
plt.show()

out = pd.DataFrame(
    count_depths,
    index=["n=" + str(n) for n in ns],
    columns=["k=" + str(k) for k in canalizing_depths],
)

print(out.to_string())
```

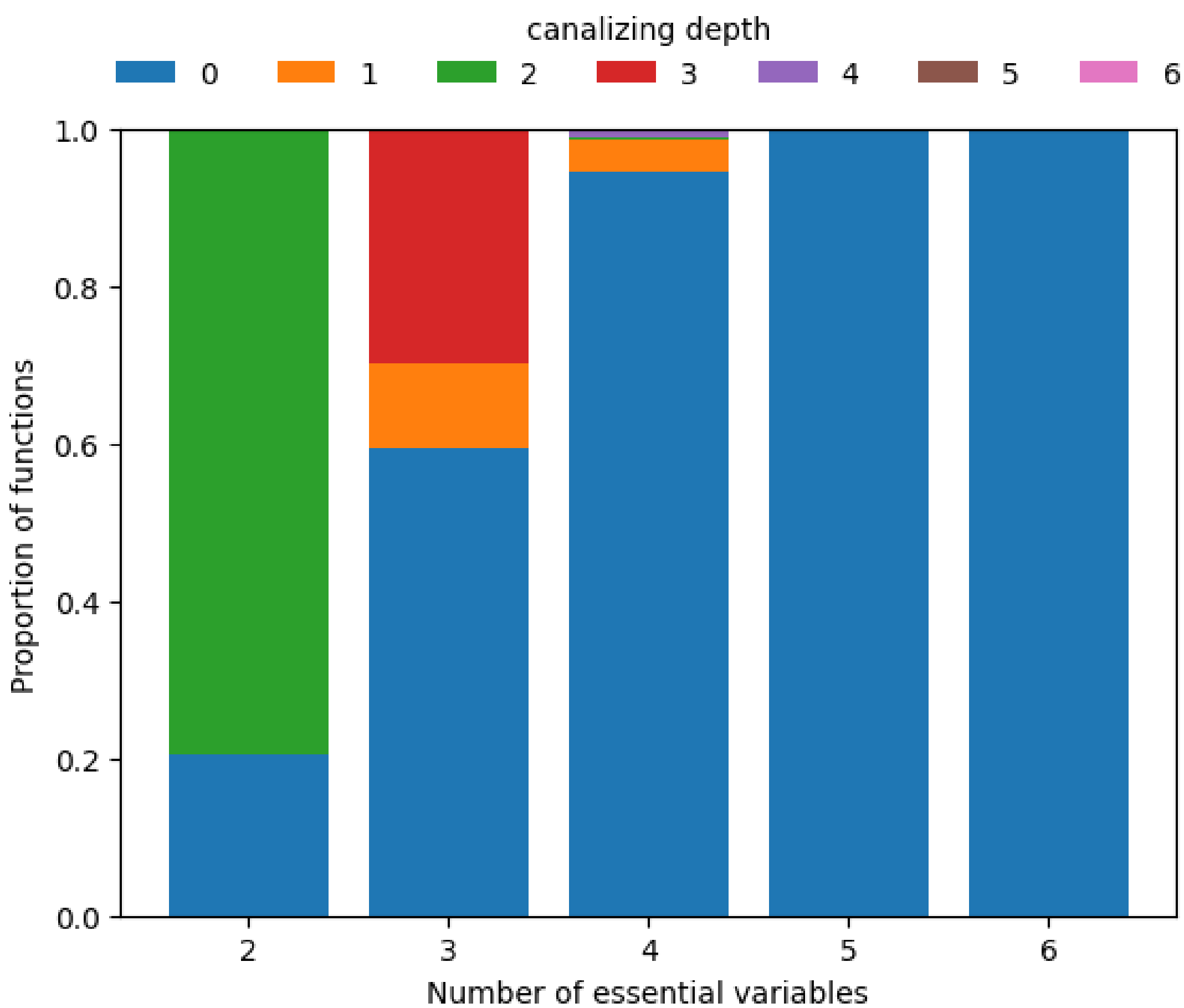


```
       k=0    k=1    k=2    k=3    k=4  k=5  k=6
n=2  0.205  0.000  0.795  0.000  0.000  0.0  0.0
n=3  0.594  0.109  0.000  0.297  0.000  0.0  0.0
n=4  0.945  0.042  0.002  0.000  0.011  0.0  0.0
n=5  1.000  0.000  0.000  0.000  0.000  0.0  0.0
n=6  1.000  0.000  0.000  0.000  0.000  0.0  0.0
```

We see that hardly any Boolean function with $n \geq 5$ inputs is canalizing, let alone nested canalizing. This makes the finding that most Boolean functions in published Boolean gene regulatory network models are nested canalizing very surprising [Kadelka et al., 2024].

5.3.1 Restricting to canalizing functions

To zoom in on the few functions that are canalizing for higher n, we can simply require `depth=1` and repeat the above analysis.

```
count_depths = np.zeros((len(ns), max(ns) + 1))

for _ in range(n_simulations):
    for i, n in enumerate(ns):
        f = bf.random_function(n, depth=1)
        count_depths[i, f.get_canalizing_depth()] += 1

count_depths /= n_simulations

fig, ax = plt.subplots()
for i, depth in enumerate(canalizing_depths):
    ax.bar(
        ns,
        count_depths[:, i],
        bottom=np.sum(count_depths[:, :i], axis=1),
        label=str(depth),
    )

ax.legend(
    frameon=False,
    loc="center",
    bbox_to_anchor=(0.5, 1.1),
    ncol=8,
    title="canalizing depth",
)
ax.set_xticks(ns)
ax.set_xlabel("Number of essential variables")
ax.set_ylabel("Proportion of functions")
plt.show()

out = pd.DataFrame(
    count_depths,
    index=["n=" + str(n) for n in ns],
    columns=["k=" + str(k) for k in canalizing_depths],
);

print(out.to_string())
```

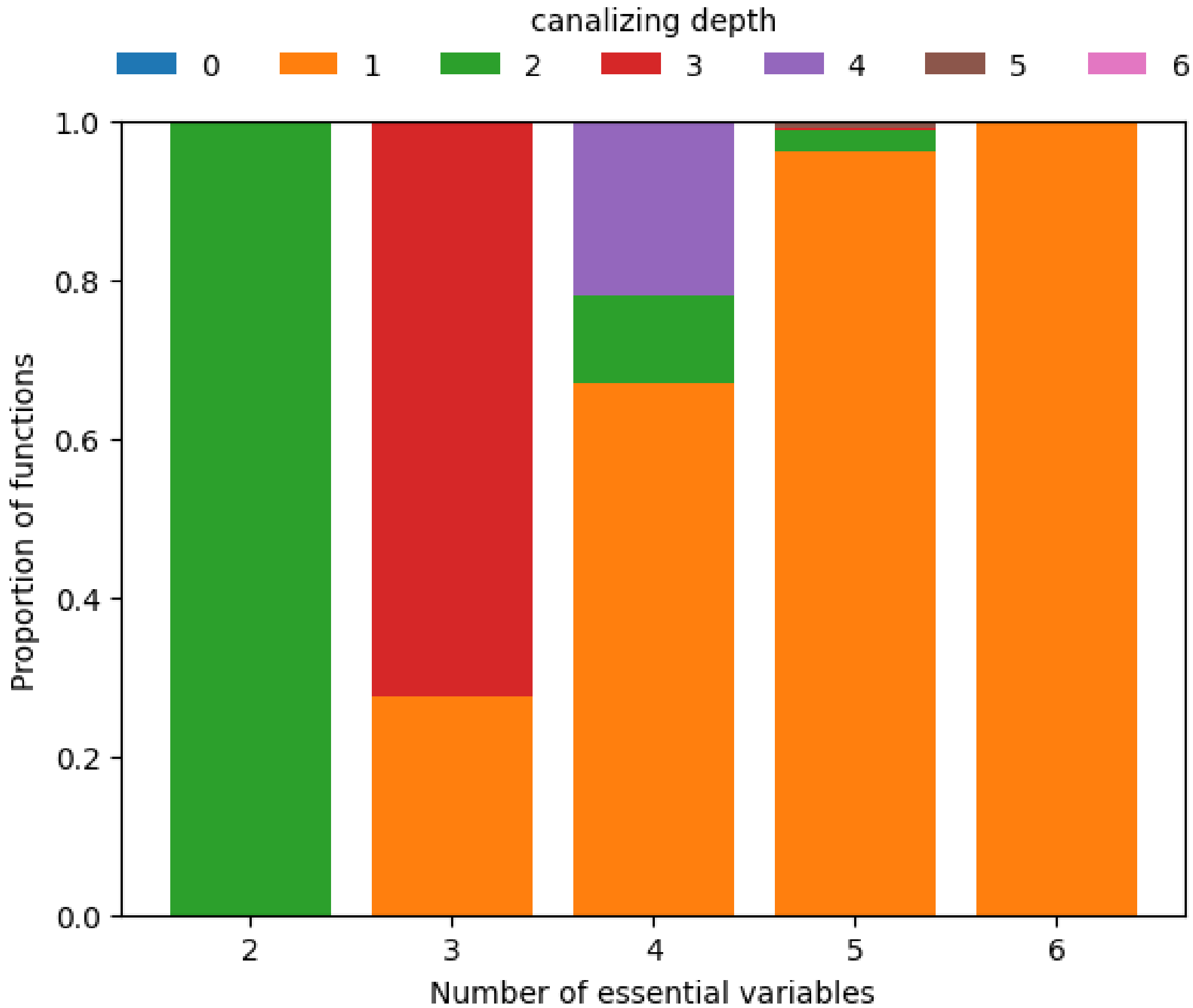


```
     k=0   k=1   k=2   k=3   k=4   k=5  k=6
n=2  0.0  0.000  1.000  0.000  0.00  0.000  0.0
n=3  0.0  0.274  0.000  0.726  0.00  0.000  0.0
n=4  0.0  0.670  0.110  0.000  0.22  0.000  0.0
n=5  0.0  0.963  0.026  0.002  0.00  0.009  0.0
n=6  0.0  0.999  0.001  0.000  0.00  0.000  0.0
```

This analysis reveals that among Boolean functions of degree $n \geq 5$, functions with few conditionally canalizing variables are much more abundant than functions with more conditionally canalizing variables, which is mathematically obvious due to the recursive nature of the definition of k-canalization [He and Macauley, 2016].

5.4 Collective canalization vs degree

Using a similar setup, we can investigate if and how the various measures of collective canalization, specifically canalizing strength [Kadelka et al., 2023] and the normalized input redundancy [Gates et al., 2021], change when the degree of the functions changes.

```
n_simulations = 100
ns = np.arange(2, 8)

canalizing_strengths = np.zeros((len(ns), n_simulations))
input_redundancies = np.zeros((len(ns), n_simulations))

for j in range(n_simulations):
```

```python
    for i, n in enumerate(ns):
        f = bf.random_function(n)
        canalizing_strengths[i, j] = f.get_canalizing_strength()
        input_redundancies[i, j] = f.get_input_redundancy()

width = 0.4
fig, ax = plt.subplots()

ax.violinplot(
    canalizing_strengths.T,
    positions=ns - width / 2,
    widths=width,
    showmeans=True,
    showextrema=False,
)
ax.scatter([], [], color="C0", label="canalizing strength")

ax.violinplot(
    input_redundancies.T,
    positions=ns + width / 2,
    widths=width,
    showmeans=True,
    showextrema=False,
)
ax.scatter([], [], color="C1", label="normalized input redundancy")

ax.legend(
    loc="center",
    bbox_to_anchor=(0.5, 1.05),
    frameon=False,
    ncol=2,
)
ax.set_xlabel("Number of essential variables")
ax.set_ylabel("Value")
plt.show()
```

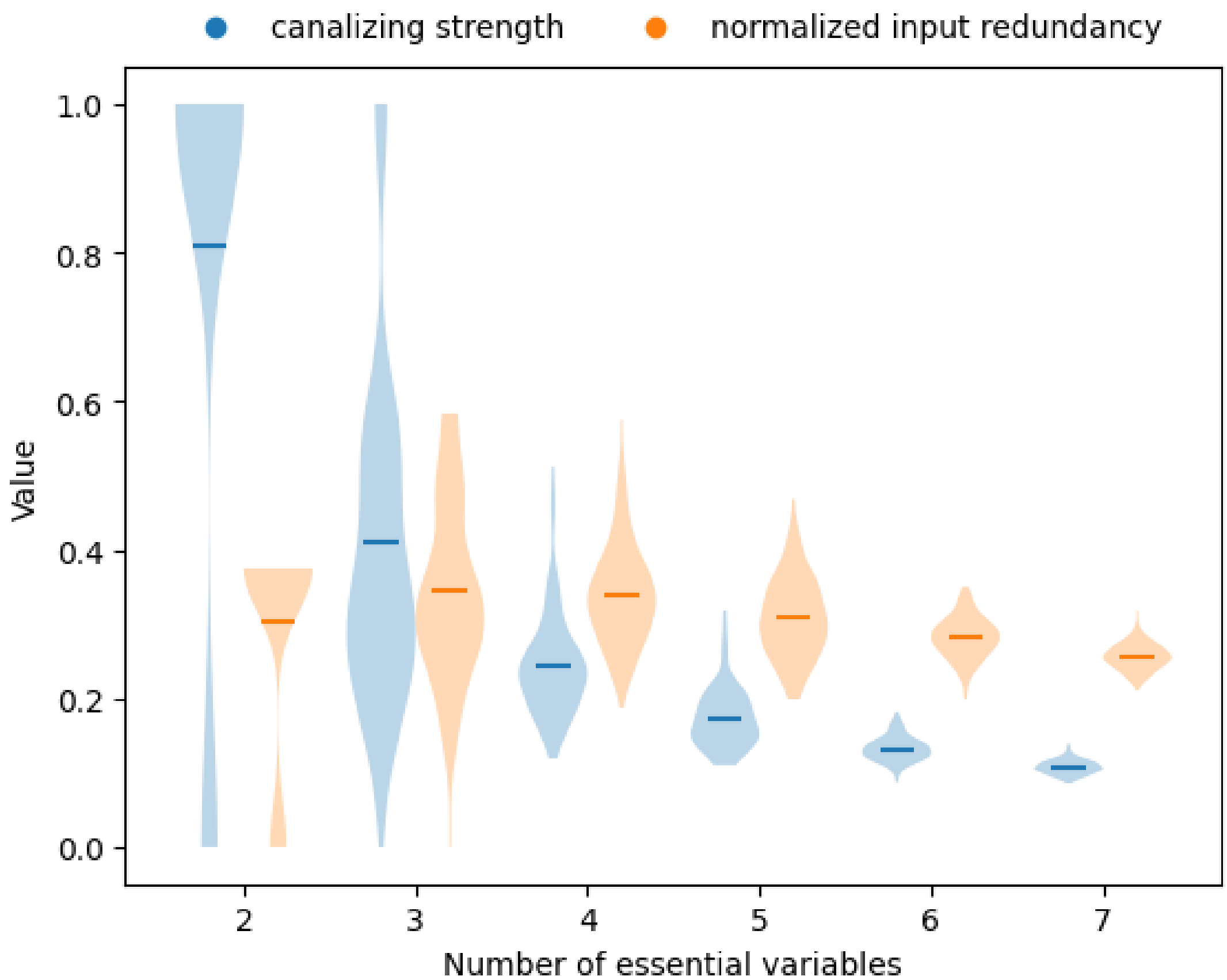

Both measures decrease with increasing degree, but canalizing strength declines more sharply.

5.4.1 Stratification by canalizing depth

If we stratify this analysis by canalizing depth (exact canalizing depth using `exact_depth=True` or minimal canalizing depth using the default `exact_depth=False`), we can confirm that functions with more conditionally canalizing variables tend to also have higher average collective canalization, irrespective of how it is measured. In other words, the various measures of canalization are all highly correlated.

```
n_simulations = 100
exact_depth = False
ns = np.arange(2, 7)

max_depth = max(ns)

canalizing_strengths = np.zeros((len(ns), max_depth + 1, n_simulations))
input_redundancies = np.zeros((len(ns), max_depth + 1, n_simulations))

for k in range(n_simulations):
    for i, n in enumerate(ns):
        for depth in np.append(np.arange(n - 1), n):
            f = bf.random_function(n, depth=depth, exact_depth=exact_depth)
            canalizing_strengths[i, depth, k] = f.get_canalizing_strength()
            input_redundancies[i, depth, k] = f.get_input_redundancy()

fig, ax = plt.subplots(2, 1, figsize=(6, 6), sharex=True)

base_gap = 1.0
```

```
intra_gap = 0.3
width = 0.28

for ii, (data, label) in enumerate(
    zip(
        [canalizing_strengths, input_redundancies],
        ["canalizing strength", "normalized input redundancy"],
    )
):
    positions = []
    values = []
    colors = []
    group_centers = []

    current_x = 0.0
    for i, n in enumerate(ns):
        depths = np.append(np.arange(n - 1), n)
        offsets = np.linspace(
            -(len(depths) - 1) * intra_gap / 2,
            (len(depths) - 1) * intra_gap / 2,
            len(depths),
        )
        group_positions = current_x + offsets
        positions.extend(group_positions)
        group_centers.append(current_x)

        for d in depths:
            values.append(data[i, d, :])
            colors.append("C" + str(d))

        group_width = (len(depths) - 1) * intra_gap
        current_x += group_width / 2 + base_gap + width + intra_gap

    for pos, val, c in zip(positions, values, colors):
        vp = ax[ii].violinplot(val, positions=[pos], widths=width, showmeans=True, showextrema=
    False)
        for body in vp["bodies"]:
            body.set_facecolor(c)
            body.set_alpha(0.85)
        vp["cmeans"].set_color("k")

    ax[ii].set_ylabel(label)
    ax[ii].set_ylim([-0.02, 1.02])

ax[1].set_xlabel("Number of essential variables (n)")
ax[1].set_xticks(group_centers)
ax[1].set_xticklabels(ns)

depth_handles = [
    plt.Line2D([0], [0], color="C" + str(d), lw=5, label=str(d))
    for d in range(max_depth + 1)
]

fig.legend(
    handles=depth_handles,
    loc="upper center",
    ncol=7,
    frameon=False,
    title="exact canalizing depth" if exact_depth else "minimal canalizing depth",
```

```
)
plt.show()
```

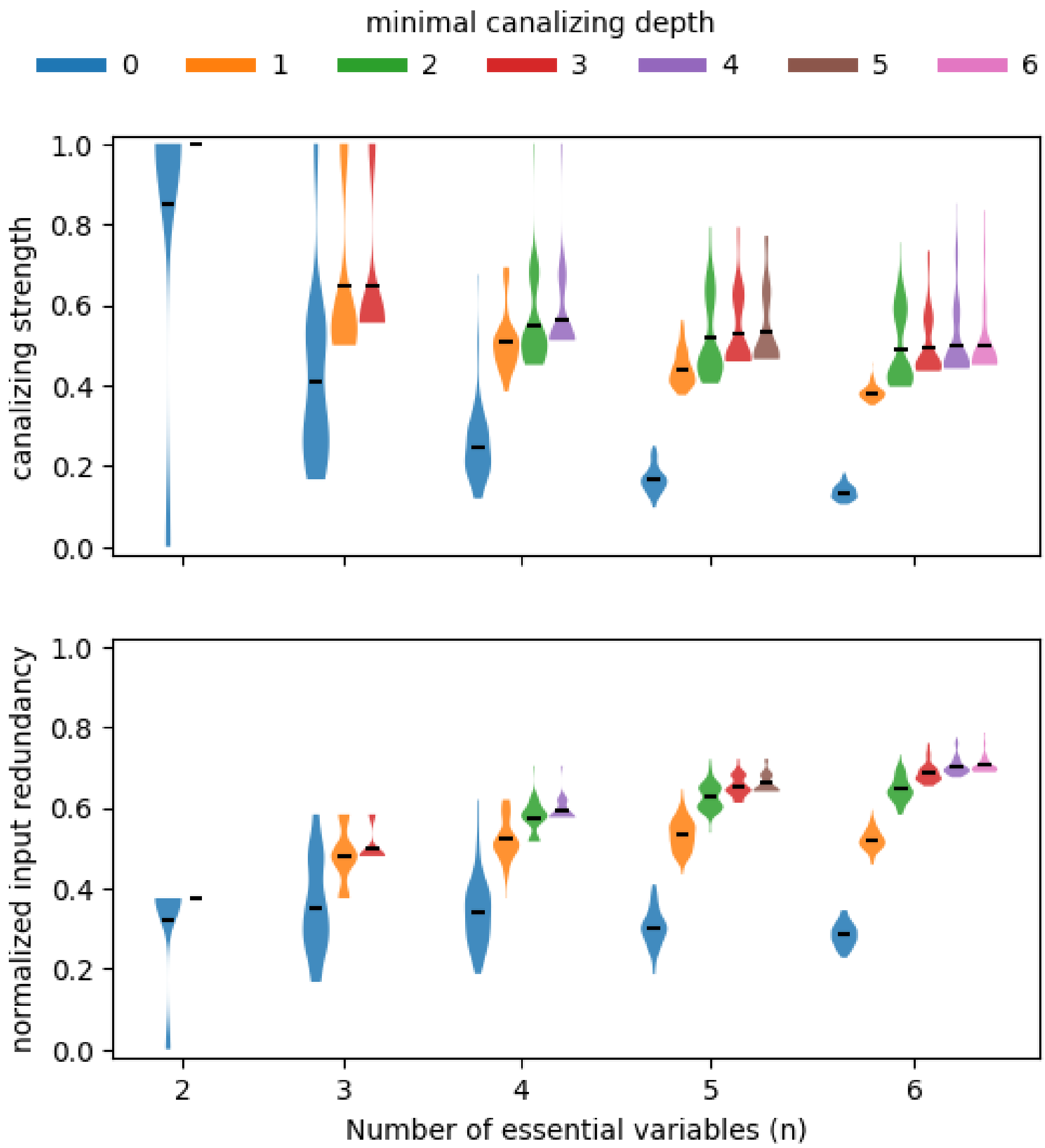

5.4.2 Correlation between canalizing strength and input redundancy

We can generate all (non-degenerate) Boolean functions of a certain degree n (only feasible up to $n = 4$) and compare canalizing strength and input redundancy.

```
n = 3
allow_degenerate_functions = False
degenerate = np.zeros(2   (2 n), dtype=bool)
strengths = np.zeros(2   (2 n))
redundancies = np.zeros(2   (2 n))

for i, fvec in enumerate(bf.get_left_side_of_truth_table(2 n)):
    f = bf.BooleanFunction(fvec)
    strengths[i] = f.get_canalizing_strength()
    redundancies[i] = f.get_input_redundancy()
    if not allow_degenerate_functions:
```

```
        degenerate[i] = f.is_degenerate()

if allow_degenerate_functions:
    which = np.ones(2   (2 n), dtype=bool)
else:
    which = ~degenerate


plt.figure(figsize=(5, 4))
plt.scatter(strengths[which], redundancies[which], alpha=0.7)
plt.xlabel("Canalizing strength")
plt.ylabel("Normalized input redundancy")
plt.tight_layout()
plt.show()

stats.spearmanr(strengths[which], redundancies[which])
```

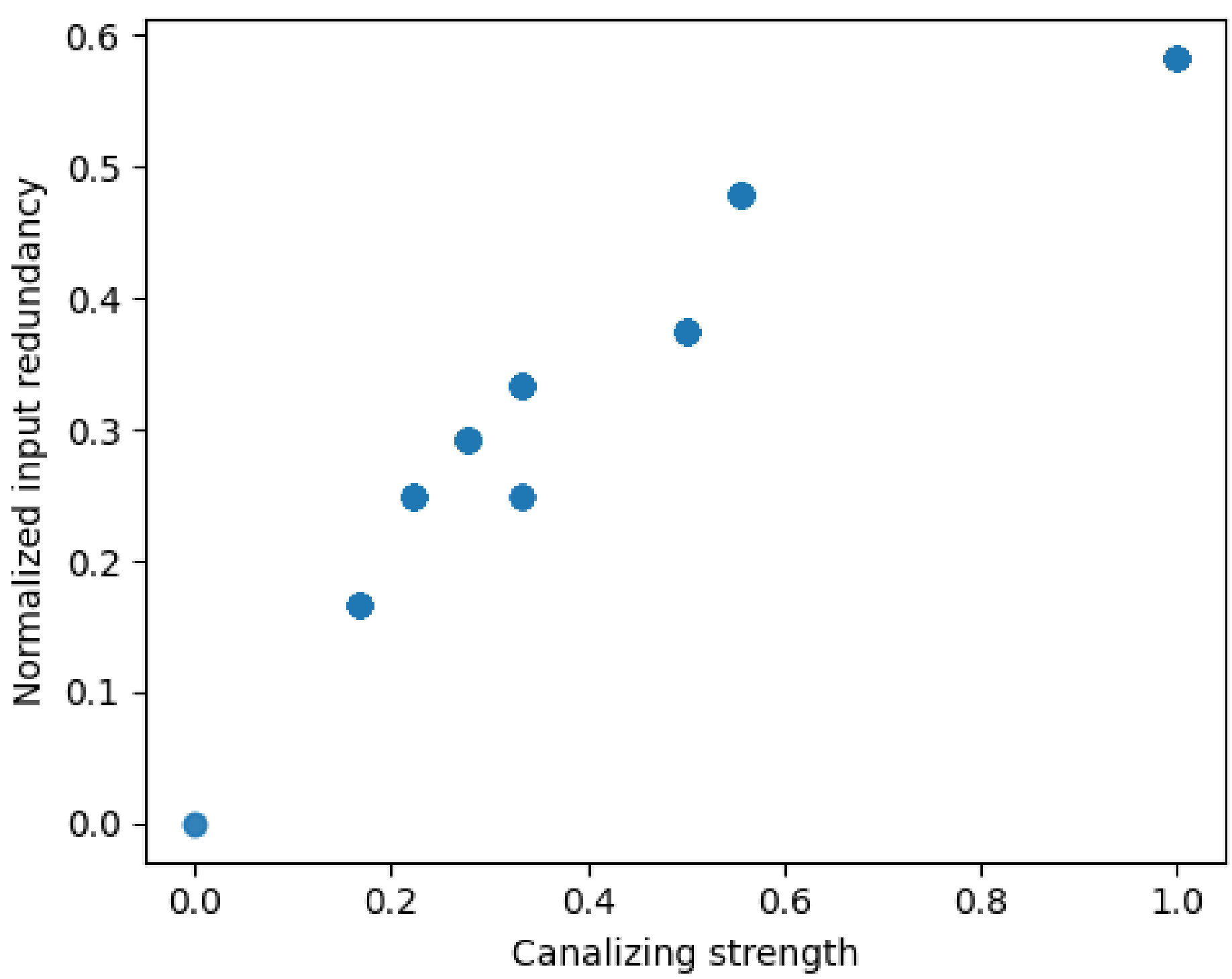


```
SignificanceResult(statistic=np.float64(0.9700304760700043), pvalue=np.float64(1.0759731607818433e
    -134))
```

Both measures are highly correlated but markedly not the same, which becomes even more evident when rerunning the analysis for $n = 4$ (see Kadelka [2026]). Some functions possess relatively high canalizing strength but low input redundancy, and vice versa. It remains an open question what drives this behavior.

5.5 Correlation between canalization and bias

All metrics used to assess the sensitivity of Boolean functions (canalization, absolute bias, average sensitivity) are correlated. For example, functions with higher absolute bias are more likely to be canalizing.

```
ns = np.arange(2, 6)
n_simulations = 3000
```

```
bias_values = np.linspace(0, 1, 21)

count_canalizing = np.zeros((len(ns), len(bias_values)), dtype=int)

for i, n in enumerate(ns):
    for _ in range(n_simulations):
        for j, bias in enumerate(bias_values):
            f = bf.random_function(n, bias=bias, allow_degenerate_functions=True)
            if f.is_canalizing():
                count_canalizing[i, j] += 1

fig, ax = plt.subplots()
for i, n in enumerate(ns):
    ax.plot(bias_values, count_canalizing[i] / n_simulations, label=f"n={n}")

xticks = [0, 0.25, 0.5, 0.75, 1]
ax.set_xticks(xticks)
ax.set_xticklabels([f"{p} ({round(200*abs(p-0.5))}%)" for p in xticks])
ax.set_xlabel("bias (absolute bias)")
ax.set_ylabel("probability canalizing")
ax.legend(
    loc="center",
    frameon=False,
    bbox_to_anchor=(0.5, 1.05),
    ncol=6,
)
plt.show()
```

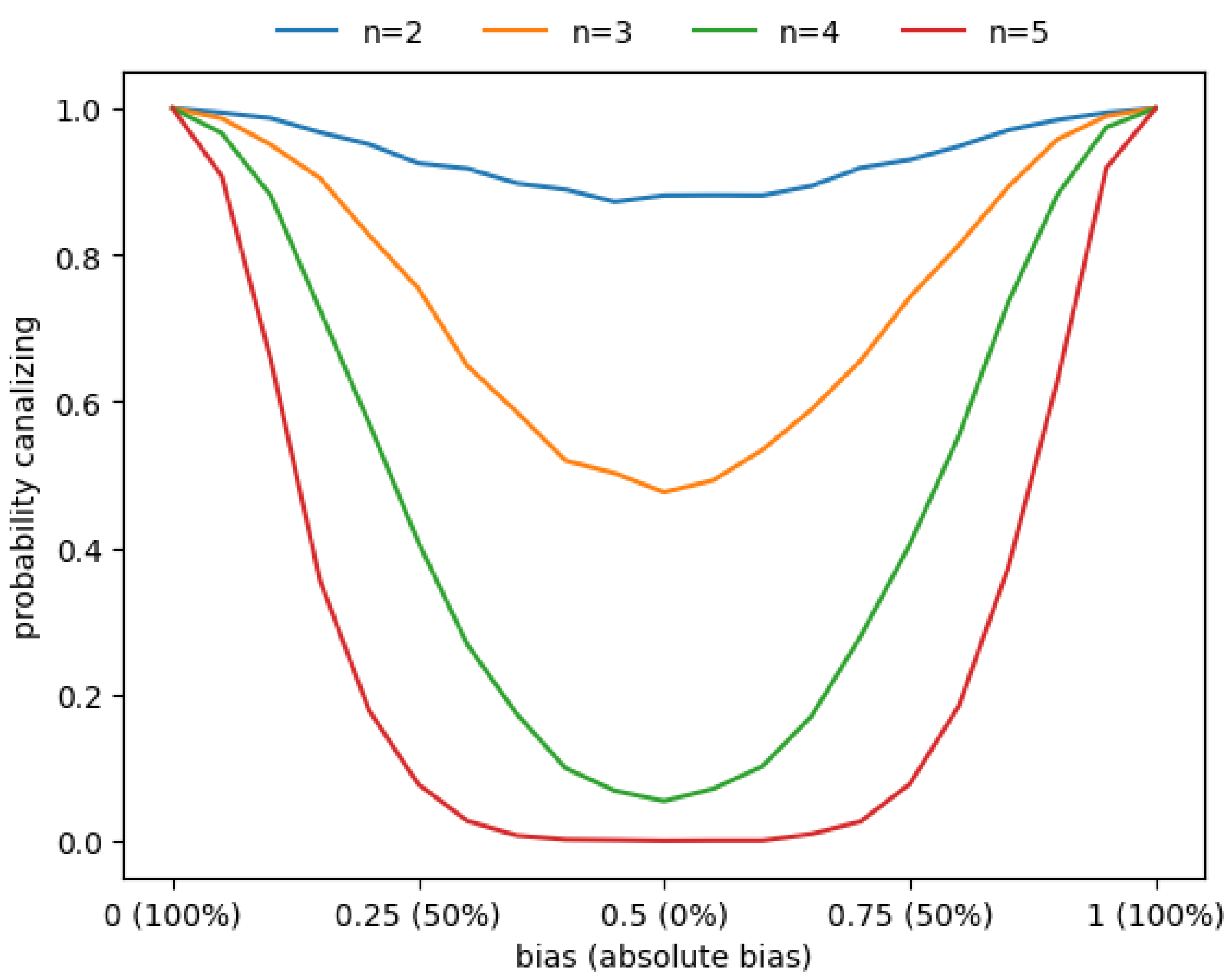

5.5.1 Degeneracy vs bias

Similarly, the probability that a function is degenerate (i.e., that it does not depend on all its variables) also increases as the absolute bias increases.

```
count_degenerate = np.zeros((len(ns), len(bias_values)), dtype=int)

for i, n in enumerate(ns):
    for _ in range(n_simulations):
        for j, bias in enumerate(bias_values):
            f = bf.random_function(n, bias=bias, allow_degenerate_functions=True)
            if f.is_degenerate():
                count_degenerate[i, j] += 1

fig, ax = plt.subplots()
for i, n in enumerate(ns):
    ax.plot(bias_values, count_degenerate[i] / n_simulations, label=f"n={n}")

ax.set_xticks(xticks)
ax.set_xticklabels([f"{p} ({round(200*abs(p-0.5))}%)" for p in xticks])
ax.set_xlabel("bias (absolute bias)")
ax.set_ylabel("probability degenerate")
ax.legend(
    loc="center",
    frameon=False,
    bbox_to_anchor=(0.5, 1.05),
    ncol=6,
)
plt.show()
```

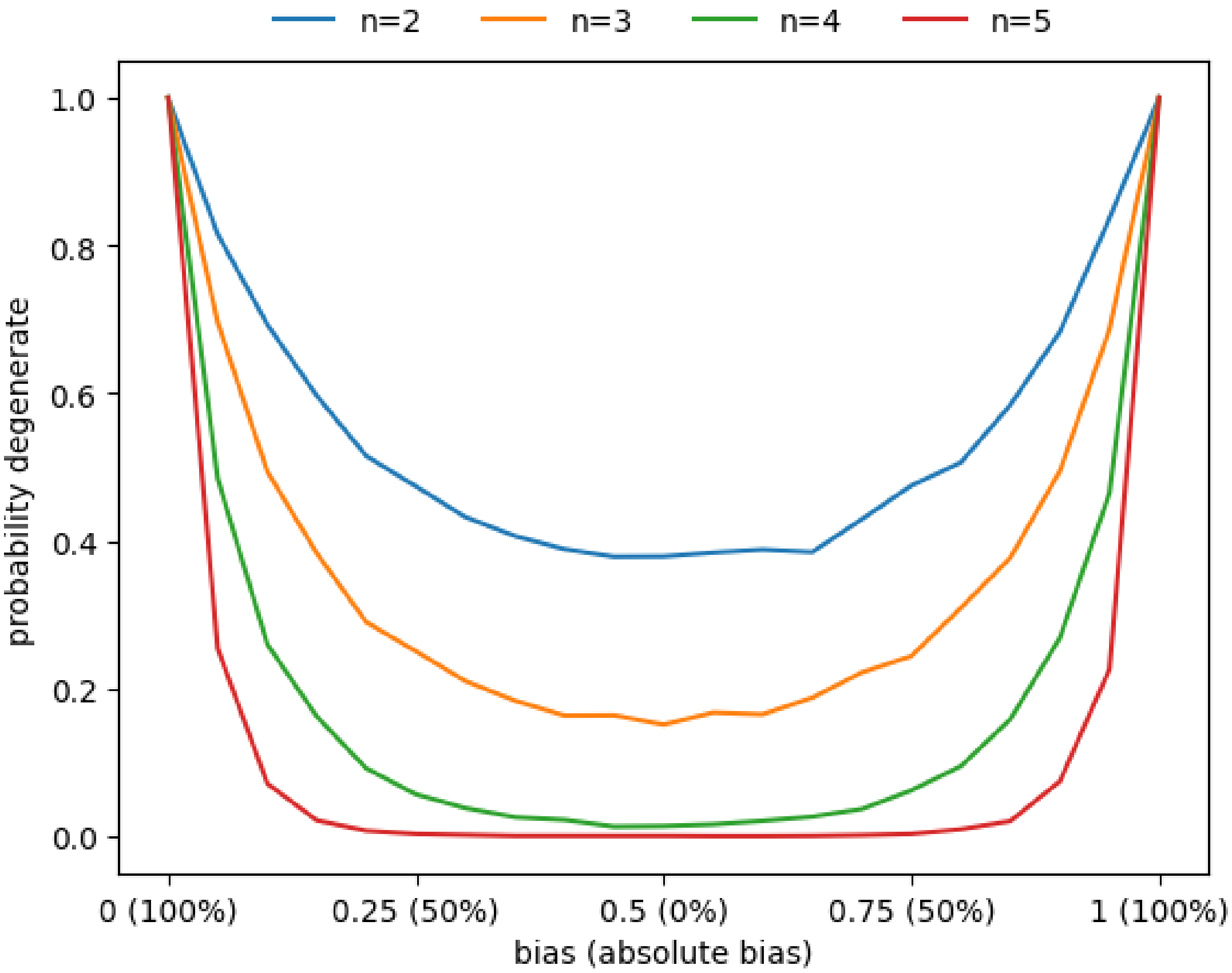

5.6 Analyzing functions with specific canalizing layer structure

The average sensitivity of the Boolean functions governing the updates in a Boolean network, determines the stability of the network to perturbations. More generally, it determines the dynamical regime of the network (see Tutorial 8). The ability to generate canalizing functions with a specific canalizing layer structure enables us to investigate the link between layer structure and average sensitivity, as well as other properties, such as canalizing strength or effective degree.

For nested canalizing functions of a given degree n, there exists a bijection between their absolute bias and their canalizing layer structure [Kadelka et al., 2017]. The function `boolforge.hamming_weight_to_ncf_layer_structure(degree` implements this. NCFs with the same layer structure have the same dynamical properties. That is, they have the same average sensitivity, canalizing strength and the same effective degree. Iterating over all possible absolute biases (parametrized by the possible Hamming weights), we can thus generate all dynamically different types of n-input NCFs and investigate their average sensitivity, which we can compute exactly for relatively low degree.

```
n = 5
all_hamming = np.arange(1, 2   (n - 1), 2)
all_abs_bias = 2 * np.abs(all_hamming/2 n - 0.5)

avg_sens = np.zeros(2   (n - 2))
can_strength = np.zeros_like(avg_sens)
eff_degree = np.zeros_like(avg_sens)
layer_structures = []

for i, w in enumerate(all_hamming):
    layer = bf.hamming_weight_to_ncf_layer_structure(n, w)
```

```
    layer_structures.append(layer)
    f = bf.random_function(n, layer_structure=layer)
    avg_sens[i] = f.get_average_sensitivity(exact=True, normalized=False)
    can_strength[i] = f.get_canalizing_strength()
    eff_degree[i] = f.get_effective_degree()

df = pd.DataFrame(
    {
        "Hamming weight": all_hamming,
        "Absolute bias": all_abs_bias,
        "Layer structure": list(map(str, layer_structures)),
        "Average sensitivity": avg_sens,
        "Canalizing strength": np.round(can_strength, 4),
        "Effective degree": np.round(eff_degree, 4),
    }
)

print(df.to_string())
```

```
   Hamming weight  Absolute bias Layer structure  Average sensitivity  Canalizing strength
    Effective degree
0               1         0.9375             [5]               0.3125               1.0000
       1.1250
1               3         0.8125          [3, 2]               0.6875               0.7705
       1.3984
2               5         0.6875       [2, 1, 2]               0.9375               0.6369
       1.5938
3               7         0.5625          [2, 3]               1.0625               0.5993
       1.5833
4               9         0.4375       [1, 1, 3]               1.1875               0.5033
       1.7266
5              11         0.3125    [1, 1, 1, 2]               1.3125               0.4657
       1.8021
6              13         0.1875       [1, 2, 2]               1.3125               0.4657
       1.7708
7              15         0.0625          [1, 4]               1.1875               0.5033
       1.6094
```

We notice that nested canalizing functions with higher absolute bias tend to be more sensitive to input changes and also less canalizing. However, the relationship between absolute bias and these other metrics is far from monotonic. Further, we notice that there is a perfect correlation between the average sensitivity of a nested canalizing function and its canalizing strength, and a near perfect correlation between average sensitivity and effective degree.

To investigate the non-monotonic behavior further, we can vary the degree and create line plots that reveal a clear pattern, as shown in Kadelka et al. [2017].

```
ns = np.arange(5, 9)
fig, ax = plt.subplots()

for n in ns:
    all_hamming_weights = np.arange(1, 2   (n - 1), 2)
    all_abs_bias = 2 * np.abs(all_hamming_weights/2 n - 0.5)
    avg_sens = np.zeros(2   (n - 2))

    for i, w in enumerate(all_hamming_weights):
        layer = bf.hamming_weight_to_ncf_layer_structure(n, w)
        f = bf.random_function(n, layer_structure=layer)
```

```
        avg_sens[i] = f.get_average_sensitivity(exact=True, normalized=False)

    ax.plot(all_abs_bias, avg_sens, "x--", label=f"n={n}")

ax.legend(frameon=False)
ax.set_xlabel("Absolute bias")
ax.set_ylabel("Average sensitivity")
plt.show()
```

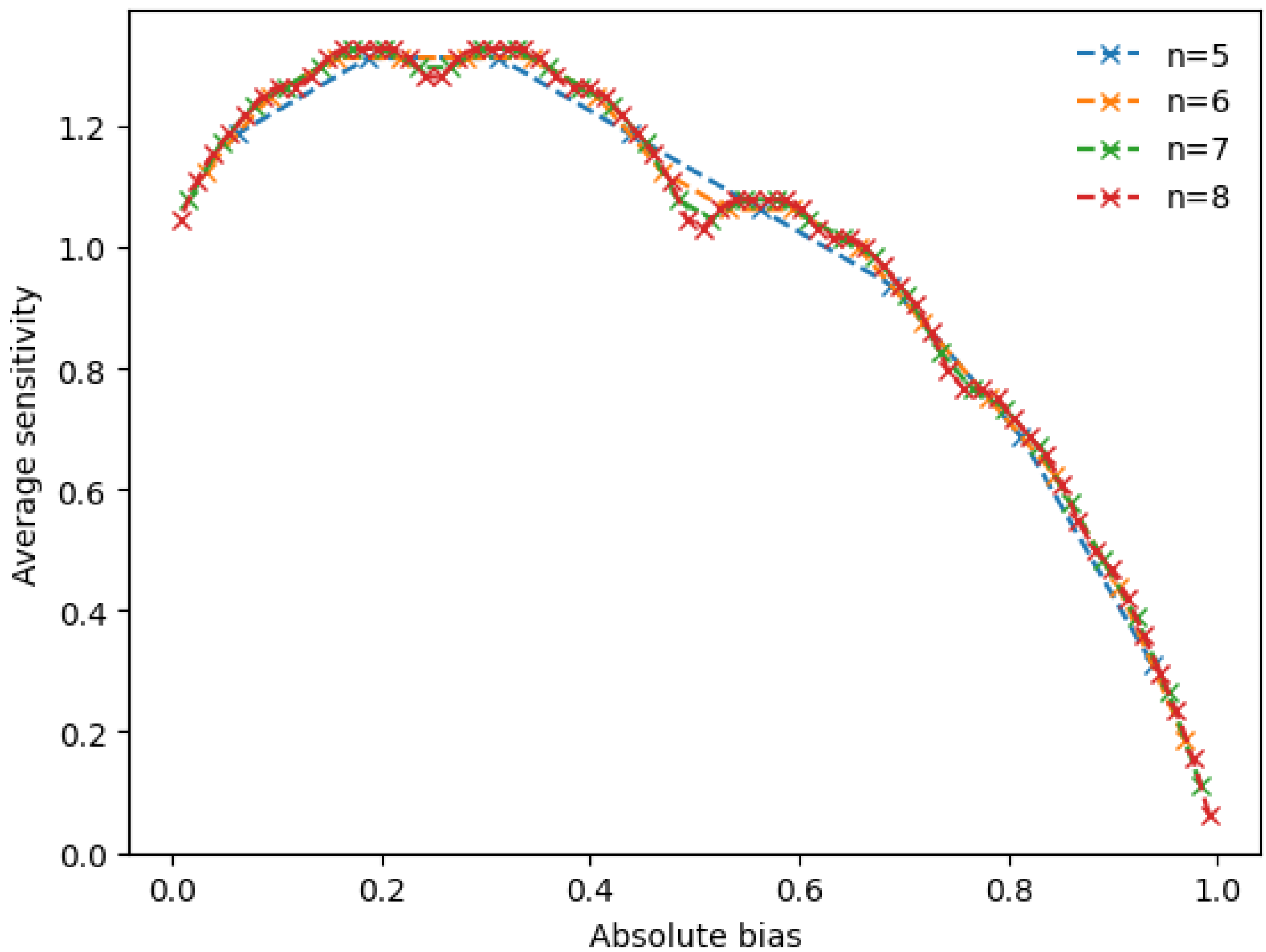

5.7 Summary

This tutorial illustrated how ensembles of Boolean functions generated under explicit constraints reveal systematic relationships between canalization, bias, redundancy, and sensitivity.

The key findings include:

1. Canalization is rare in random functions but common in biology.
2. Canalizing strength and input redundancy both decrease with degree.
3. Functions with high absolute bias are more likely to be highly canalizing.
4. For NCFs, layer structure is uniquely determined by bias.
5. Average sensitivity varies systematically with layer structure.

These relationships constrain the space of biologically plausible functions and suggest evolutionary optimization for robustness.

We now move on to Boolean networks, where Boolean functions serve as node update rules and give rise to collective dynamical behavior.

6 Working with Boolean Networks

While previous tutorials focused on individual Boolean functions, this tutorial introduces Boolean networks, which combine multiple Boolean functions into a dynamical system.

6.1 What you will learn

In this tutorial you will learn how to:

- create Boolean networks,
- compute basic properties of the wiring diagram,
- compute basic properties of Boolean networks.
- transform Boolean networks through structural manipulations such as fixing node values or removing regulatory interactions.

6.2 Setup

```
import boolforge as bf
import numpy as np
```

6.3 Boolean network theory

A Boolean network $F = (f_1, \ldots, f_N)$ is a dynamical system consisting of N Boolean update functions. Each node can be in one of two states, 0 or 1, often interpreted as OFF/ON in biological contexts.

Under *synchronous updating*, all nodes update simultaneously, yielding a deterministic state transition graph on $\{0,1\}^N$. Under *asynchronous updating*, only one node is updated at a time, yielding a stochastic transition graph. BoolForge implements both schemes.

Real biological networks are typically sparsely connected. The *in-degree* of a node is the number of essential inputs of its update function. The *wiring diagram* encodes which nodes regulate which others.

Despite their simplicity, Boolean networks can:

- reproduce complex dynamics (oscillations, multistability),
- predict gene knockout effects,
- identify control strategies,
- scale to genome-wide networks (1000s of nodes).

6.4 Wiring diagrams

We first construct wiring diagrams, which encode network structure independently of specific Boolean functions. Separating topology (encoded in BoolForge by `I`) from dynamics (`F`) allows:

- studying structural properties independent of specific Boolean rules,
- swapping different rule sets on the same topology,
- efficient storage (sparse I, local F vs dense full truth table).

```

# Wiring diagram of a 3-node network
I = [
    [1],
    [0, 2],
    [1],
]

W = bf.WiringDiagram(I=I)

```

```
print("W.N:", W.N)
print("W.variables:", W.variables)
print("W.indegrees:", W.indegrees)
print("W.outdegrees:", W.outdegrees)

fig = W.plot(show=False);
```

```
W.N: 3
W.variables: ['x0' 'x1' 'x2']
W.indegrees: [1 2 1]
W.outdegrees: [1 2 1]
```

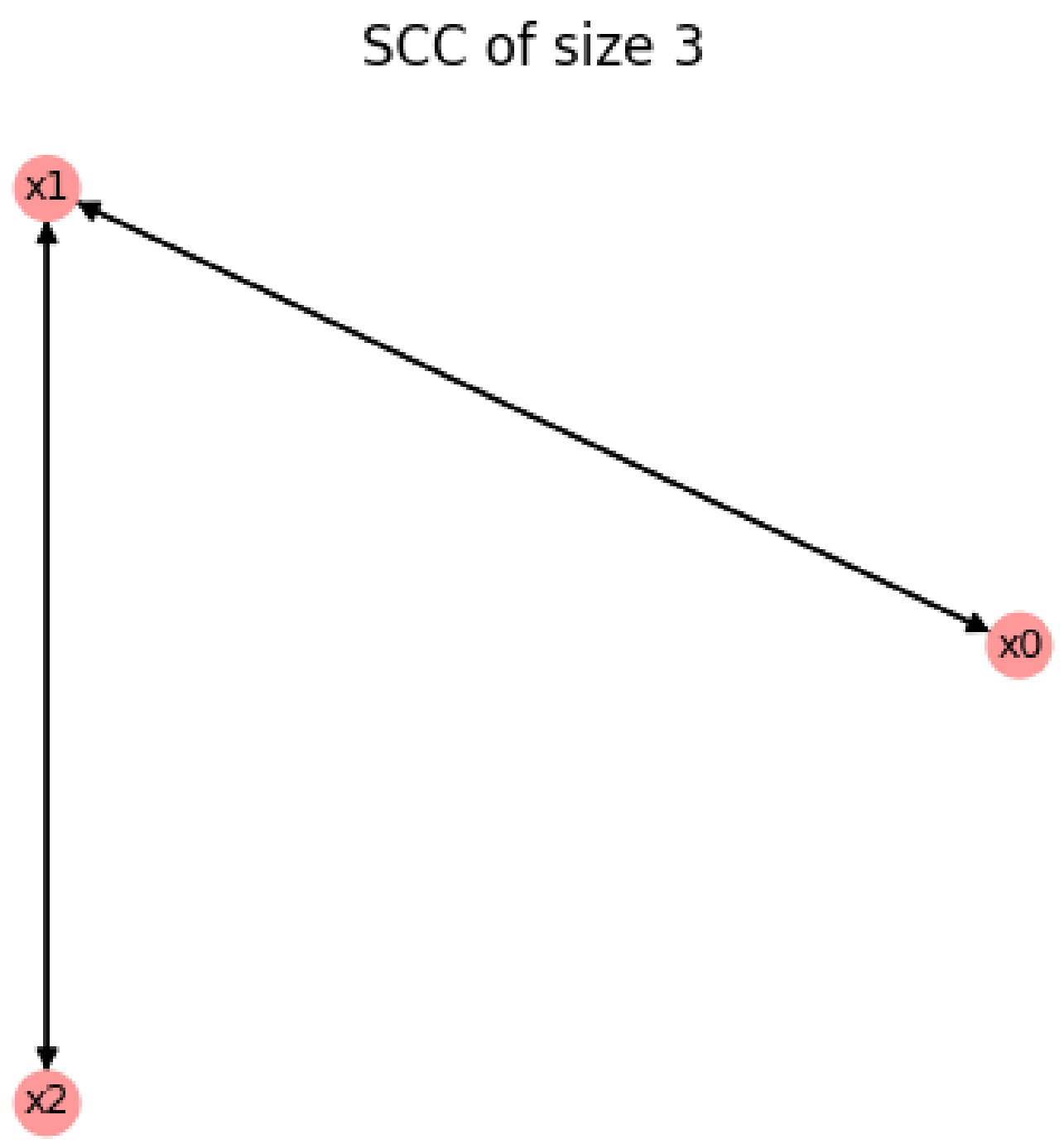

The wiring diagram above consists of $N = 3$ variables, and uses default variable names $x_0, \ldots, x_{N-1}$. The vectors `indegrees` and `outdegrees` describe the number of incoming and outgoing edges for each node.

6.4.1 Example with constants and unequal degrees

The next wiring diagram contains a constant node (source) x_0 and a node that is only regulated but does not regulate any nodes (sink) x_2.

```
I = [
    [],
    [0],
    [0, 1],
]

W = bf.WiringDiagram(I=I)

print("W.N:", W.N)
print("W.variables:", W.variables)
print("W.indegrees:", W.indegrees)
print("W.outdegrees:", W.outdegrees)
```

```
fig = W.plot(show=False)
```

```
W.N: 3
W.variables: ['x0' 'x1' 'x2']
W.indegrees: [0 1 2]
W.outdegrees: [2 1 0]
```

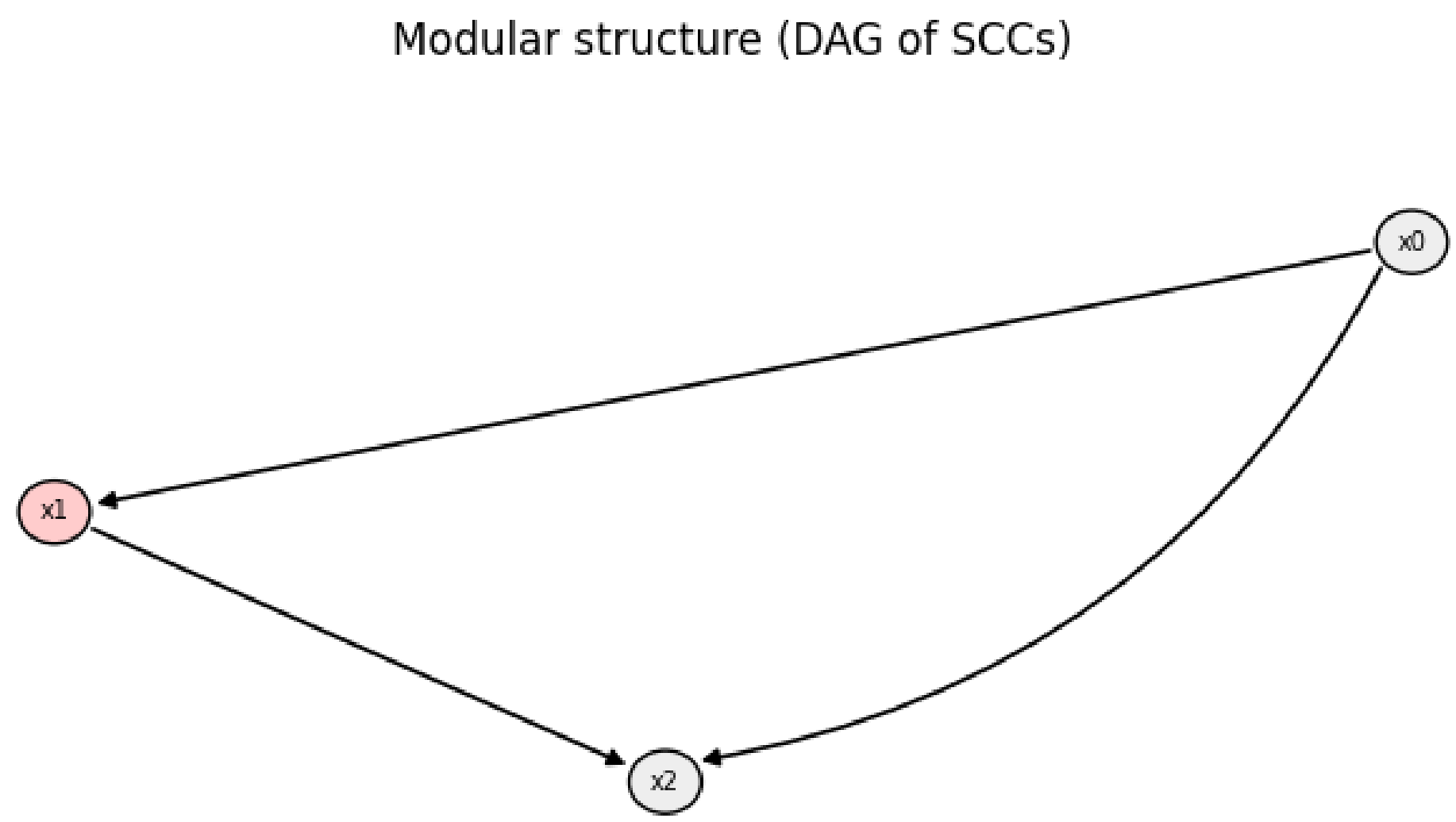

This wiring diagram encodes a *feed-forward loop*, one of the most common *network motifs* in transcriptional networks. It can:

- filter transient signals (coherent FFL with AND gate),
- accelerate response (incoherent FFL),

See Mangan and Alon [2003] for a detailed analysis. `BoolForge` enables the identification of all feed-forward loops:

```
print("W.get_ffls()", W.get_ffls())
```

```
W.get_ffls() {'FFLs': [[0, 1, 2]]}
```

This tells us that `W` contains one FFL, in which x_0 regulates both x_1 and x_2, while x_2 is also regulated by x_1.

`BoolForge` can also identify all feedback loops. For this, we consider another wiring diagram:

```
I2 = [
    [2,1],
    [0],
    [1],
]

W2 = bf.WiringDiagram(I=I2)
fig = W2.plot(show=False)
fig

print("W2.get_fbls()", W2.get_fbls())
```

```
W2.get_fbls() {'FBLs': [[0, 1, 2], [0, 1]]}
```

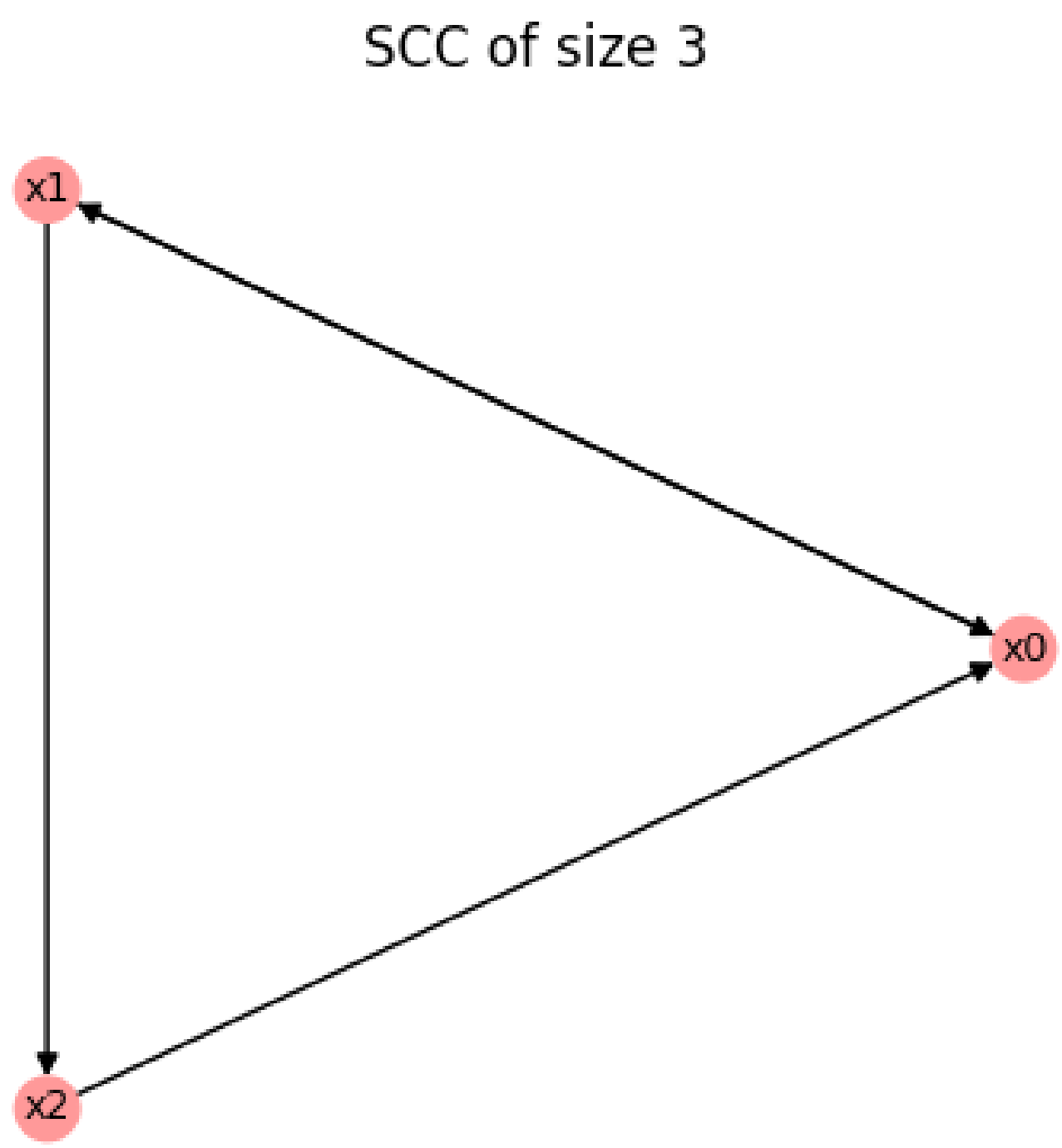

The function `.get_fbls()` identifies all simple cycles in the wiring diagram. In this case, there exists a 2-cycle $x_0 \leftrightarrow x_1$ and a 3-cycle $x_0 \to x_1 \to x_2 \to x_0$.

6.5 Creating Boolean networks

To create a Boolean network, we must specify:

1. A wiring diagram `I`, describing who regulates whom.
2. A list `F` of Boolean update functions (or truth tables), one per node.

```
I = [
    [1],
    [0, 2],
    [1],
]

F = [
    [0, 1],
    [0, 1, 1, 1],
    [0, 1],
]

bn = bf.BooleanNetwork(F=F, I=I)

print(bn.to_truth_table().to_string())
```

```
   x0(t)  x1(t)  x2(t)  x0(t+1)  x1(t+1)  x2(t+1)
0      0      0      0        0        0        0
1      0      0      1        0        1        0
```

```
2       0      1      0       1       0       1
3       0      1      1       1       1       1
4       1      0      0       0       1       0
5       1      0      1       0       1       0
6       1      1      0       1       1       1
7       1      1      1       1       1       1
```

The full truth table of a Boolean network has size $N \times 2^N$ and therefore grows exponentially with the number of nodes.
In practice, however, `BoolForge` never stores this object explicitly. Instead, a Boolean network is represented internally by its wiring diagram `I` and the list of update functions `F`, which is far more memory-efficient – especially for sparse networks with few regulators per node.

When a Boolean network is constructed from `F` and `I`, `BoolForge` automatically performs a series of consistency checks to guard against common modeling errors. For example, it verifies that each update function has the correct length, namely 2^n, where n is the number of regulators of the corresponding node as specified in `I`. If any of these checks fail, an informative error is raised immediately, helping ensure that the resulting network is well-defined.

6.5.1 Creating networks from strings

Alternatively, Boolean networks can be specified using a human-readable string representation, where each line defines the update rule of one node. This format closely mirrors the way Boolean models are written in the literature and is often more convenient than manually specifying wiring diagrams and truth tables.

In the example below, each line has the form $x_i = f_i(\text{regulators of } x_i)$, where Boolean operators such as `AND`, `OR`, and `NOT` can be used to define the update functions.

```
string = """
x = y
y = x OR z
z = y
"""

bn_str = bf.BooleanNetwork.from_string(string, separator="=")
print(bn_str.to_truth_table().to_string())
```

```
   x(t)  y(t)  z(t)  x(t+1)  y(t+1)  z(t+1)
0     0     0     0       0       0       0
1     0     0     1       0       1       0
2     0     1     0       1       0       1
3     0     1     1       1       1       1
4     1     0     0       0       1       0
5     1     0     1       0       1       0
6     1     1     0       1       1       1
7     1     1     1       1       1       1
```

Here, the update rule `x = y` specifies that node `x` copies the state of `y`, while `y = x OR z` indicates that node `y` becomes activated (1) whenever `x`, or `z`, or both are active.

From this symbolic description, `BoolForge` automatically:

- extracts the wiring diagram,
- determines the regulators of each node,
- constructs the corresponding Boolean update functions.

Internally, the string representation is converted into the same `(F, I)` representation used throughout the package. As a result, Boolean networks created from strings behave identically to those created explicitly from wiring diagrams and truth tables.

This interface is particularly useful for loading Boolean network models from external sources, such as `.bnet` files, or for quickly prototyping models in an interactive setting.

6.5.2 Interoperability with CANA

`BoolForge` provides native interoperability with the CANA package for the analysis of Boolean functions and Boolean networks [Marcus et al., 2025]. Existing `BoolForge` networks can be converted into `CANA` objects and back without loss of information.

In the example below, we convert a `BoolForge` Boolean network into its `CANA` representation using `to_cana()`, and then reconstruct a new `BoolForge` Boolean network from that `CANA` object.

The final assertion verifies that this round-trip conversion preserves:

- the Boolean update functions,
- the wiring diagram,
- and the variable names.

This guarantees that `BoolForge` and `CANA` can be used interchangeably within a workflow, allowing users to leverage `CANA`'s analytical tools while continuing to build and manipulate models using `BoolForge`.

```
cana_bn = bn.to_cana()
bn_from_cana = bf.BooleanNetwork.from_cana(cana_bn)

assert (
    np.all([np.all(bn.F[i].f == bn_from_cana.F[i].f) for i in range(bn.N)])
    and np.all([np.all(bn.I[i] == bn_from_cana.I[i]) for i in range(bn.N)])
    and np.all(bn.variables == bn_from_cana.variables)
), "BooleanNetwork CANA conversion failed"
```

6.6 Types of nodes in Boolean networks

Nodes in a Boolean network can be classified as follows:

- *Constant nodes*:
  Nodes with constant update functions (always 0 or always 1). These nodes act as parameters and they are eliminated at construction time by substituting their constant value into all dependent update functions.
- *Identity nodes*:
  Nodes whose update function is the identity, i.e., $f(x_i) = x_i$. Their value is determined by the initial condition and remains constant over time. Identity nodes are retained as part of the Boolean network state. They may be viewed as nodes with a self-loop and no other incoming edges.
- *Regulated nodes*:
  Nodes whose update functions depend on one or more other nodes.

```
F = [
    [0, 0, 0, 1],  # regulated
    [0, 1, 1, 1],  # regulated
    [0, 1],        # identity
    [0],           # constant
]

I = [
    [1, 2],        # regulated
    [0, 3],        # regulated
    [2],           # identity
    [],            # constant
]
```

```
bn = bf.BooleanNetwork(F, I)

print("bn.variables:", bn.variables)
print("bn.constants:", bn.constants)
print("bn.I:", bn.I)
print("bn.F:")
for i, f in enumerate(bn.F):
    print(f"  F[{i}] = {f!r}")
```

```
bn.variables: ['x0' 'x1' 'x2']
bn.constants: {'x3': 0}
bn.I: [array([1, 2]), array([0]), array([2])]
bn.F:
  F[0] = BooleanFunction(name='x0', f=[0, 0, 0, 1])
  F[1] = BooleanFunction(name='x1', f=[0, 1])
  F[2] = BooleanFunction(name='x2', f=[0, 1])
```

The constant node is removed, and its value is propagated into downstream update functions.

If we now change the value of the constant node from 0 to 1, the network is constructed in the same way, and the constant value 1 is substituted directly into all downstream update functions, before removal of the constant node.

As a result, the Boolean update functions of downstream nodes may simplify, potentially reducing the number of regulators or changing the logical form of the function. This illustrates how constant nodes act as parameters whose values influence the effective dynamics of the network.

Importantly, this simplification is performed symbolically at construction time and does not depend on the dynamical evolution of the network.

```
F = [
    [0, 0, 0, 1],
    [0, 1, 1, 1],
    [0, 1],
    [1],
]

I = [
    [1, 2],
    [0, 3],
    [2],
    [],
]

bn = bf.BooleanNetwork(F, I)

print("bn.variables:", bn.variables)
print("bn.constants:", bn.constants)
print("bn.I:", bn.I)
print("bn.F:")
for i, f in enumerate(bn.F):
    print(f"  F[{i}] = {f!r}")
```

```
bn.variables: ['x0' 'x1' 'x2']
bn.constants: {'x3': 1}
bn.I: [array([1, 2]), array([0]), array([2])]
bn.F:
  F[0] = BooleanFunction(name='x0', f=[0, 0, 0, 1])
  F[1] = BooleanFunction(name='x1', f=[1, 1])
```

```
F[2] = BooleanFunction(name='x2', f=[0, 1])
```

Although x_1 becomes fixed at 1 after one update, it is not treated as a constant node. In BoolForge, constant nodes are identified by their update functions (always 0 or always 1), not by their long-term dynamical behavior. In other words, BoolForge distinguishes structural constants (defined by update rules) from dynamical constants (states that become fixed along trajectories). Since $x_1 = 0$ remains a valid initial condition, the node is retained as part of the network state.

6.7 Boolean network properties

The class BooleanNetwork inherits basic structural properties and methods from WiringDiagram. In particular, all graph-theoretic attributes of the wiring diagram – such as the number of nodes, in-degrees, and out-degrees – are directly accessible on a Boolean network object.

Moreover, BooleanNetwork inherits visualization utilities from WiringDiagram, including methods for plotting the wiring diagram and its modular structure, using .plot(). This allows users to inspect the topology of a Boolean network independently of the specific update functions.

Beyond these inherited features, BooleanNetwork provides a rich collection of additional methods for analyzing the dynamics, structure, and control properties of Boolean networks. These include functionality for:

- computing fixed points and attractors,
- analyzing transient dynamics and state transition graphs,
- studying robustness and sensitivity to perturbations,
- performing node and edge interventions.

Many of these methods will be introduced and discussed in detail in the following tutorials. Here, we focus only on a few basic and commonly used properties.

```
print("bn.N:", bn.N)
print("bn.indegrees:", bn.indegrees)
print("bn.outdegrees:", bn.outdegrees)
print("bn.variables:", bn.variables)

bn.plot();
```

```
bn.N: 3
bn.indegrees: [2 1 1]
bn.outdegrees: [1 1 2]
bn.variables: ['x0' 'x1' 'x2']
```

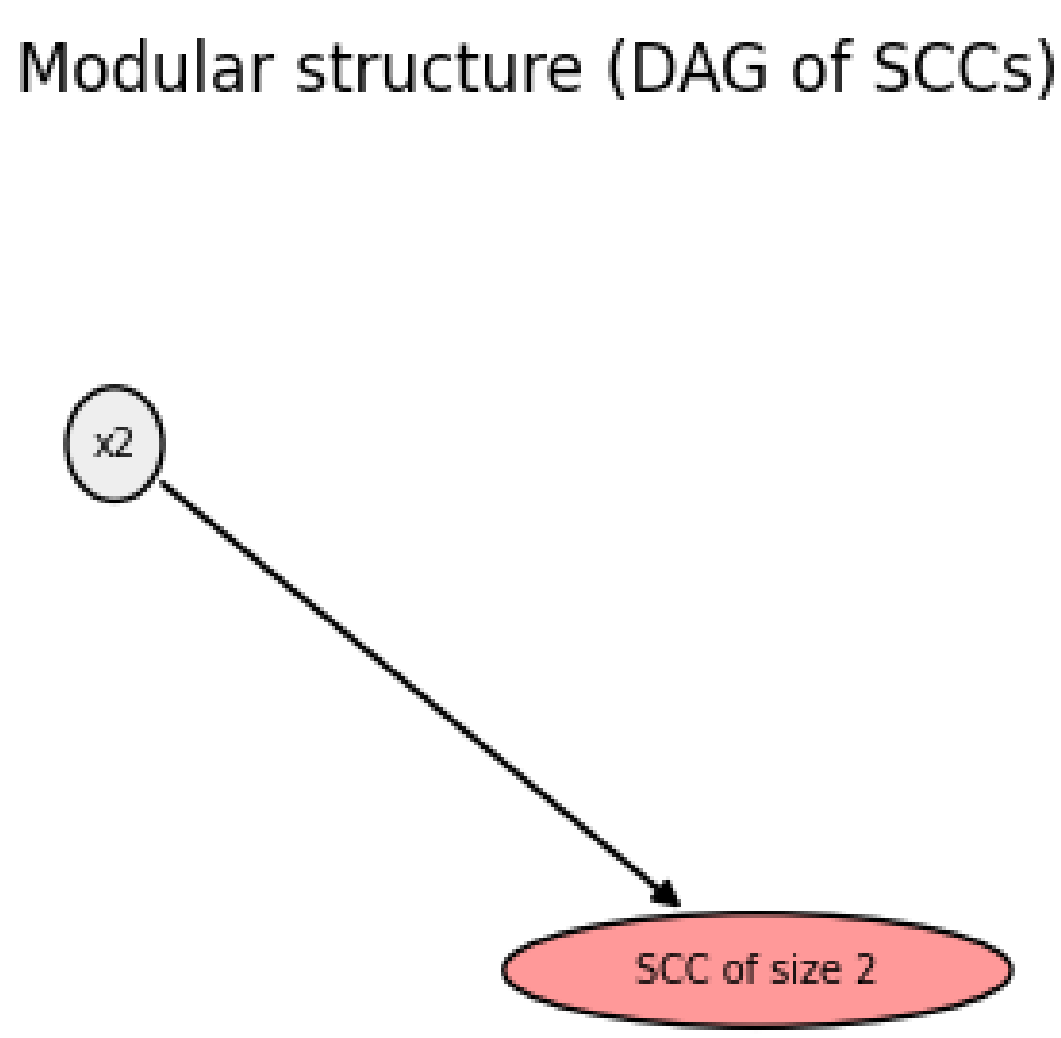

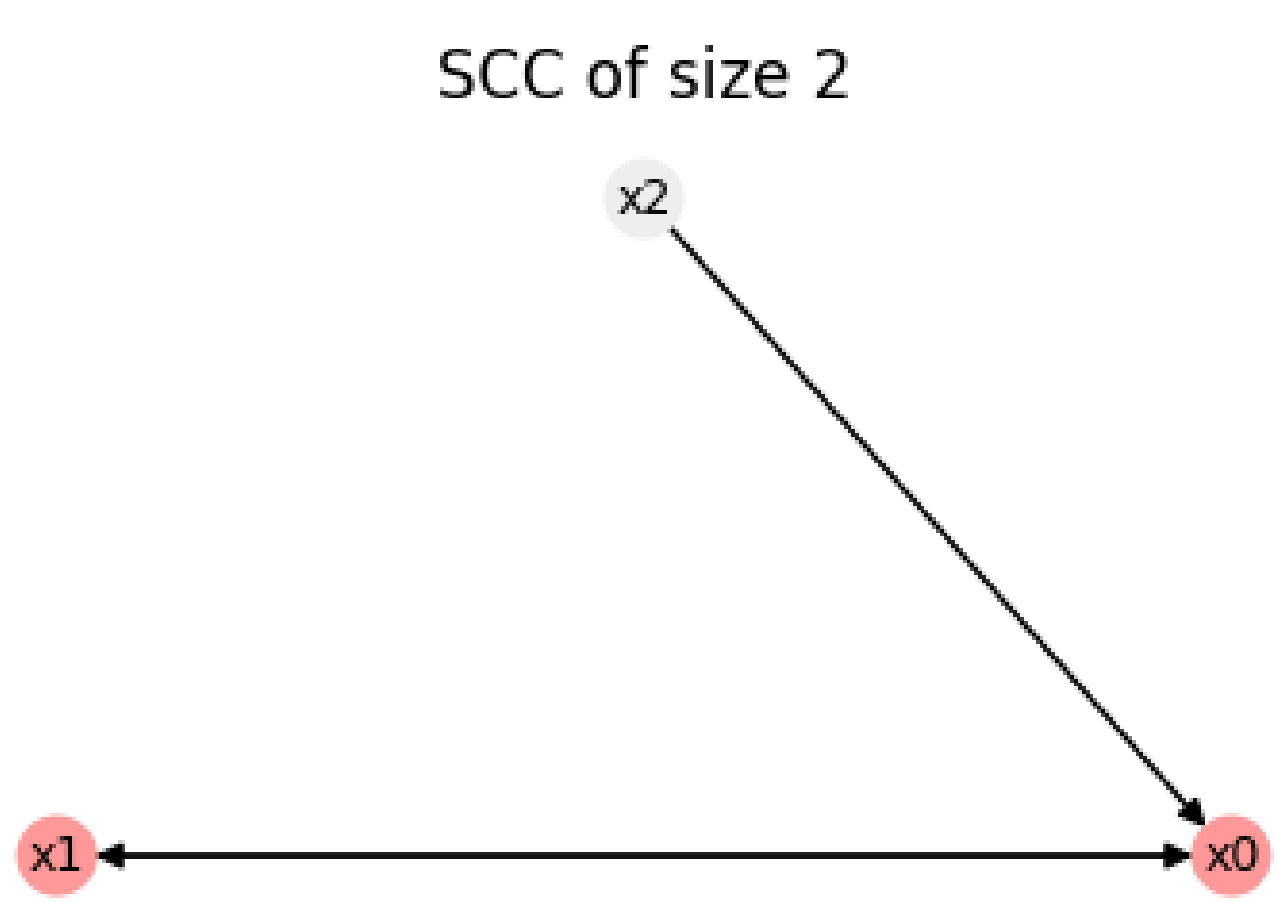

Just like BooleanFunction objects, BooleanNetwork possesses a `.summary()` method, which prints a human-readable overview of basic properties. If more advanced properties have already been computed, e.g., attractors, this information is also displayed (or if the optional keyword `compute_all` is set to True, default False).

```
print(bn.summary())
print()
print(bn.summary(compute_all=True)) #or simply print(bn.summary(True))
```

```
BooleanNetwork
--------------
Number of nodes:              3
Number of regulated nodes:    2
Number of identity nodes:     1
Number of constants (removed):1
Average degree:               1.333
Largest in-degree:            2
Largest out-degree:           2
Regulated nodes:              ['x0', 'x1']
```

```
Identity nodes (inputs):      ['x2']
Constants:                    {'x3': 1}

BooleanNetwork
--------------
Number of nodes:              3
Number of regulated nodes:    2
Number of identity nodes:     1
Number of constants (removed):1
Average degree:               1.333
Largest in-degree:            2
Largest out-degree:           2
Regulated nodes:              ['x0', 'x1']
Identity nodes (inputs):      ['x2']
Constants:                    {'x3': 1}
Number of attractors:         2
Largest basin size:           0.500
Basin size entropy:           0.693
Derrida value:                0.667
Coherence:                    0.667
Fragility:                    0.222
```

The more advanced properties displayed here are the subject of the next two tutorials.

6.8 Manipulation and control of Boolean networks

Identity nodes can represent external inputs or environmental conditions. Fixing their values allows us to study the behavior of the network under specific contexts. BoolForge enables users to obtain a reduced network, in which the identity nodes are set to specific values.

```
cn = bn.get_network_with_fixed_identity_nodes(values_identity_nodes=[0])
print("cn.F:")
for i, f in enumerate(cn.F):
    print(f"  F[{i}] = {f!r}")
print()
print(cn.summary())
```

```
cn.F:
  F[0] = BooleanFunction(name='x0', f=[0, 0])
  F[1] = BooleanFunction(name='x1', f=[1, 1])

BooleanNetwork
--------------
Number of nodes:              2
Number of regulated nodes:    2
Number of constants (removed):2
Average degree:               1.000
Largest in-degree:            1
Largest out-degree:           1
Regulated nodes:              ['x0', 'x1']
Constants:                    {'x2': 0, 'x3': 1}
```

Fixing identity nodes converts them into constant nodes, which are then eliminated via constant propagation. Only the identity nodes are removed from `cn`. Nodes that become dynamically constant after fixing identity nodes (e.g., x_0 and x_1) are retained, since their initial values may still vary. For example, $x_0(t = 0) = 1$ or $x_1(t = 0) = 0$ remain valid initial values, despite the fact that $x_0(t) = 0$ and $x_1(t) = 1$ at any time $t > 0$.

6.8.1 Node controls

Boolean network control is an active area of research (see e.g., Murrugarra et al. [2016] or Borriello and Daniels [2021]). For example, the knock-out of a certain gene can be simulated in a Boolean network by setting this gene to a constant value of zero. Likewise, overexpression can be modeled by setting it to a constant value of one. BoolForge enables users to implement node and edge controls of existing Boolean networks. This provides a simple framework for simulating interventions such as gene knock-outs or overexpression.

To implement node controls, we need to specify which nodes should be controlled and the constant values that they should be set to. As an example, we consider a classical Boolean network model, the three-node repressilator [Elowitz and Leibler, 2000].

```
string = """
A = not B
B = not C
C = not A
"""

bn = bf.BooleanNetwork.from_string(string, separator="=")
bn.plot();
cn = bn.get_network_with_node_controls(indices_controlled_nodes=[2],
                                       values_controlled_nodes=[0])
cn.plot();
print("cn.F:")
for i, f in enumerate(cn.F):
    print(f"  F[{i}] = {f!r}")
print("cn.constants:", cn.constants)
```

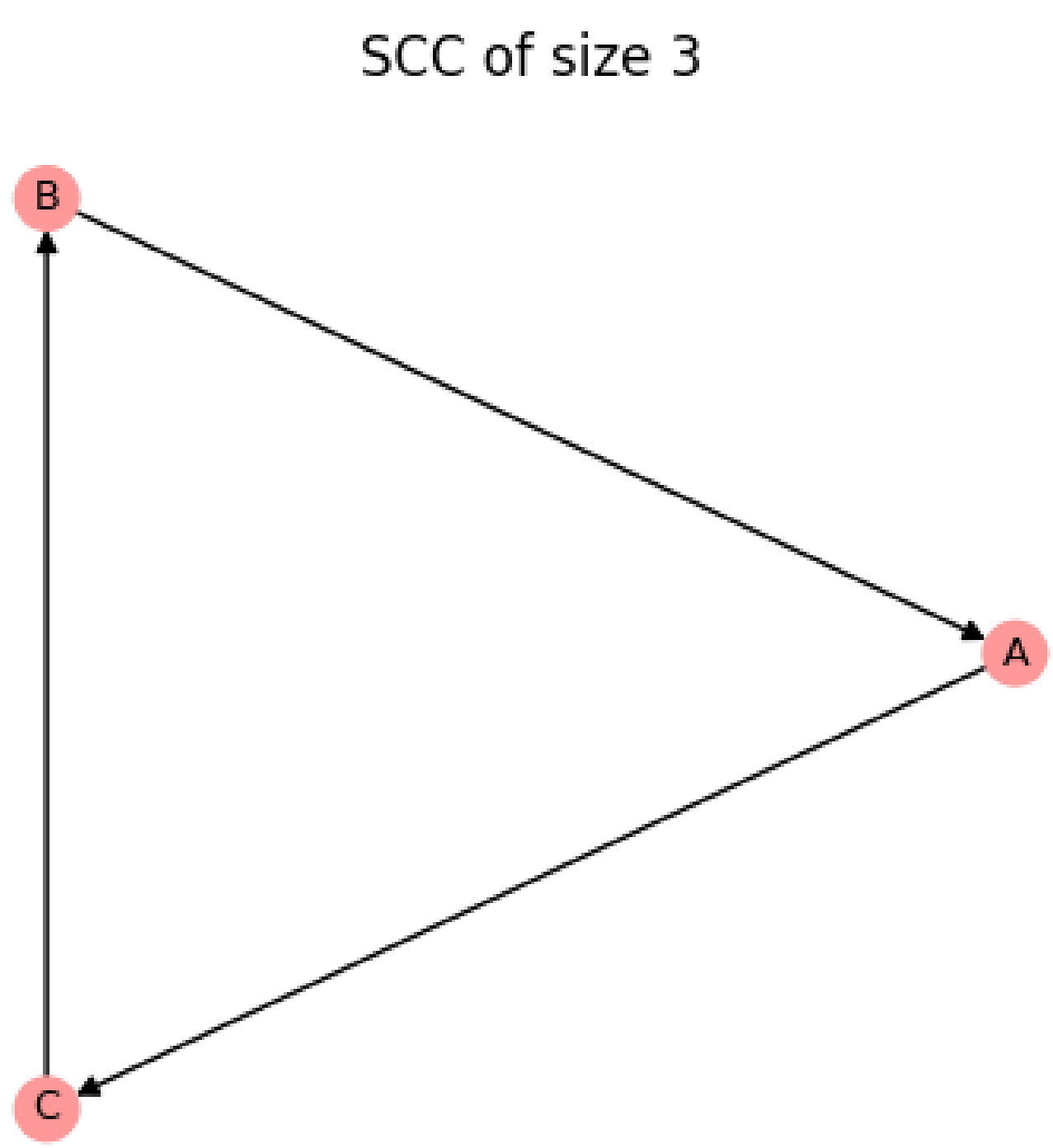

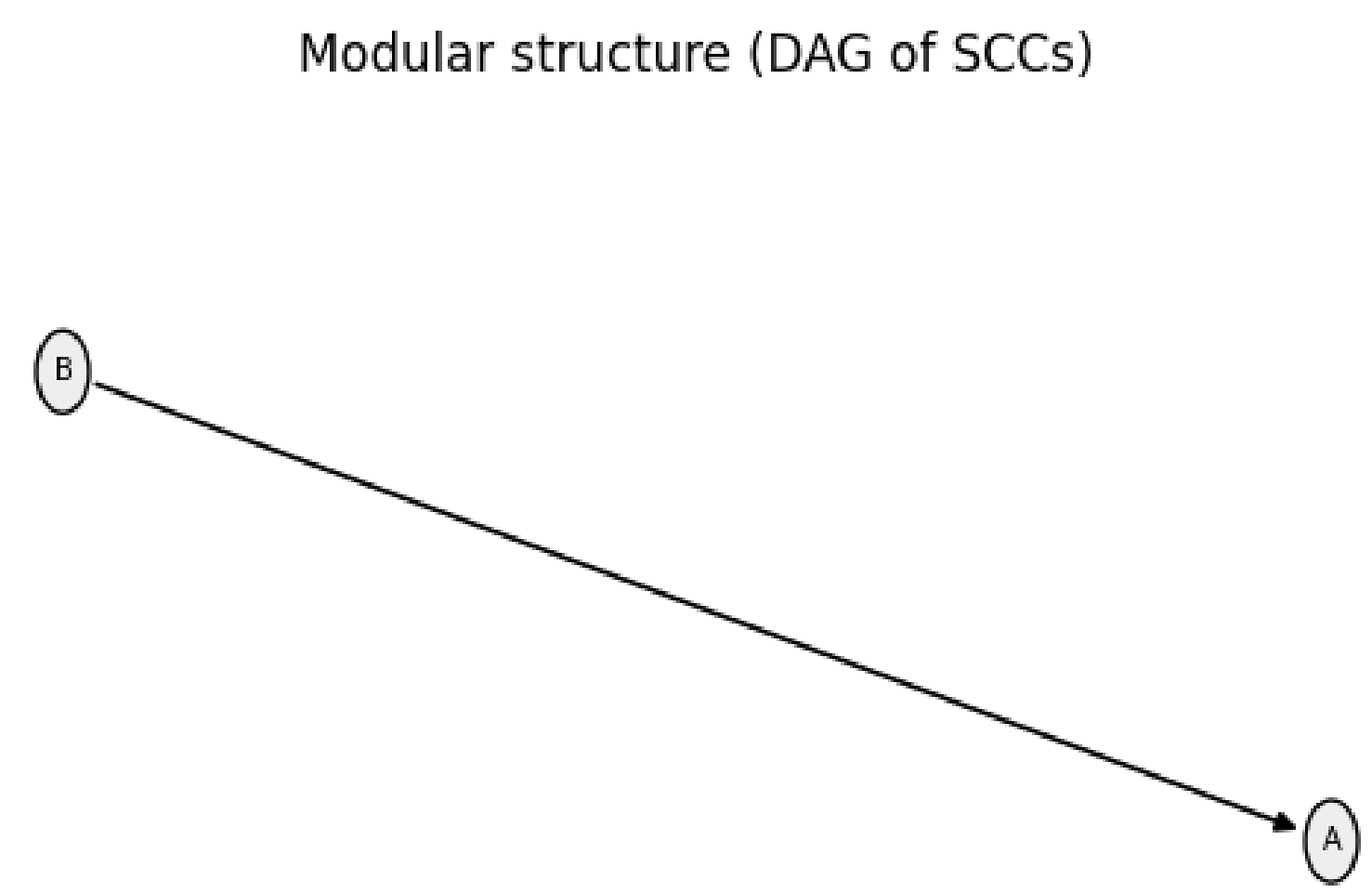


```
cn.F:
  F[0] = BooleanFunction(name='A', f=[1, 0])
  F[1] = BooleanFunction(name='B', f=[1, 1])
cn.constants: {'C': 0}
```

Setting $C = 0$ removes C from the reduced network `cn` (it becomes a constant). Moreover, since $B = \neg C$, we get $B = 1$ always, while the update rule for A is not changed.

6.8.2 Edge controls

Similarly, we can implement edge controls. Edge control removes the influence of a source node on a target node by fixing the source to a specified value within the target's update function. The resulting function is then simplified, and the corresponding edge is removed. As an example, we consider a more connected Boolean network with different types of update rules.

```
string = """
A = B and C
B = A or C
C = A and not B
"""
bn = bf.BooleanNetwork.from_string(string, separator="=")
print("bn.I:", bn.I)
print("bn.F:")
for i, f in enumerate(bn.F):
    print(f"  F[{i}] = {f!r}")
print()

cn = bn.get_network_with_edge_controls(control_targets=[0],
                                       control_sources=[1],
                                       values_edge_controls=[0])
print("cn.I:", cn.I)
print("cn.F:")
for i, f in enumerate(cn.F):
    print(f"  F[{i}] = {f!r}")
```

```
bn.I: [array([1, 2]), array([0, 2]), array([0, 1])]
```

```
bn.F:
  F[0] = BooleanFunction(name='A', f=[0, 0, 0, 1])
  F[1] = BooleanFunction(name='B', f=[0, 1, 1, 1])
  F[2] = BooleanFunction(name='C', f=[0, 0, 1, 0])

cn.I: [array([2]), array([0, 2]), array([0, 1])]
cn.F:
  F[0] = BooleanFunction(name='A', f=[0, 0])
  F[1] = BooleanFunction(name='B', f=[0, 1, 1, 1])
  F[2] = BooleanFunction(name='C', f=[0, 0, 1, 0])
```

By setting $B = 0$ in the regulation of A, we remove B's influence on A. Moreover, since $A = B \wedge C$, we now have $A = 0$ always.

6.9 Outlook

In the remaining tutorials, we build on this foundation to study the dynamical behavior of Boolean networks, including attractors, basins of attraction, and stability under perturbations.

7 Dynamics of Boolean Networks

In this tutorial, we study the *dynamics* of Boolean networks. Building on the construction and structural analysis from previous tutorials, we now focus on characterizing the long-term behavior of Boolean networks.

7.1 What you will learn

You will learn how to:

- simulate Boolean network dynamics under different updating schemes,
- compute and classify attractors,
- analyze basins of attraction,
- relate network structure to dynamical behavior.

7.2 Setup

```
import boolforge as bf
import numpy as np
import pandas as pd
import matplotlib.pyplot as plt
```

7.3 State space of a Boolean network

A Boolean network with N nodes defines a dynamical system on the discrete state space $\{0,1\}^N$.

Each state is a binary vector

$$\mathbf{x} = (x_0, \ldots, x_{N-1}) \in \{0,1\}^N,$$

where x_i denotes the state of node i.

We use a small Boolean network as a running example.

```
string = """
x = y
y = x OR z
z = y
"""

bn = bf.BooleanNetwork.from_string(string, separator="=")

print("Variables:", bn.variables)
print("N:", bn.N)
print("bn.I:", bn.I)
print("bn.F:")
for i, f in enumerate(bn.F):
    print(f"  F[{i}] = {f!r}")
```

```
Variables: ['x' 'y' 'z']
N: 3
bn.I: [array([1]), array([0, 2]), array([1])]
bn.F:
  F[0] = BooleanFunction(name='x', f=[0, 1])
  F[1] = BooleanFunction(name='y', f=[0, 1, 1, 1])
  F[2] = BooleanFunction(name='z', f=[0, 1])
```

All state vectors follow the variable order given by `bn.variables`. For small networks, we can enumerate all 2^N states explicitly.

```
all_states = bf.get_left_side_of_truth_table(bn.N)
print(pd.DataFrame(all_states, columns=bn.variables).to_string())
```

```
   x  y  z
0  0  0  0
1  0  0  1
2  0  1  0
3  0  1  1
4  1  0  0
5  1  0  1
6  1  1  0
7  1  1  1
```

7.4 Dynamics of synchronous Boolean networks

Under *synchronous updating*, all nodes are updated simultaneously, defining a deterministic update map

$$\mathbf{x}(t+1) = F(\mathbf{x}(t)).$$

7.4.1 Exact computation

The update map F can be evaluated directly for any state vector. In BoolForge, this is implemented by the method `update_network_synchronously`. For convenience, Boolean networks are callable, so that `bn(state)` evaluates the update map and is equivalent to `bn.update_network_synchronously(state)`.

```
for state in all_states:
    print(state, "-->", bn(state))
```

```
[0 0 0] --> [0 0 0]
[0 0 1] --> [0 1 0]
[0 1 0] --> [1 0 1]
[0 1 1] --> [1 1 1]
[1 0 0] --> [0 1 0]
[1 0 1] --> [0 1 0]
[1 1 0] --> [1 1 1]
[1 1 1] --> [1 1 1]
```

This output matches the synchronous truth table representation:

```
print(bn.to_truth_table().to_string())
```

```
   x(t)  y(t)  z(t)  x(t+1)  y(t+1)  z(t+1)
0     0     0     0       0       0       0
1     0     0     1       0       1       0
2     0     1     0       1       0       1
3     0     1     1       1       1       1
4     1     0     0       0       1       0
5     1     0     1       0       1       0
6     1     1     0       1       1       1
7     1     1     1       1       1       1
```

Each state has exactly one successor, so the dynamics consist of transient trajectories leading into *attractors* (steady states or cycles).

In this example, the network has:

- two steady states: $(0, 0, 0)$ and $(1, 1, 1)$,
- one cyclic attractor of length 2: $(0, 1, 0) \leftrightarrow (1, 0, 1)$.

7.4.2 Exhaustive attractor computation

BoolForge contains a dedicated method to identify all attractors of a network under synchronous update.

```
dict_dynamics = bn.get_attractors_synchronous_exact()
```

The returned dictionary contains:

- `STG`: the synchronous state transition graph,
- `NumberOfAttractors`,
- `Attractors`,
- `AttractorID`,
- `BasinSizes`.

For computational reasons, binary states in $\{0, 1\}^N$ are identified by their decimal representation. The state transition graph can be decoded as follows:

```
for state in range(2   bn.N):
    next_state = dict_dynamics["STG"][state]
    print(
        state,
        "=",
        bf.dec2bin(state, bn.N),
        "-->",
        next_state,
        "=",
        bf.dec2bin(next_state, bn.N),
    )
```

```
0 = [0, 0, 0] --> 0 = [0, 0, 0]
1 = [0, 0, 1] --> 2 = [0, 1, 0]
2 = [0, 1, 0] --> 5 = [1, 0, 1]
3 = [0, 1, 1] --> 7 = [1, 1, 1]
4 = [1, 0, 0] --> 2 = [0, 1, 0]
5 = [1, 0, 1] --> 2 = [0, 1, 0]
6 = [1, 1, 0] --> 7 = [1, 1, 1]
7 = [1, 1, 1] --> 7 = [1, 1, 1]
```

After repeated updates, the system settles into periodic behavior. That is, irrespective of the initial state, an attractor is reached. The list of all attractors (in decimal representation) can be displayed.

```
print(dict_dynamics['Attractors'])
```

```
[[0], [2, 5], [7]]
```

Attractors can be printed in binary representation:

```
for attractor in dict_dynamics["Attractors"]:
    print(f"Attractor of length {len(attractor)}:")
    for state in attractor:
        print(state, bf.dec2bin(state, bn.N))
    print()
```

```
Attractor of length 1:
0 [0, 0, 0]

Attractor of length 2:
2 [0, 1, 0]
5 [1, 0, 1]

Attractor of length 1:
7 [1, 1, 1]
```

The information which state transitions to which attractor is stored in a dictionary. Here, the indices correspond to the list of attractors in `dict_dynamics['Attractors']`.

```
for state_dec,attr_id in enumerate(dict_dynamics['AttractorID']):
    print(state_dec,'--> attractor',attr_id,
          'which is',dict_dynamics['Attractors'][attr_id])
```

```
0 --> attractor 0 which is [0]
1 --> attractor 1 which is [2, 5]
2 --> attractor 1 which is [2, 5]
3 --> attractor 2 which is [7]
4 --> attractor 1 which is [2, 5]
5 --> attractor 1 which is [2, 5]
6 --> attractor 2 which is [7]
7 --> attractor 2 which is [7]
```

Finally, the basin size of each attractor is determined by the number of states that eventually transition to an attractor. By definition, the sum of all basin sizes is always 2^N. To simplify the comparison of the basin size distribution for networks of different size, `BoolForge` normalizes the basin sizes by default.

```
print(dict_dynamics['BasinSizes'])
```

```
[0.125 0.5   0.375]
```

From the previous two outputs, we see that there is no state (other than 000) that eventually transitions to 000. Half the states transition to the 2-cycle, while 3 out of 8 states transition to the attractor 111.

7.4.3 Monte Carlo simulation

For larger networks, exhaustive enumeration is infeasible. Monte Carlo simulation approximates the attractor landscape.

```
dict_dynamics = bn.get_attractors_synchronous(n_simulations=1000)
print('Discovered attractors:',dict_dynamics['Attractors'])
print('Basin size approximation:',dict_dynamics['BasinSizesApproximation'])
```

```
Discovered attractors: [[7], [2, 5], [0]]
Basin size approximation: [0.383 0.498 0.119]
```

The simulation returns additional information:

- sampled initial states,
- the number of timeouts (trajectories not reaching an attractor before timeout).

```
for key in dict_dynamics:
    print(key)
```

```
Attractors
NumberOfAttractorsLowerBound
BasinSizesApproximation
AttractorID
InitialSamplePoints
STG
NumberOfTimeouts
```

In the absence of timeouts: If an attractor has relative basin size q, the probability that it is found from m random initializations is $1-(1-q)^m$.

```
qs = [0.0001, 0.001, 0.01, 0.1]
ms = np.logspace(0, 4, 1000)

fig, ax = plt.subplots()
for q in qs:
    ax.semilogx(ms, 1 - (1 - q)   ms, label=str(q))

ax.legend(title=r"$q$", frameon=False)
ax.set_xlabel("number of initial states ($m$)")
ax.set_ylabel("probability attractor of basin size q is found")
plt.show()
```

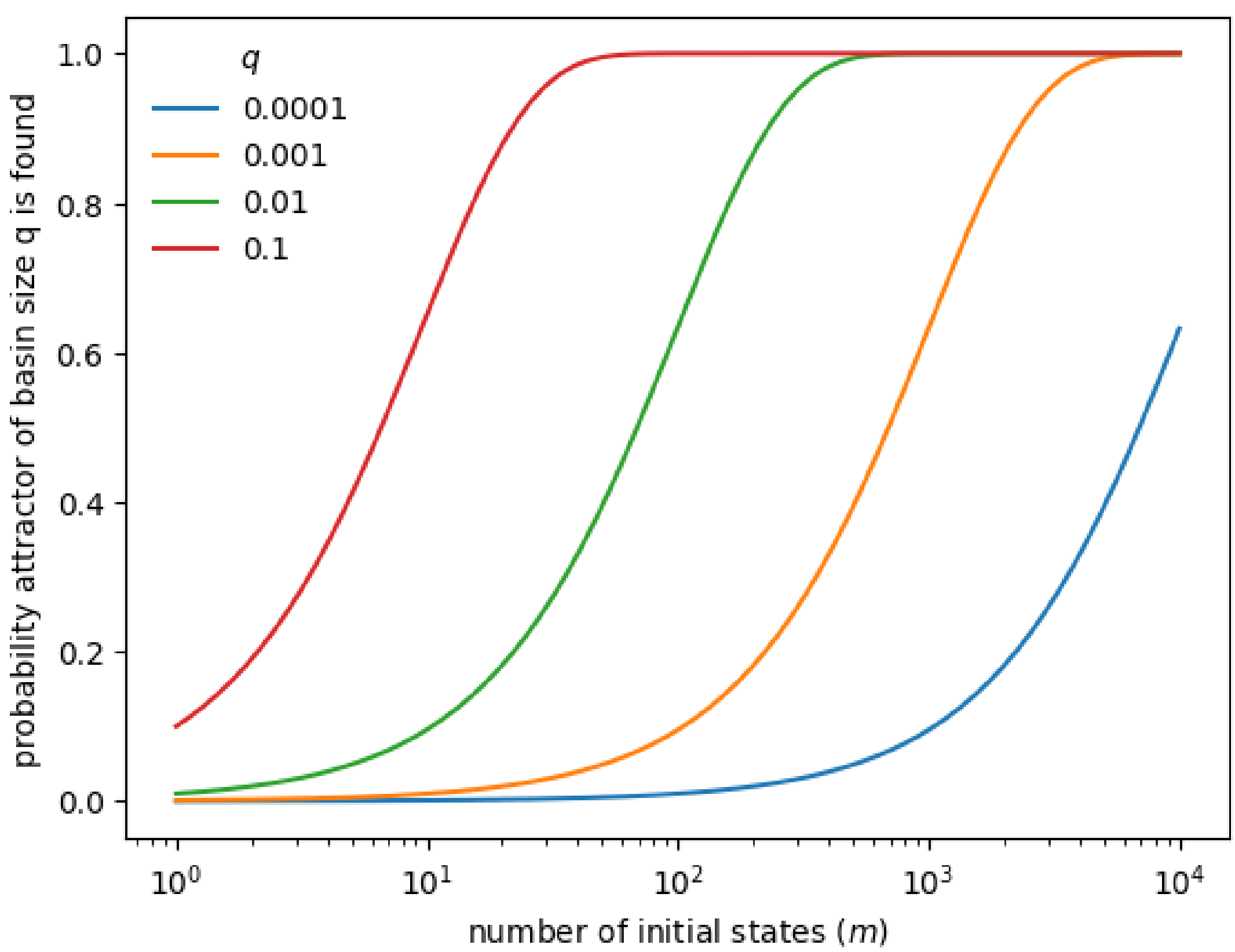

7.5 Dynamics of asynchronous Boolean networks

Synchronous updating is computationally convenient but biologically unrealistic. Asynchronous updating assumes that only one node changes at a time.

7.5.1 Attractors under general asynchronous update

BoolForge can compute attractors under *general asynchronous updating*, where at each step only a single node updates according to its Boolean rule. Under synchronous updating, the dynamics are deterministic, while under asynchronous updating, the dynamics are generally stochastic. That implies that the notion of attractors needs to be adapted. In particular, synchronous limit cycles typically disappear under asynchronous updating, while steady states remain unchanged. Instead, under asynchronous updating, the long-term behavior is characterized by the *terminal strongly connected components* (SCCs) of the asynchronous state transition graph.

```
terminal_sccs = bn.get_terminal_sccs_asynchronous_exact()
print('Terminal strongly connected components:', terminal_sccs)
```

```
Terminal strongly connected components: [[0], [7]]
```

The result reveals the same two steady states as in the synchronous case. However, the limit cycle observed under synchronous updating disappears under asynchronous dynamics.

In addition, BoolForge can return the *full asynchronous state transition graph*.

```
STG_async = bn.get_asynchronous_transition_matrix()
print(STG_async)
```

```
<Compressed Sparse Row sparse matrix of dtype 'float32'
    with 14 stored elements and shape (8, 8)>
  Coords    Values
  (0, 0)    1.0
  (1, 0)    0.5
  (1, 3)    0.5
  (2, 0)    0.3333333432674408
  (2, 3)    0.3333333432674408
  (2, 6)    0.3333333432674408
  (3, 7)    1.0
  (4, 0)    0.5
  (4, 6)    0.5
  (5, 1)    0.3333333432674408
  (5, 4)    0.3333333432674408
  (5, 7)    0.3333333432674408
  (6, 7)    1.0
  (7, 7)    1.0
```

The state transition graph, a *sparse transition matrix* of a Markov chain, describes all possible state transitions, together with their probabilities.

From this matrix, BoolForge can compute the *absorption probabilities*, i.e., the probability that a trajectory starting from a given state eventually reaches a specific terminal SCC.

```
print(np.round(bn.get_absorption_probabilities_exact(),4))
```

```
[[1.     0.    ]
 [0.5    0.5   ]
 [0.3333 0.6667]
 [0.     1.    ]
 [0.5    0.5   ]
 [0.3333 0.6667]
 [0.     1.    ]
 [0.     1.    ]]
```

This shows that the state 001 (1 in decimal representation) reaches both steady states with equal probability. On the other hand, the states 011 (decimal = 3) and 110 (decimal = 6) always eventually settle in the steady state 111, the second attractor in `terminal_sccs`.

From the absorption probabilities, the size of each basin of attraction can also be readily computed as the column-wise sum of these probabilities. This metric, together with many other dynamical properties, is returned by the method `get_terminal_sccs_and_robustness_asynchronous_exact`, which we investigate in more detail in the next tutorial.

```
dict_dynamics = bn.get_terminal_sccs_and_robustness_asynchronous_exact()
print('Basin sizes:',dict_dynamics['BasinSizes'])
```

```
Basin sizes: [0.33333334 0.6666666 ]
```

7.5.2 Monte Carlo approximation

As in the synchronous case, `BoolForge` also contains a Monte Carlo routine for sampling asynchronous dynamics. However, this routine is currently limited to identifying steady states and approximating their basin sizes. It does not identify terminal SCCs. For this task, specialized tools such as pystablemotifs are recommended [Rozum et al., 2022].

BoolForge's simulation framework provides:

- a lower bound on the number of steady states,
- approximate basin size distributions,

```
dict_dynamics = bn.get_steady_states_asynchronous(n_simulations=500)
print('Discovered steady states:', dict_dynamics['SteadyStates'])
print('Number of steady states (lower bound):',dict_dynamics['NumberOfSteadyStatesLowerBound'])
print('Basin size approximation:',dict_dynamics['BasinSizesApproximation'])
```

```
Discovered steady states: [7, 0]
Number of steady states (lower bound): 2
Basin size approximation: [0.68 0.32]
```

7.5.3 Sampling from a fixed initial condition

In biological Boolean network models, a specific state $\mathbf{x} \in \{0,1\}^N$ is frequently considered the initial state, e.g., corresponding to the G0 phase of the cell cylce. To enable exploration of the stochastic trajectories from a specific state, BoolForge contains the following method.

```
dict_dynamics = bn.get_steady_states_asynchronous_given_one_initial_condition(
    initial_condition=[0, 0, 1], n_simulations=500
)
print('Discovered steady states:', dict_dynamics['SteadyStates'])
print('Number of steady states (lower bound):',dict_dynamics['NumberOfSteadyStatesLowerBound'])
print('Basin size approximation:',dict_dynamics['BasinSizesApproximation'])
```

```
Discovered steady states: [0, 7]
Number of steady states (lower bound): 2
Basin size approximation: [0.526 0.474]
```

Note the equivalent analysis under synchronous update is trivial because the dynamics are deterministic and the long-term behavior when starting in a specific initial condition can be found by

```
dict_dynamics = bn.get_attractors_synchronous(n_simulations=1,
                                              initial_sample_points=[[0,0,1]],
                                              initial_sample_points_are_vectors=True)
for key, value in dict_dynamics.items():
    print(f"{key}: {value}")
```

```
Attractors: [[2, 5]]
NumberOfAttractorsLowerBound: 1
BasinSizesApproximation: [1.]
AttractorID: {2: 0, 5: 0}
InitialSamplePoints: [[0, 0, 1]]
STG: {1: 2}
NumberOfTimeouts: 0
```

7.6 Summary

In this tutorial you learned how to:

- simulate Boolean network dynamics,
- compute synchronous attractors exactly and approximately,
- analyze basin sizes,
- compute asynchronous attractors (terminal SCCs) and absorption probabilities exactly,
- identify, by simulation, some (but possible not all) steady states for large asynchronously updated Boolean networks.

This concludes the function- and network-level analysis. Subsequent tutorials focus on analyzing stability to perturbations, control analysis, and ensemble experiments.

8 Perturbation and sensitivity analysis of Boolean networks

In this tutorial, we study how Boolean networks respond to perturbations. Rather than implementing perturbations manually, we leverage BoolForge's built-in robustness and sensitivity measures.

8.1 What you will learn

You will learn how to:

- quantify robustness and fragility of Boolean networks under synchronous and asynchronous update schemes,
- interpret basin-level and attractor-level robustness measures,
- perform exact and approximate robustness computations, and
- compute Derrida values as a measure of dynamical sensitivity.

Together, these tools allow us to assess dynamical stability and resilience of Boolean network models in a principled and computationally efficient way.

8.2 Setup

```
import boolforge as bf
import pandas as pd
```

We reuse the small Boolean network from the previous tutorial as a running example.

```
string = """
x = y
y = x OR z
z = y
"""

bn = bf.BooleanNetwork.from_string(string, separator="=")

print("Variables:", bn.variables)
print("Number of nodes:", bn.N)
```

```
Variables: ['x' 'y' 'z']
Number of nodes: 3
```

8.3 Exact attractors and robustness measures

BoolForge provides a single method that computes:

- all attractors,
- basin sizes,
- overall network coherence and fragility,
- basin-level coherence and fragility, and
- attractor-level coherence and fragility.

These quantities are defined via systematic single-bit perturbations in the Boolean hypercube and can be computed *exactly* for small networks.

```
results_exact_sync = bn.get_attractors_and_robustness_synchronous_exact()
for key in results_exact_sync.keys():
    print(key)
```

```
Attractors
NumberOfAttractors
BasinSizes
AttractorID
Coherence
Fragility
BasinCoherences
BasinFragilities
AttractorCoherences
AttractorFragilities
```

For convenience, information about the dynamics (attractors, basin sizes, etc), described in detail in the previous tutorial, is also returned by this method.

```
print("Number of attractors:", results_exact_sync["NumberOfAttractors"])
print("Attractors (decimal states):", results_exact_sync["Attractors"])
print("Eventual attractor of each state:", results_exact_sync["AttractorID"])

print("Basin sizes:", results_exact_sync["BasinSizes"])
```

```
Number of attractors: 3
Attractors (decimal states): [[0], [2, 5], [7]]
Eventual attractor of each state: [0 1 1 2 1 1 2 2]
Basin sizes: [0.125 0.5   0.375]
```

8.4 Network-, basin- and attractor-level robustness in synchronous networks

Robustness can be resolved at different structural levels. Network-level metrics report the average robustness of any network state when subjected to perturbation.

```
print("Overall coherence:", results_exact_sync["Coherence"])
print("Overall fragility:", results_exact_sync["Fragility"])
```

```
Overall coherence: 0.3333333333333333
Overall fragility: 0.3333333333333333
```

The same robustness metrics, coherence and fragility, can also be averaged across a smaller set of states, e.g., all states in one basin of attraction (see Bavisetty et al. [2025]), or an even smaller set of states, e.g., all states that form an attractor (see Bavisetty et al. [2026]).

```
df_basins = pd.DataFrame({
    "BasinSizes": results_exact_sync["BasinSizes"],
    "BasinCoherences": results_exact_sync["BasinCoherences"],
    "BasinFragilities": results_exact_sync["BasinFragilities"],
}, index = list(map(str,results_exact_sync["Attractors"])))

df_attractors = pd.DataFrame({
    "AttractorCoherences": results_exact_sync["AttractorCoherences"],
    "AttractorFragilities": results_exact_sync["AttractorFragilities"],
}, index = list(map(str,results_exact_sync["Attractors"])))

print("Basin-level robustness:")
print(df_basins)

print("Attractor-level robustness:")
print(df_attractors)
```

```
Basin-level robustness:
        BasinSizes  BasinCoherences  BasinFragilities
[0]          0.125         0.000000          0.500000
[2, 5]       0.500         0.333333          0.333333
[7]          0.375         0.444444          0.277778
Attractor-level robustness:
        AttractorCoherences  AttractorFragilities
[0]                0.000000              0.500000
[2, 5]             0.333333              0.333333
[7]                0.666667              0.166667
```

Interpretation:

- *Coherence* measures the fraction of single-bit perturbations that do *not* change the final attractor [Willadsen et al., 2008].
- *Fragility*, only computed for synchronously updated networks, measures how much the attractor state changes [Park et al., 2023].

The robustness metrics considered thus far describe how a single perturbation affects the network dynamics in the long-term, i.e., at the attractor. These metrics are very meaningful biologically because attractors typically correspond to cell types of phenotypes.

It turns out that attractors in synchronously updated biological networks are often less stable than their basins, a phenomenon explored in detail in Tutorial 10.

8.5 Network-, basin- and attractor-level robustness in asynchronous networks

Coherence can also be defined for asynchronously updated networks, but fragility cannot, because the notion of a single attractor state is not well-defined in the asynchronous case. `BoolForge` therefore only computes coherence for asynchronous networks. Similar to the synchronous case, `get_terminal_sccs_and_robustness_asynchronous_exact` returns attractor (terminal strongly connected component) information and information on robustness measures at the network, basin, and attractor levels.

```
results_exact_async = bn.get_terminal_sccs_and_robustness_asynchronous_exact()
print("Terminal strongly connected components (asynchronous attractors):",
      results_exact_async["TerminalSCCs"])
print('Number of terminal strongly connected components:',
      results_exact_async["NumberOfTerminalSCCs"])
print('Length of terminal strongly connected components:',
      results_exact_async["LengthOfTerminalSCCs"])
print('Basin sizes (asynchronous):',
      results_exact_async["BasinSizes"])

print("Overall coherence (asynchronous):",
      results_exact_async["Coherence"])
print("Basin-level coherence (asynchronous):",
      results_exact_async["BasinCoherences"])
print("Attractor-level coherence (asynchronous):",
      results_exact_async["TerminalSCCCoherencesStationary"])
```

```
Terminal strongly connected components (asynchronous attractors): [[0], [7]]
Number of terminal strongly connected components: 2
Length of terminal strongly connected components: [1 1]
Basin sizes (asynchronous): [0.33333334 0.6666666 ]
Overall coherence (asynchronous): 0.6111110912428964
Basin-level coherence (asynchronous): [0.41666666 0.70833334]
Attractor-level coherence (asynchronous): [0.44444445 0.88888888]
```

8.6 Approximate robustness for larger synchronous networks

For larger networks, exact enumeration of all 2^N states is infeasible. BoolForge therefore provides a Monte Carlo approximation for synchronously updated networks that samples random initial conditions and perturbations.

```
results_approx = bn.get_attractors_and_robustness_synchronous(n_simulations=500)

print("Number of attractors (lower bound):", results_approx["NumberOfAttractorsLowerBound"])
print("Approximate coherence:", results_approx["CoherenceApproximation"])
print("Approximate fragility:", results_approx["FragilityApproximation"])
```

```
Number of attractors (lower bound): 3
Approximate coherence: 0.342
Approximate fragility: 0.329
```

Even when only using 500 random initial states, the approximate values closely match the exact ones. For networks with 20 to 30 nodes, simulation becomes the only feasible option due to the exponential growth of state space.

Note that the approximate method is not yet implemented for asynchronously updated networks, but this is planned for a future release, as the theory is being extended to the asynchronous case.

8.7 Derrida value: dynamical sensitivity

An older and very popular robustness metric, the Derrida value, measures how perturbations *propagate* after one synchronous update [Derrida and Pomeau, 1986]. It is defined as the expected Hamming distance between updated states that initially differed in exactly one bit.

BoolForge includes routines for the exact calculation and estimation of Derrida values. For networks with low degree, the exact calculation is strongly preferable. It is faster and more accurate.

```
derrida_exact = bn.get_derrida_value(exact=True)
derrida_approx = bn.get_derrida_value(n_simulations=2000)

print("Exact Derrida value:", derrida_exact)
print("Approximate Derrida value:", derrida_approx)
```

```
Exact Derrida value: 1.0
Approximate Derrida value: 1.0
```

Interpretation:

- Small Derrida values indicate ordered, stable dynamics.
- Large Derrida values indicate sensitive or chaotic dynamics.

Derrida values are closely related to average sensitivity of the update functions, and provide a complementary notion of robustness.

8.8 Summary

In this tutorial you learned how to:

- compute exact robustness measures for small Boolean networks,
- interpret coherence and fragility at network, basin, and attractor levels,
- approximate robustness measures for larger networks, and
- assess dynamical sensitivity using the Derrida value.

In Tutorial 9, we will finally analyze biological Boolean network models and design ensemble experiments.

9 Random Boolean network generation

This tutorial demonstrates how to generate *random Boolean networks with controlled structural and functional properties* using BoolForge. This ability enables ensemble studies, which are exemplified in the next tutorial.

9.1 What you will learn

In this tutorial you will learn how to generate random Boolean networks with

- prescribed structural properties (e.g., degree, degree distribution, strongly connected),
- prescribed functional properties (e.g., canalization, bias),

It is strongly recommended to complete Tutorials 4 and 5 on random function generation first.

9.2 Setup

```
import boolforge as bf
import numpy as np
import matplotlib.pyplot as plt
```

9.3 Generating random wiring diagrams

The function `random_network(N, n, *args)` generates a random N-node Boolean network with degree parameter `n`. The generation follows a two-step process:

- A random wiring diagram is created using `random_wiring_diagram(N, n, *args)`.
- Random Boolean functions with prescribed properties are generated using `random_function(n, *args)`, which was discussed in depth in Tutorials 4 and 5.

We first consider only the structural parameters that concern the generation of the random wiring diagram. In the absence of optional arguments, the in-degree distribution is assumed to be constant. That is, each node in the network is regulated by `n` nodes.

```
N = 5
n = 2

W = bf.random_wiring_diagram(N, n, rng=2)

W.plot();
```

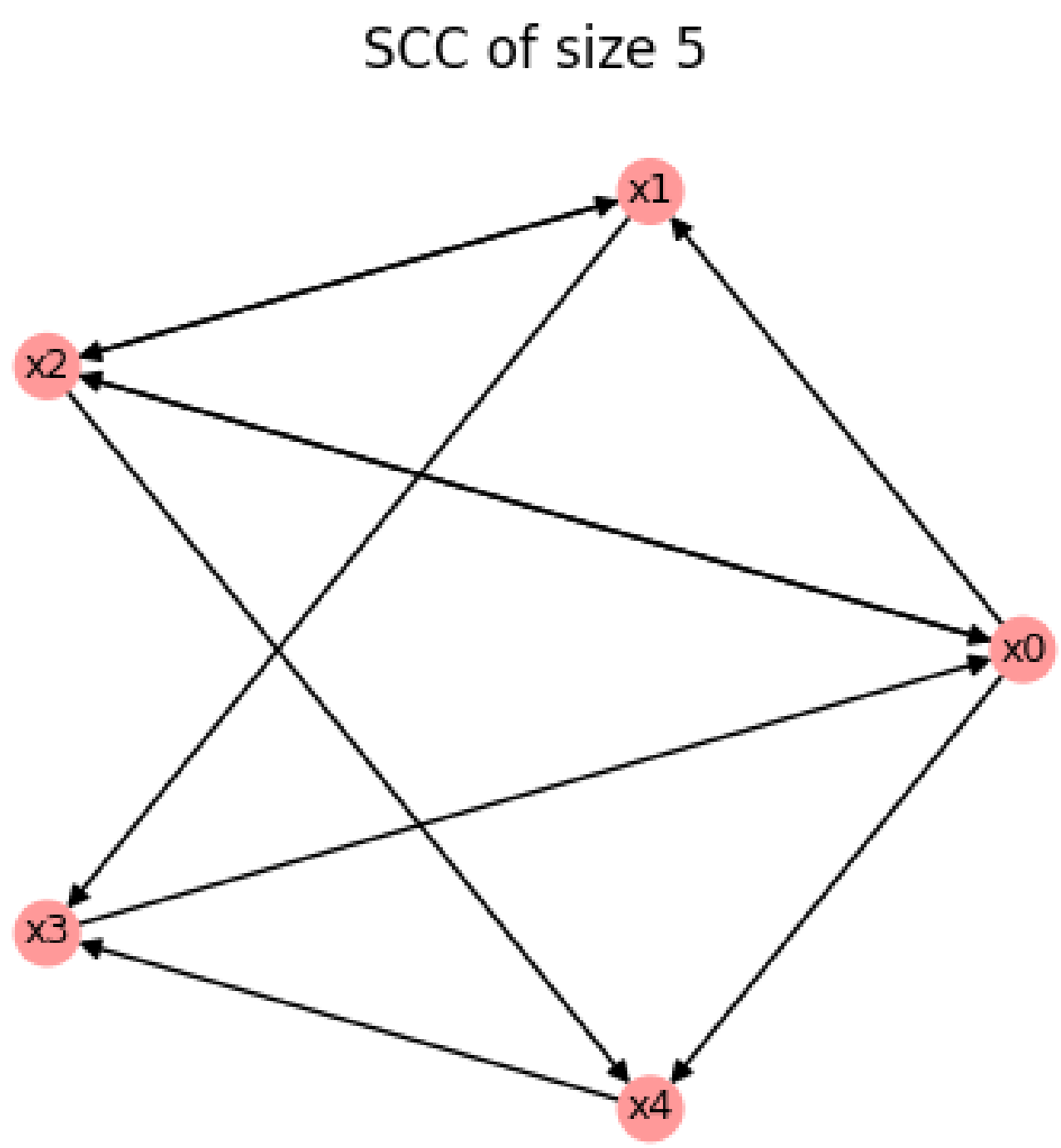

The argument `rng` seeds the random number generator, ensuring reproducible results.

The rest of this tutorial describes the various constraints / optional arguments. Each optional argument restricts the family of networks from which `random_wiring_diagram()` and `random_network()` samples.

9.3.1 Allowing self-regulation

BoolForge selects the `n` regulators of each node uniformly at random from the set of all other nodes. Thus, self-regulation is disallowed by default. Setting `allow_self_loops=True` allows nodes to regulate themselves.

```
N = 5
n = 2

W = bf.random_wiring_diagram(N,n,allow_self_loops=True,rng = 2)

W.plot();
```

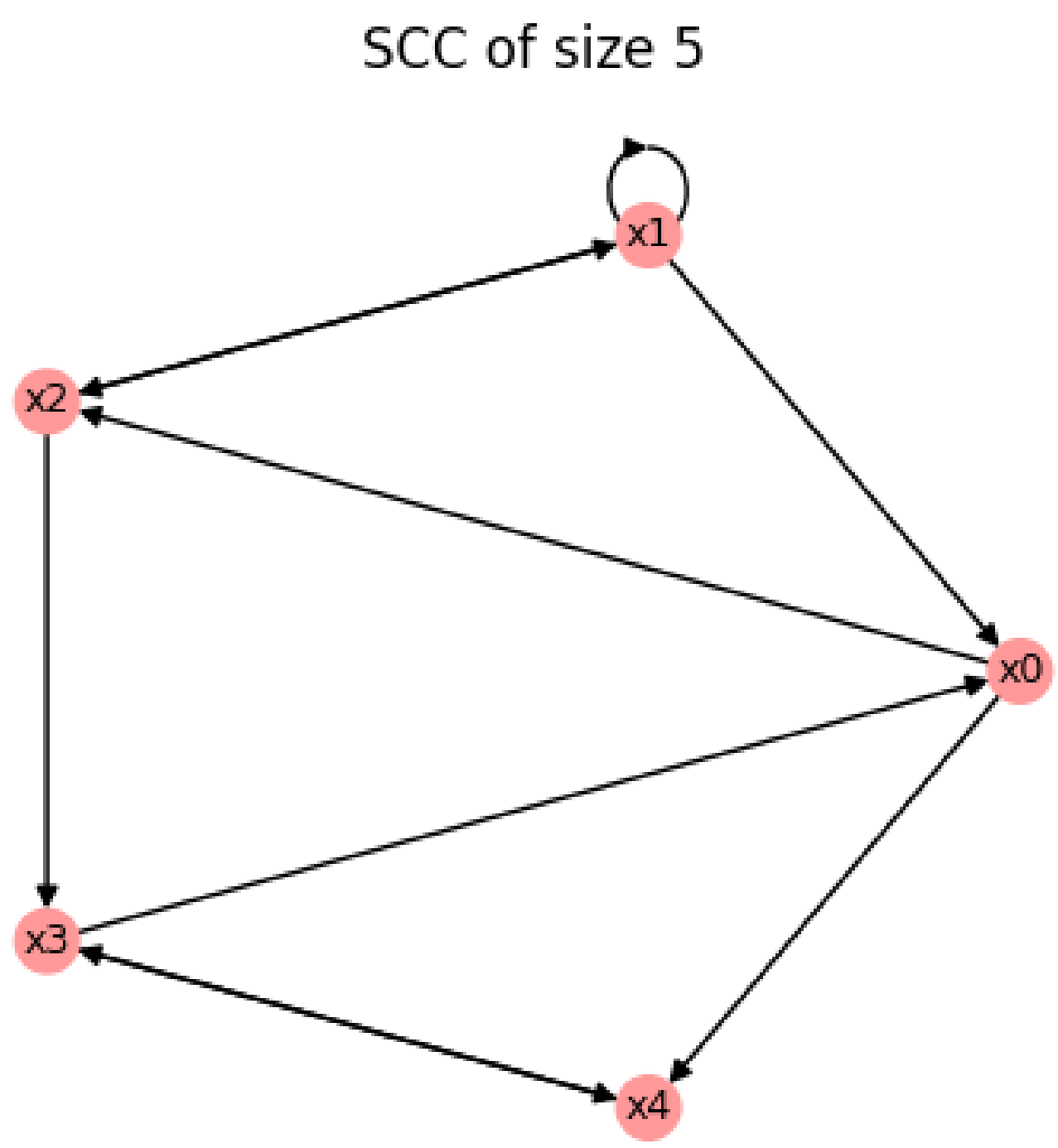

9.3.2 Poisson in-degree distributions

Classical random Boolean network theory (NK Kauffman models) assume a fixed in-degree, the default in BoolForge. However, this is a strong assumption since the in-degree in curated biological Boolean network models often appears approximately Poisson distributed. BoolForge provides the option to generate random wiring diagrams with Poisson distributed in-degree, using the optional parameter `indegree_distribution`.

```
N = 5
n = 2

W = bf.random_wiring_diagram(N,n,indegree_distribution='poisson',rng = 5)

W.plot();
```

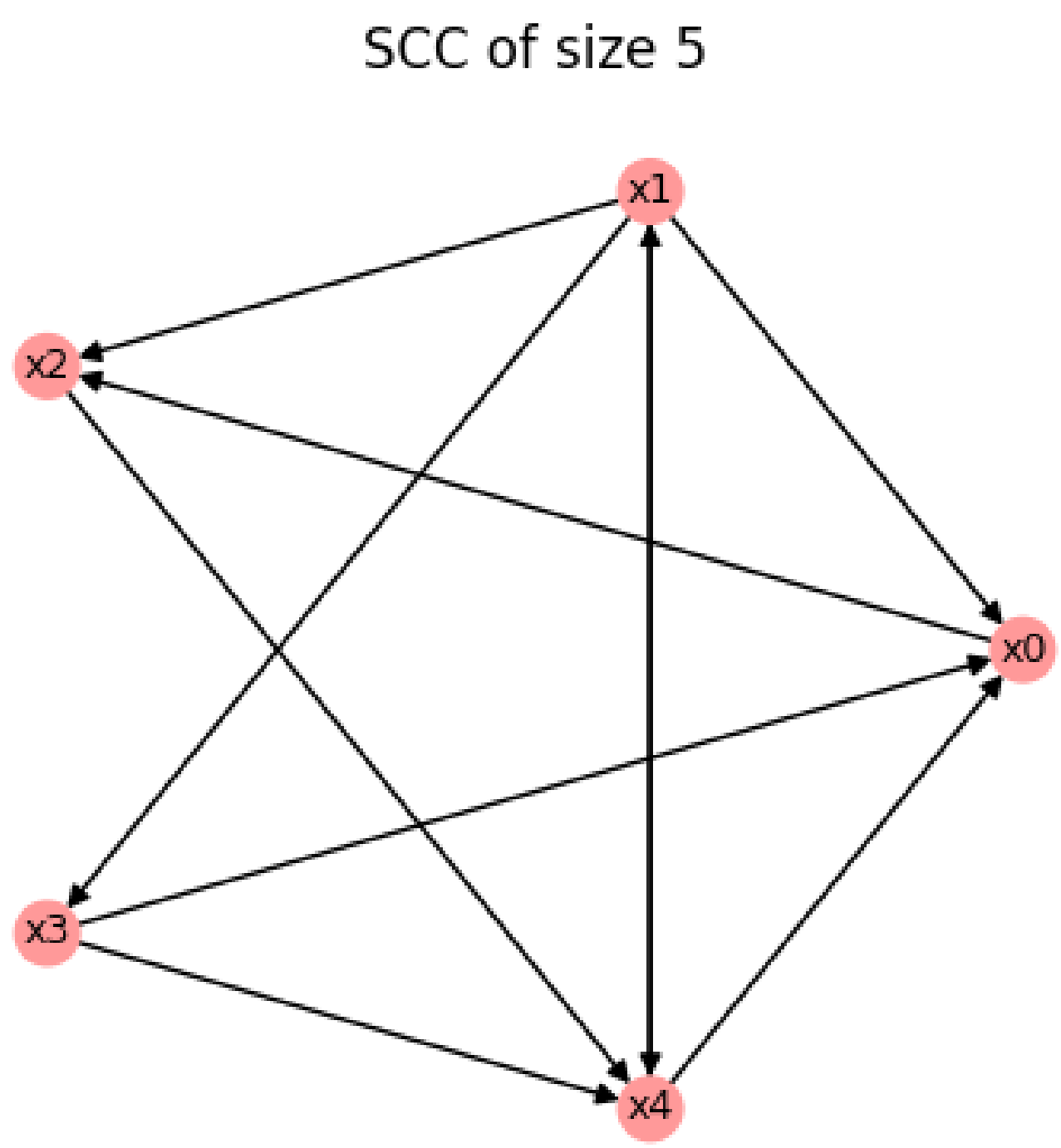

We see that some nodes (x_1 and x_3) are only regulated by one node, while others (x_0 and x_4) possess three regulators each.

When using a Poisson-distributed in-degree, the in-degree of every node is always at least 1. This avoids the artificial creation of identity nodes (with in-degree 0).

9.3.3 Avoiding output nodes

In general, it is possible that some nodes in a generated Boolean network will not regulate other nodes. By setting `min_out_degree_one=True`, we can force every node to regulate at least one node. That is, output nodes can be disallowed.

9.3.4 Strong connectedness

The wiring diagram of the generated Boolean network may or may not be strongly connected. Setting `strongly_connected=True` (default False) forces strong connectedness. Uniform sampling among strongly connected networks cannot be achieved by a simple construction method. BoolForge therefore generates candidate networks and rejects them until a strongly connected network is obtained.

Careful: When the number of nodes `N` is large and the degree `n` is small, this may take a long time. The number of unsuccessful attempts before raising an error is controlled by the optional parameter `max_strong_connectivity_attempts`.

9.3.5 Fixed wiring diagrams

All optional parameters discussed thus far describe properties of the wiring diagram. Instead of generating a new wiring diagram, an existing one (e.g., from a curated biological network model) can be passed directly to `random_network`.

In that case, `random_network(I, *args)` does not require `N` and `n`, because these quantities are inferred from the wiring diagram, provided via optional parameter `I`. As described in detail in Tutorial 6, `I` can be either a `WiringDiagram` object or a list of lists describing the regulators of each node.

For example, using the previously generated wiring diagram, we can write

```
bn = bf.random_network(I=W)
```

This feature allows multiple Boolean networks with different update functions to be generated on the same wiring diagram.

9.4 Specifying functional constraints

Once the wiring diagram is generated, the number of nodes `N` and the in-degree of each node are determined. In step 2, `random_network` now repeatedly calls `random_function` to generate the random Boolean functions. The optional parameters regulating the functional constraints are practically identical to the ones discussed in depth in Tutorial 4, with one important distinction: Most parameters can be sequences of length `N`, in order to specify distinct functional behavior for the different nodes.

In the following, we summarize the key concepts.

9.4.1 Parity functions

If `parity=True` (default False), parity functions (also known as linear functions) are chosen for all nodes, yielding a linear Boolean network (see Chandrasekhar et al. [2023]). Note that for any degree `n`, there are only two parity functions.

9.4.2 Canalizing functions

If a specific `layer_structure` is provided, all functions possess at least these canalizing layers.

```
bn = bf.random_network(N=4,n=3,layer_structure=[1],rng = 2)
for f in bn.F:
    print(f,f.get_layer_structure()['LayerStructure'])
```

```
[0 1 1 0 0 0 0 0] [1]
[0 0 1 1 0 1 1 1] [1, 2]
[0 0 0 0 0 0 0 1] [3]
[1 1 1 0 1 1 1 1] [3]
```

As we see, it is however possible for some functions to randomly possess more canalizing variables in a larger and/or more layers. To ensure `layer_structure` is interpreted as exact layer structure, set `exact_depth=True`.

```
bn = bf.random_network(N=4,n=3,layer_structure=[1],exact_depth=True,rng = 2)
for f in bn.F:
    print(f,f.get_layer_structure()['LayerStructure'])
```

```
[0 1 1 0 0 0 0 0] [1]
[1 0 1 1 0 1 1 1] [1]
[1 1 0 1 0 1 1 1] [1]
[0 1 1 0 1 1 1 1] [1]
```

Rather than specifying the exact layer structure, we can also describe the desired *canalizing depth* (i.e., the number of conditionally canalizing variables) via `depth`. As before, the optional argument `exact_depth` (default False) determines if `depth` is interpreted as exact canalizing depth, or as minimum canalizing depth.

```
#Boolean network whose rules all have minimal canalizing depth 1
bn1 = bf.random_network(N=4,n=3,depth=1,exact_depth=False,rng = 2)
for f in bn1.F:
    print(f.get_canalizing_depth(),f)
```

```
print()

#Boolean network whose rules all have exact canalizing depth 1
bn2 = bf.random_network(N=4,n=3,depth=1,exact_depth=True,rng = 2)
for f in bn2.F:
    print(f.get_canalizing_depth(),f)
```

```
1 [0 1 1 0 0 0 0 0]
3 [0 0 0 0 0 0 1 0]
3 [1 1 0 0 1 1 1 0]
3 [0 1 1 1 0 0 0 0]

1 [0 1 1 0 0 0 0 0]
1 [1 0 0 0 0 0 1 0]
1 [1 1 0 1 0 1 1 1]
1 [0 0 1 0 1 0 0 0]
```

Most optional parameters (e.g., `n`, `depth`, `layer_structure`, `bias`, `absolute_bias`) can also be specified as sequences of length `N`. In that case, each entry applies to one node in the network, allowing different functional constraints for different nodes.

```
bn = bf.random_network(
    N=4,
    n=[3,3,2,2],
    depth=[3,1,2,0],
    exact_depth=True,
    rng=2
)

for f in bn.F:
    print(f.get_canalizing_depth(),f)
```

```
3 [1 1 1 1 0 1 1 1]
1 [1 1 0 1 0 1 1 1]
2 [1 1 0 1]
0 [0 1 1 0]
```

9.4.3 Biased functions

When `parity=False` and all canalization parameters are also at their default values, `random_network` generates each update function with a specified *bias*, i.e.

- probability of output 1: `bias`
- probability of output 0: `1-bias`

The unbiased case (`bias=0.5`) is the default. Instead of the bias, users can also specify the absolute bias to generate functions with a bimodal Hamming weight distribution. For BoolForge to use the parameter provided via `absolute_bias`, `use_absolute_bias=True` is required. The default is `use_absolute_bias=False`, i.e., by default `bias` is used, resulting in a unimodal Hamming weight distribution.

```
N = 1000 #network size
n = 4   #constant in-degree

bn1 = bf.random_network(N=N,n=n,bias=0.75)
bn2 = bf.random_network(N=N,n=n,absolute_bias=0.5,use_absolute_bias=True)
bn3 = bf.random_network(N=N,n=n,absolute_bias=0.5)
bns = [bn1,bn2,bn3]
```

```
labels = ["bias = 0.75", "absolute bias = 0.5", "bias = 0.5 (balanced)"]
possible_hamming_weights = np.arange(2 n + 1)
width = 0.3

fig, ax = plt.subplots()
for i,bn in enumerate(bns):
    count = np.zeros(2 n + 1)
    for f in bn.F:
        count[f.hamming_weight] += 1
    ax.bar(possible_hamming_weights - width + i * width,
           count / N,
           width=width, label=labels[i])

ax.legend(frameon=False)
ax.set_xticks(possible_hamming_weights)
ax.set_xlabel("Hamming weight")
ax.set_ylabel("Proportion of update functions");
```

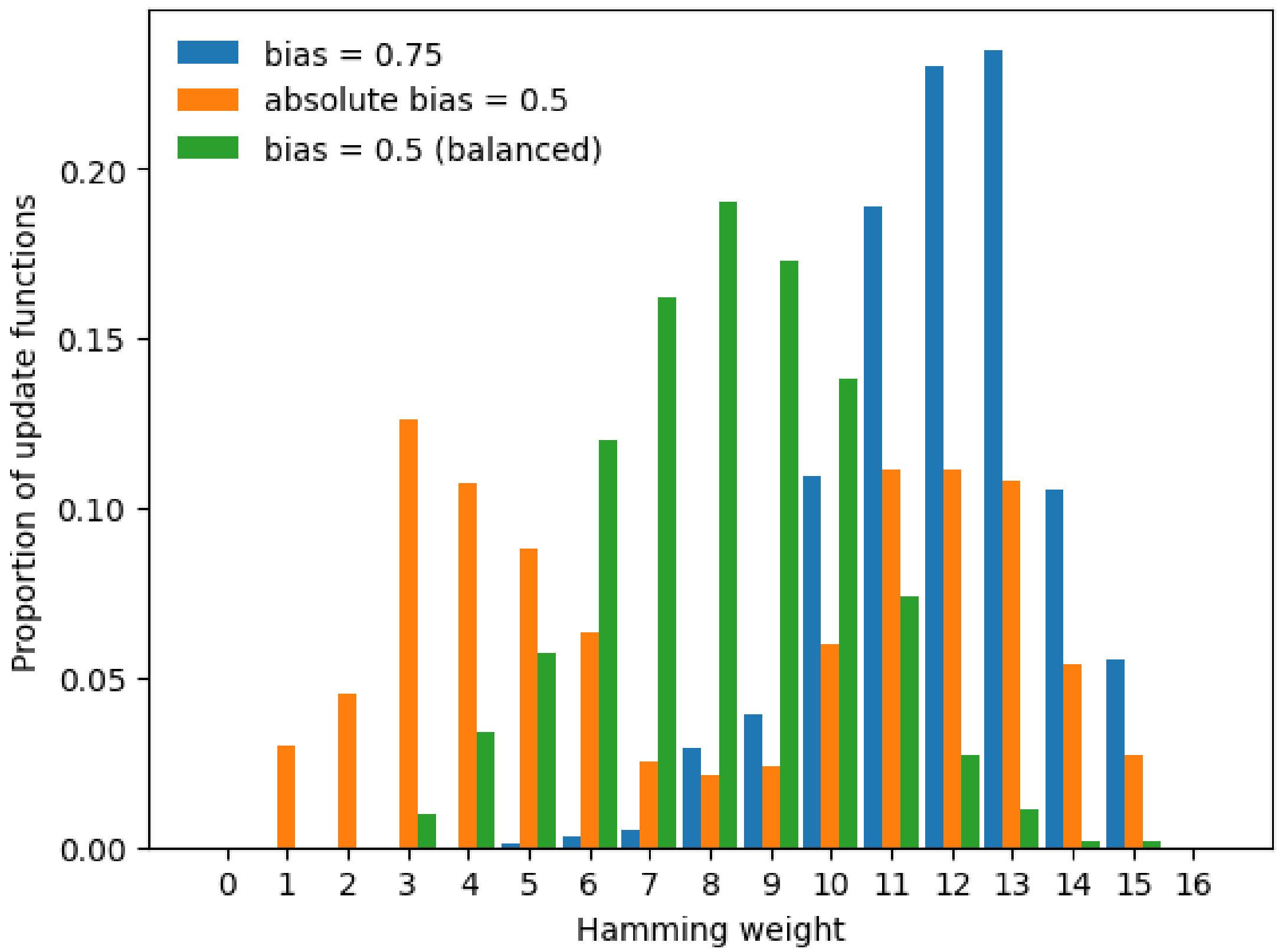

9.5 Summary

In this tutorial you learned how to:

- generate random wiring diagrams with prescribed structural constraints,
- generate, for each node in a wiring diagram, random update functions with prescribed functional constraints.

In the next tutorial, we will explore several situations, in which the ability to generate large ensembles of controlled random Boolean networks is very useful.

10 Ensemble experiments with random Boolean networks

This tutorial demonstrates how BoolForge's ability to generate *random Boolean networks with controlled structural and functional properties* is essential for many types of studies. Specifically, it enables:

1. Null model comparisons:
   Are biological networks structurally or dynamically different from random networks?
2. Ensemble studies: How do structural properties such as degree or canalization affect network dynamics?

10.1 What you will learn

In this tutorial you will learn how to generate random Boolean networks with:

- specific structural properties (e.g., degree, degree distribution, strongly connected),
- prescribed functional properties (e.g., canalization, bias),

It is strongly recommended to complete Tutorials 4 and 5 on random function generation first.

10.2 Setup

```
import boolforge as bf
import numpy as np
import matplotlib.pyplot as plt
```

10.3 NK Kauffman networks

One of the classical models of complex systems is the *NK random Boolean network* introduced by Stuart Kauffman.

In this model:

- The network contains N nodes.
- Each node is regulated by k inputs.
- Each update function is generated randomly with *bias p*, i.e.
  - probability of output 1: `p`
  - probability of output 0: `1-p`

A key theoretical result due to Derrida and Pomeau [1986] predicts how a single-node perturbation propagates in large random Boolean networks. They showed that if two network states differ in one node, the expected number of differences after one update step is $2kp(1-p)$.

If this value is

- < 1, then perturbations decrease on average (ordered regime)
- > 1, then perturbations increase on average (chaotic regime)
- $= 1$, then perturbations remain on average of equal size (critical boundary)

The expected number of propagated perturbations is called the *Derrida value*.

```
N = 100          # network size
ks = range(1,5)  # constant in-degree
n_networks = 50  # ensemble size
p = 0.5          # bias p: probability of ones in truth table

derrida_values = []
for k in ks:
    derrida_values.append([])
    for _ in range(n_networks):
```

```
        bn = bf.random_network(N, k, bias = p, allow_degenerate_functions=True)
        derrida_values[-1].append( bn.get_derrida_value(exact=True) )

plt.boxplot(derrida_values, positions=list(ks))
plt.axhline(1, linestyle="--", color="gray", label="critical value")
plt.plot(ks, [2*k*p*(1-p) for k in ks], "o-", label=r"$2kp(1-p)$ (annealed theory)")
plt.xlabel("Constant in-degree k")
plt.ylabel("Derrida value")
plt.legend(frameon=False);
```

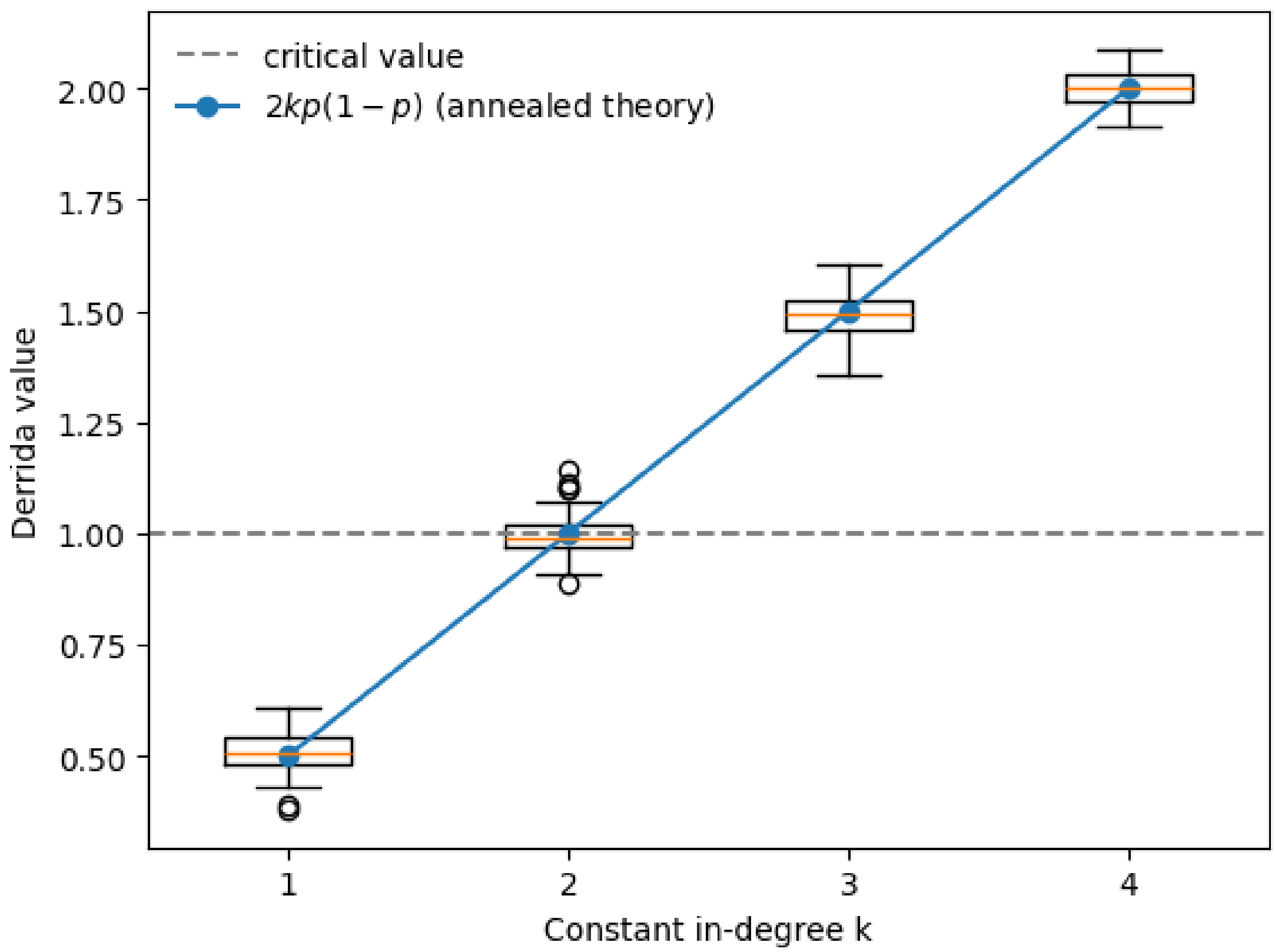

The numerical results closely follow the theoretical prediction $2kp(1-p)$ derived under the *annealed approximation*. The phase transition occurs when the Derrida value crosses 1.

For unbiased Boolean functions (with bias $p = 0.5$), the theory predicts the critical connectivity $k = 2$, which we also observe in this BoolForge ensemble experiment.

We encourage the reader to vary the bias p in the above example. As the bias becomes more extreme, the Derrida value declines and networks with higher connectivity exhibit critical dynamics.

10.4 BoolForge philosophy: regulatory functions are non-degenerate

The classical NK model assumes that a Boolean function with k inputs may *not actually depend on all of them*. Such functions are called *degenerate*.

While this assumption is natural in statistical physics models (e.g. spin glasses), it is biologically questionable. In gene regulatory networks, an input typically represents a *specific regulatory interaction*. If a transcription factor does not affect the gene, it should not appear as an input in the first place. *Therefore BoolForge assumes non-degenerate Boolean functions by default.*

Degeneracy occurs frequently for small input sizes:

- $k = 1$: 2 out of 4 functions are degenerate (50%)
- $k = 2$: 6 out of 16 functions are degenerate
- larger k: degeneracy becomes increasingly rare

Disallowing degenerate functions therefore mainly affects sparse networks, precisely the regime most biological networks operate in (typical average in-degree ≈ 2-3).

We now repeat the previous experiment, *disallowing degenerate functions*.

```
derrida_values = []
for k in ks:
    derrida_values.append([])
    for _ in range(n_networks):
        bn = bf.random_network(N, k, bias = p, allow_degenerate_functions=False)
        derrida_values[-1].append( bn.get_derrida_value(exact=True) )

plt.boxplot(derrida_values, positions=list(ks))
plt.axhline(1, linestyle="--", color="gray", label="critical value")
plt.plot(ks, [2*k*p*(1-p) for k in ks], "o-", label=r"$2kp(1-p)$ (annealed theory)")
plt.xlabel("Constant in-degree k")
plt.ylabel("Derrida value")
plt.legend(frameon=False);
```

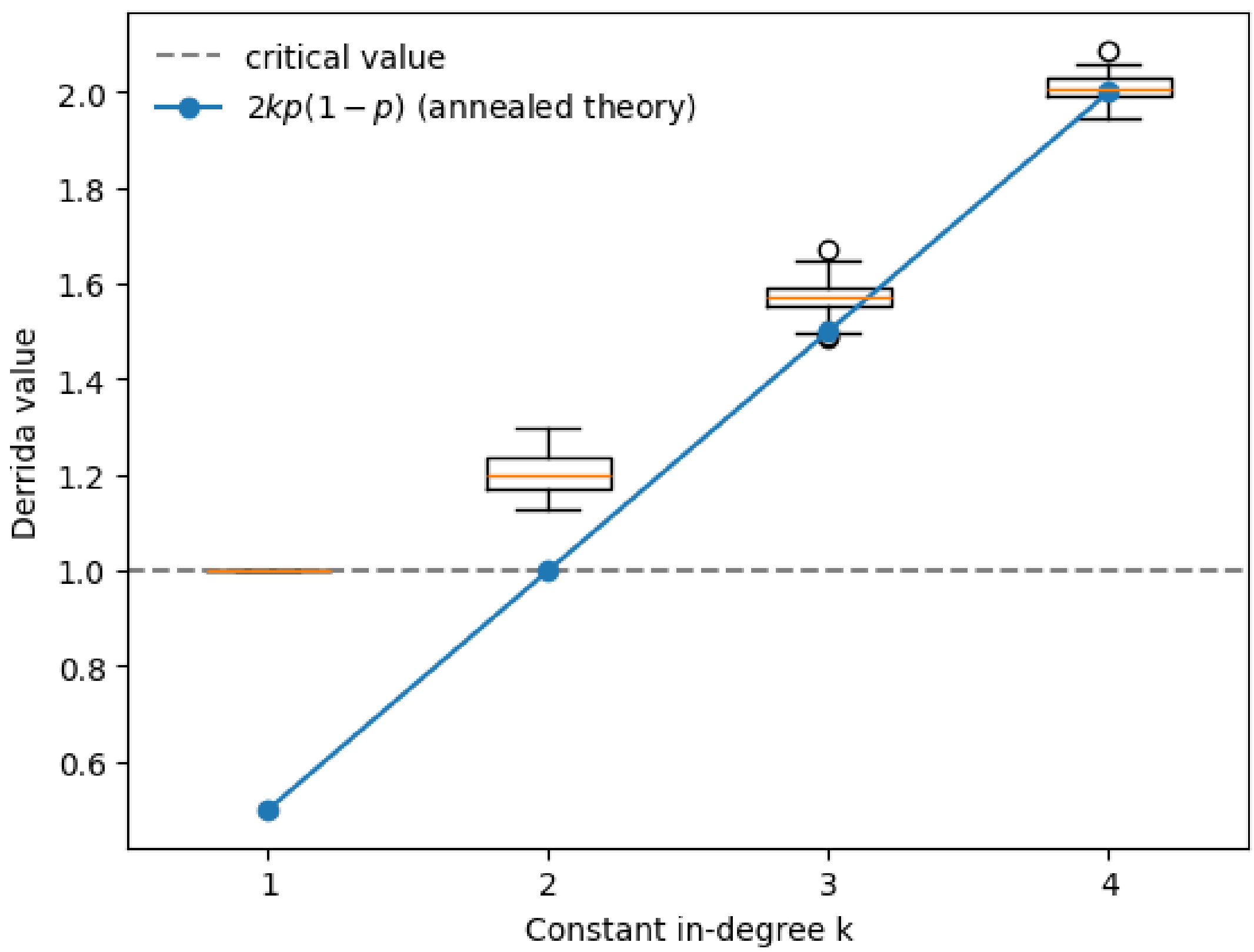

The behavior changes substantially. For *unbiased, non-degenerate Boolean networks (with bias $p = 0.5$)* the phase transition occurs already at $k = 1$, rather than $k = 2$, as predicted by the classical NK theory.

This illustrates how biologically motivated modeling assumptions can significantly affect the predicted dynamical regime of Boolean networks.

10.5 Random networks with prescribed canalization

A major advantage of BoolForge is its ability to generate Boolean functions with *controlled canalization properties*. This is important because canalization is a common feature of biological regulatory networks.

To display the impact of the canalizing layer structure, we generate ensembles of Boolean networks of fixed size and fixed in-degree, which are governed by nested canalizing functions of variable layer structure.

```
N = 12          # network size
n = 5           # constant in-degree
n_networks = 100 # ensemble size

all_hamming = np.arange(1, 2   (n - 1), 2)
all_abs_bias = 2 * np.abs(all_hamming/2 n - 0.5)

number_attractors = []
number_steady_states = []
for i, w in enumerate(all_hamming):
    layer_structure = bf.hamming_weight_to_ncf_layer_structure(n, w)
    number_attractors.append([])
    number_steady_states.append([])
    for _ in range(n_networks):
        bn = bf.random_network(N, n, layer_structure=layer_structure)
        attr_info = bn.get_attractors_synchronous_exact()
        n_attractors = attr_info['NumberOfAttractors']
        number_attractors[-1].append( n_attractors )
        number_steady_states[-1].append(
            sum([len(a)==1 for a in attr_info['Attractors']])
        )

fig,ax = plt.subplots()
mean, std = np.mean(number_attractors,1), np.std(number_attractors,1)
ax.plot(all_abs_bias, mean, 'rx:', label='attractors')
ax.fill_between(all_abs_bias, mean-std, mean+std, color='r', alpha = 0.2)
mean, std = np.mean(number_steady_states,1), np.std(number_steady_states,1)
ax.plot(all_abs_bias, mean, 'bo--', label='steady states')
ax.fill_between(all_abs_bias, mean-std, mean+std, color='b', alpha = 0.2)
ax.set_xlabel("Absolute bias")
ax.set_ylabel("Number")
ax.legend(frameon=False,loc='best');
```

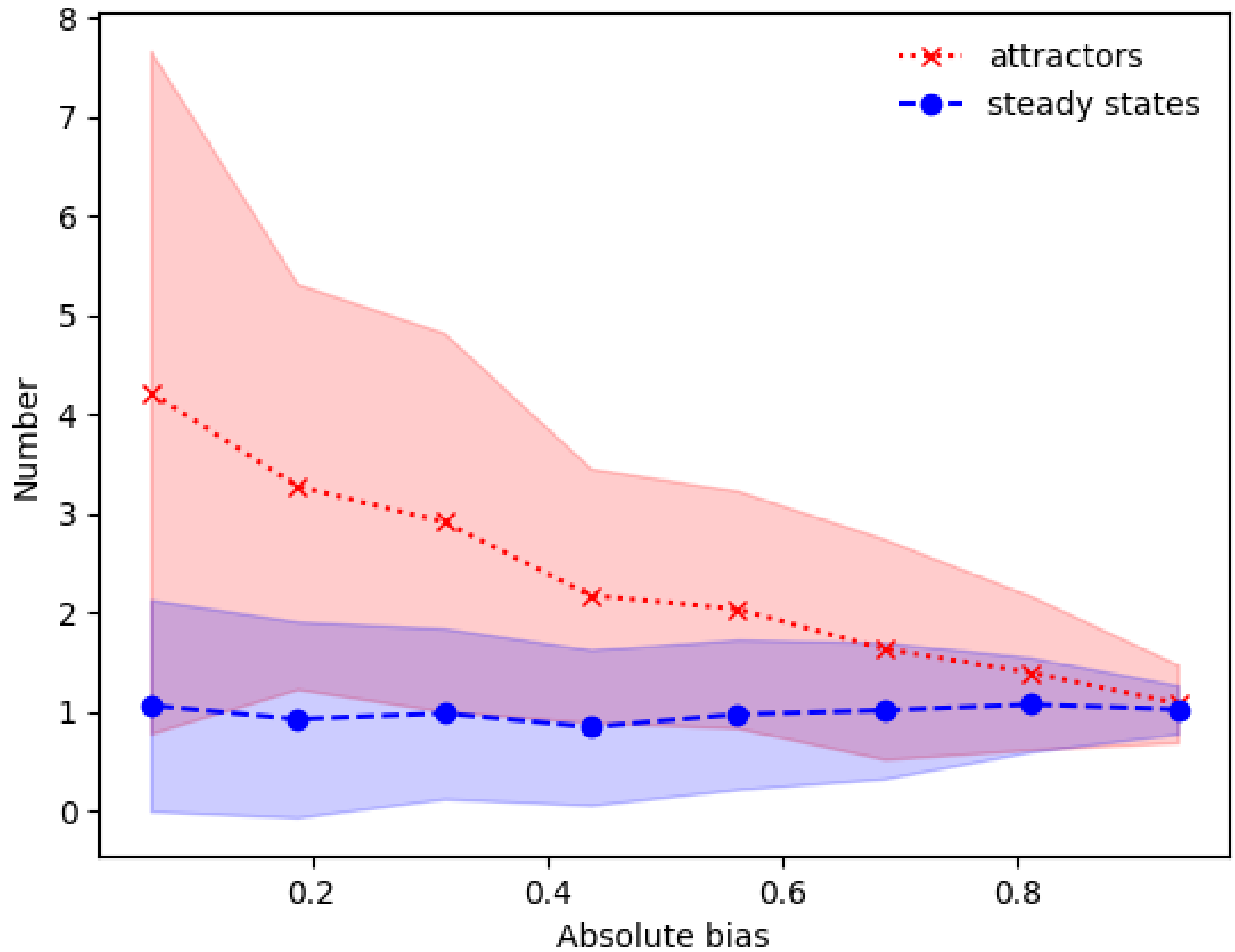

This plot shows: The higher the absolute bias of the governing nested canalizing functions, the more ordered the dynamics, characterized by the presence of only a few network attractors, which are primarily steady states.

10.6 Summary and outlook

In this tutorial you learned how to:

- compute exact robustness measures for small Boolean networks,
- interpret coherence and fragility at network, basin, and attractor levels,
- approximate robustness measures for larger networks, and
- assess dynamical sensitivity using the Derrida value.

In Tutorial 11, we will finally analyze biological Boolean network models and design null models and ensemble experiments.

11 Curated biological Boolean networks and null models

In this tutorial, we study how to analyze curated biological Boolean networks.

11.1 What you will learn

You will learn how to:

- load repositories of curated biological Boolean network models,
- analyze these models,
- generate null models to test the statistical significance of features in biological models.

These tools enable real research findings, namely the identification of design principles of regulatory functions and networks.

11.2 Setup

```
import boolforge as bf
import numpy as np
import matplotlib.pyplot as plt
from scipy.stats import ttest_rel
```

11.3 Loading model repositories

BoolForge makes it very easy to load all models included in three different repositories of curated biological Boolean networks.

```
models = bf.get_bio_models_from_repository(simplify_functions=True)
bns = models['BooleanNetworks']
n_models = len(bns)
```

The function `get_bio_models_from_repository` loads, by default, all 122 distinct biological Boolean network models, analyzed in Kadelka et al. [2024], and deposited in a Github repository. The models are parsed directly from the associated Github repository, meaning a wireless connection is required to successfully execute this function. Setting the optional parameter `simplify_functions=True` ensures that all update functions are non-degenerate. This is important for correct null model computation. By default, because this procedure may be very time-confusing for networks with very high degree, `simplify_functions=False`. Note that any `BooleanNetwork` object `bn` can be simplified at any time, using `bn.simplify_functions()`.

Models from the two other available repositories can be loaded by selecting the respective Github repository name:

- 'pystablemotifs (jcrozum)'
- 'biodivine (sybila)'

```
models_sm = bf.get_bio_models_from_repository('pystablemotifs (jcrozum)',
                                              simplify_functions=True)
bns_sm = models_sm['BooleanNetworks']
n_models_sm = len(bns_sm)

#models_bd = bf.get_bio_models_from_repository('biodivine (sybila)',

#                                               simplify_functions=True)
#n_models_bd = len(models_bd)
#bns_bd = models_bd['BooleanNetworks']
```

Note that the last repository is very large, which is why this code is commented out.

11.4 Analyzing model repositories

By applying BoolForge functions to all models in a repository, we can swiftly generate summary statistics, such as the size distribution of the models, or their average degree.

```
sizes = [bn.N for bn in bns]
average_degrees = [np.mean(bn.indegrees) for bn in bns]
```

Plotting the size of a model against its average essential degree (essential because we removed all non-essential inputs by setting `simplify_functions=True`), we observe that, for these models, there exists no strong correlation between size and degree.

```
sizes_sm = [bn.N for bn in bns_sm]
average_degrees_sm = [np.mean(bn.indegrees) for bn in bns_sm]

f,ax = plt.subplots()
ax.semilogx(sizes, average_degrees, 'rx',
            label = 'expert-curated (ckadelka)')
ax.semilogx(sizes_sm, average_degrees_sm, 'bo',
            label = 'pystablemotifs (jcrozum)')
ax.set_xlabel('network size')
ax.set_ylabel('average essential degree')
ax.legend(loc='best',frameon=False);
```

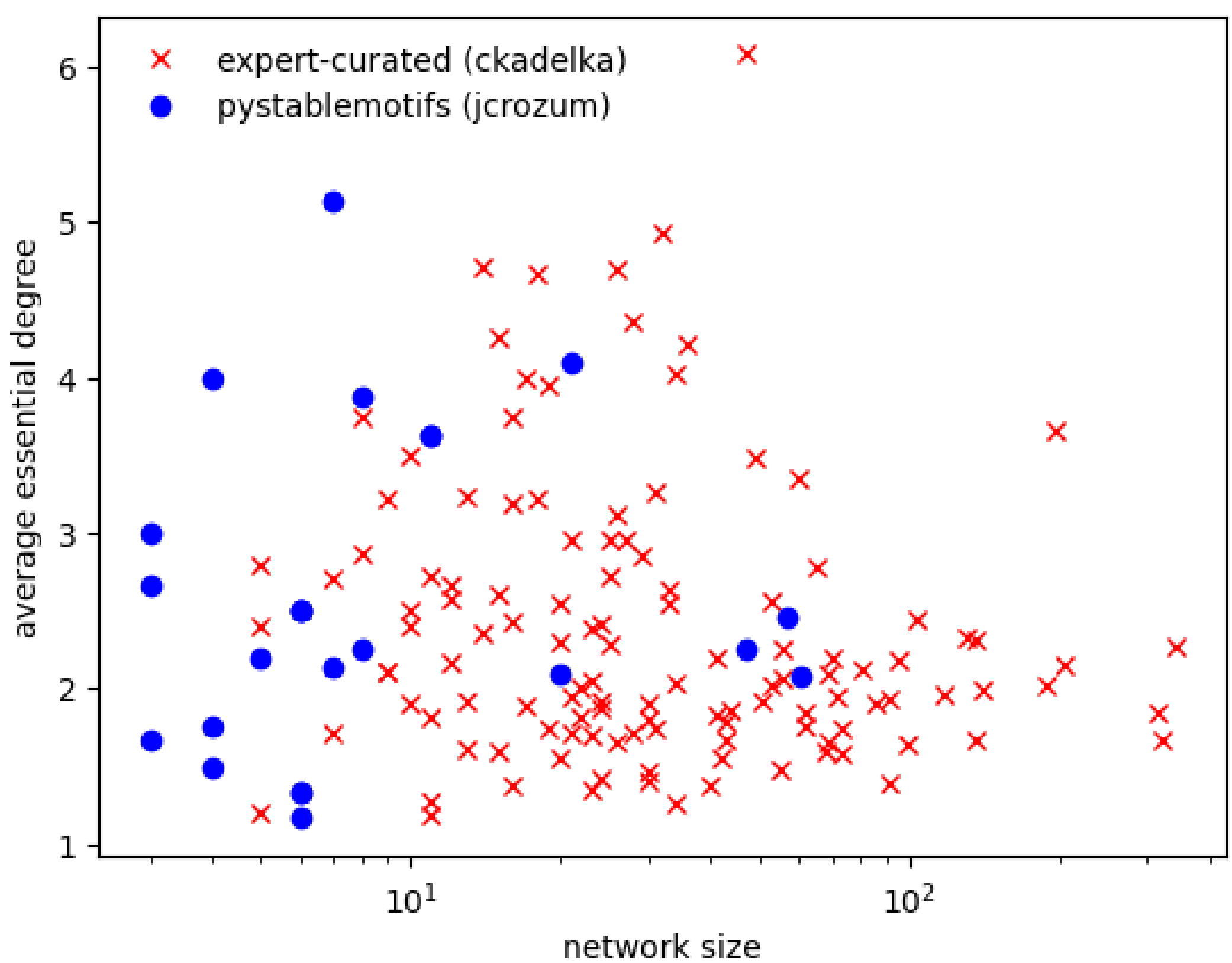

11.5 Null models

Observed properties of Boolean networks are often difficult to interpret in isolation. For example, a network may exhibit a certain number of attractors, a particular robustness to perturbations, or a specific Derrida

value. However, it is not immediately clear whether such properties are meaningful or simply typical for networks with the same size and structural characteristics.

To address this question, researchers compare observed networks with *null models*: randomly generated Boolean networks that preserve selected structural features, such as the number of nodes, the wiring diagram, or the bias of regulatory functions. By analyzing ensembles of such randomized networks, it becomes possible to determine whether the behavior of a given network is unusual or expected (see e.g., Kadelka and Murrugarra [2024]).

The BoolForge function `random_null_model(BooleanNetwork, *args)` provides extensive tools for generating these null models and for performing ensemble-based analyses that connect structural properties of Boolean functions and networks with their dynamical behavior.

The function takes as required input a Boolean network. Important: This network may not contain any degenerate update functions. If it does, these functions must be simplified via `bn.simplify_functions()` prior to generating null models. The avoid repeating this step many times, this simplification is not performed inside `random_null_model`. The type of null model is specified by optional arguments. Both the wiring diagram and the Boolean update rules can be randomized subject to specified invariants.

11.5.1 Randomization of the wiring diagram

By default, the wiring diagram of the provided Boolean network is not changed. However, setting `wiring_diagram="fixed_indegree"` generates a new wiring diagram using `random_wiring_diagram`. Each node in the new wiring diagram has exactly the same in-degree as in the provided Boolean network.

```
bn_orig = bf.random_network(N=8, n=2, indegree_distribution='Poisson', rng = 3)

bn_null = bf.random_null_model(bn_orig,
                               wiring_diagram='fixed_indegree')

print('bn_orig.in-degrees:',bn_orig.indegrees)
print('bn_null.in-degrees:',bn_null.indegrees)
print()
print('bn_orig.out-degrees:',bn_orig.outdegrees)
print('bn_null.out-degrees:',bn_null.outdegrees)
```

```
bn_orig.in-degrees: [1 2 1 2 1 2 3 2]
bn_null.in-degrees: [1 2 1 2 1 2 3 2]

bn_orig.out-degrees: [4 3 1 0 1 0 1 4]
bn_null.out-degrees: [2 2 3 1 0 1 2 3]
```

We see that the in-degrees of the original Boolean network are preserved, while the out-degrees change substantially. Additional optional arguments in the `fixed_indegree` mode include `strongly_connected`, `allow_self_loops`, and `min_out_degree_one`, as described in detail in Tutorial 9.

A more constrained null model fixes the out-degree in addition to the in-degree. This can be obtained by setting `wiring_diagram="fixed_in_and_outdegree"`.

```
bn_orig = bf.random_network(N=8, n=2, indegree_distribution='Poisson', rng = 3)

bn_null = bf.random_null_model(bn_orig,
                               wiring_diagram='fixed_in_and_outdegree')

print('bn_orig.in-degrees:',bn_orig.indegrees)
print('bn_null.in-degrees:',bn_null.indegrees)
print()
print('bn_orig.out-degrees:',bn_orig.outdegrees)
print('bn_null.out-degrees:',bn_null.outdegrees)
```

```
bn_orig.in-degrees: [1 2 1 2 1 2 3 2]
bn_null.in-degrees: [1 2 1 2 1 2 3 2]

bn_orig.out-degrees: [4 3 1 0 1 0 1 4]
bn_null.out-degrees: [4 3 1 0 1 0 1 4]
```

In the `fixed_in_and_outdegree` mode, the original wiring diagram is rewired through an edge-swapping algorithm. Additional optional arguments that can be used in this mode include `allow_new_self_loops` and `allow_self_loop_rewiring`.

11.5.2 Randomization of the update functions

In addition to the wiring diagram, the Boolean update functions can also be randomized. This behavior is controlled by two Boolean flags:

- `preserve_bias`: If True (default), the newly generated update function of each node has the same Hamming weight (number of ones in the truth table) as the original update function.
- `preserve_canalizing_depth`: If True (default), the newly generated update function of each node has the same canalizing depth as the original update function.

If both flags are True (the default), both properties are preserved simultaneously. If neither flag is True, the newly generated update rules may be any non-degenerate Boolean function consistent with the given in-degree.

```python
# 8-node network governed by 3-input functions with minimum canalizing depth 1
bn_orig = bf.random_network(N=8, n=3, depth=1, rng = 6)

bn_null00 = bf.random_null_model(bn_orig,
                                preserve_bias=False,
                                preserve_canalizing_depth=False)
bn_null01 = bf.random_null_model(bn_orig,
                                preserve_bias=False,
                                preserve_canalizing_depth=True)
bn_null10 = bf.random_null_model(bn_orig,
                                preserve_bias=True,
                                preserve_canalizing_depth=False)
bn_null11 = bf.random_null_model(bn_orig,
                                preserve_bias=True,
                                preserve_canalizing_depth=True)

print('Canalizing depths:')
print('bn_orig:  ',
      [f.get_canalizing_depth() for f in bn_orig.F])
print('bn_null00:',
      [f.get_canalizing_depth() for f in bn_null00.F])
print('bn_null01:',
      [f.get_canalizing_depth() for f in bn_null01.F])
print('bn_null10:',
      [f.get_canalizing_depth() for f in bn_null10.F])
print('bn_null11:',
      [f.get_canalizing_depth() for f in bn_null11.F])
print()
print('Hamming weights:')

print('bn_orig:  ',
      [f.hamming_weight for f in bn_orig.F])
print('bn_null00:',
```

```
      [f.hamming_weight for f in bn_null00.F])
print('bn_null01:',
      [f.hamming_weight for f in bn_null01.F])
print('bn_null10:',
      [f.hamming_weight for f in bn_null10.F])
print('bn_null11:',
      [f.hamming_weight for f in bn_null11.F])
```

```
Canalizing depths:
bn_orig:   [3, 3, 1, 1, 3, 3, 1, 3]
bn_null00: [0, 0, 1, 0, 0, 1, 0, 3]
bn_null01: [3, 3, 1, 1, 3, 3, 1, 3]
bn_null10: [0, 3, 1, 1, 3, 0, 1, 3]
bn_null11: [3, 3, 1, 1, 3, 3, 1, 3]

Hamming weights:
bn_orig:   [3, 5, 6, 6, 1, 5, 2, 7]
bn_null00: [4, 4, 6, 2, 4, 6, 4, 3]
bn_null01: [3, 5, 2, 6, 7, 5, 2, 3]
bn_null10: [3, 5, 6, 6, 1, 5, 2, 7]
bn_null11: [3, 5, 6, 6, 1, 5, 2, 7]
```

We see that the preserved structural constraints determine which properties of the original network are retained in the null models. Such controlled randomization allows systematic investigation of how structural features influence network dynamics.

11.6 Example use case: high coherence of biological networks

As an example, we compare the *coherence* of curated biological Boolean network models with the coherence expected under randomized null models (see Bavisetty et al. [2026]). Coherence measures the long-term resilience of a network to small perturbations.

For each biological network, we generate an ensemble of randomized null models in which the wiring diagram is preserved but the Boolean update rules are replaced by random Boolean functions. We then compare the coherence of the biological model with the average coherence of its corresponding null models.

```
n_null_models = 50

bns_to_analyze = [bn for bn in bns if bn.N <= 16]
bio_data = [bn.get_attractors_and_robustness_synchronous_exact()['Coherence']
            for bn in bns_to_analyze]

null_data = []
for bn in bns_to_analyze:
    null_data.append([])
    for _ in range(n_null_models):
        null_model = bf.random_null_model(bn,
                                   preserve_bias=False,
                                   preserve_canalizing_depth=False)
        null_data[-1].append(
            null_model.get_attractors_and_robustness_synchronous_exact()['Coherence']
        )

f,ax = plt.subplots()
ax.plot(bio_data,np.mean(null_data,1),'x')
ax.plot([0,1],[0,1],'r--')
ax.set_aspect('equal', adjustable='box')
ax.set_xlabel('Biological network coherence')
```

```
ax.set_ylabel('Average null model coherence')
stat, p = ttest_rel(bio_data,np.mean(null_data,1), alternative='greater')
p_str = f"{p:.2g}" if p >= 1e-3 else "< 0.001"
ax.set_title(f"One-sided paired t-test: p {('= ' if p >= 1e-3 else '')}{p_str}");
```

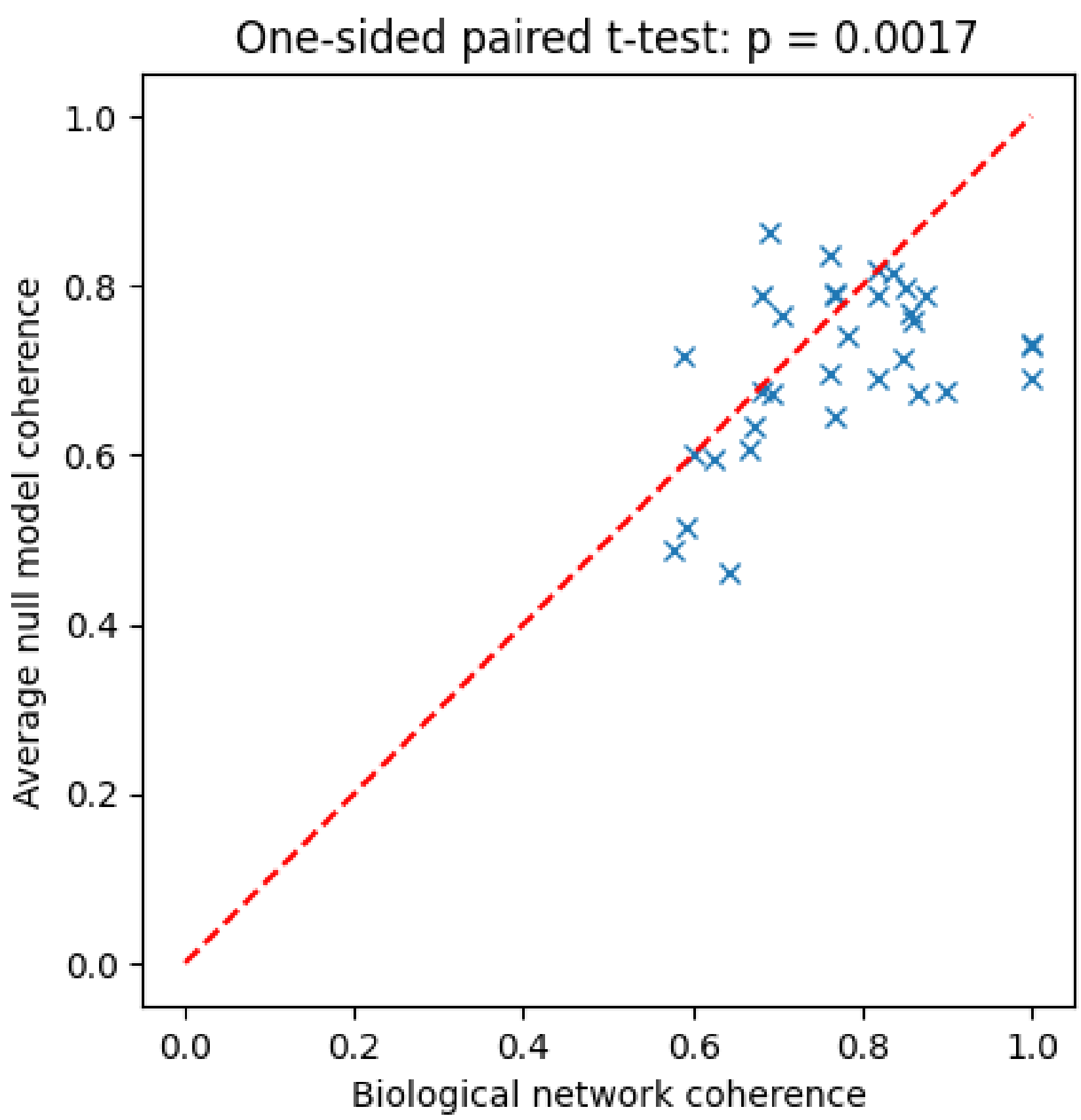

We see that most biological networks exhibit higher than expected coherence. Even for this small ensemble of biological networks (restricted here to networks with at most 16 nodes to allow exact dynamical analysis), this is a statistically significant difference, as exemplified by the one-sided paired t-test.

The higher coherence observed in biological networks is likely due to their highly biased and canalized regulatory logic (see Bavisetty et al. [2026]). To test this in BoolForge, we can rerun the computational experiment, this time with null models where bias and/or canalizing depth are preserved.

```
null_data = []
for i,bn in enumerate(bns_to_analyze):
    null_data.append([])
    for _ in range(n_null_models):
        null_model = bf.random_null_model(bn,
                                          preserve_bias=True,
                                          preserve_canalizing_depth=True)
        null_data[-1].append(
            null_model.get_attractors_and_robustness_synchronous_exact()['Coherence']
        )

f,ax = plt.subplots()
ax.plot(bio_data,np.mean(null_data,1),'x')
ax.plot([0,1],[0,1],'r--')
ax.set_aspect('equal', adjustable='box')
```

```
ax.set_xlabel('Biological network coherence')
ax.set_ylabel('Average null model coherence');
stat, p = ttest_rel(bio_data,np.mean(null_data,1), alternative='greater')
p_str = f"{p:.2g}" if p >= 1e-3 else "< 0.001"
ax.set_title(f"One-sided paired t-test: p {('= ' if p >= 1e-3 else '')}{p_str}");
```

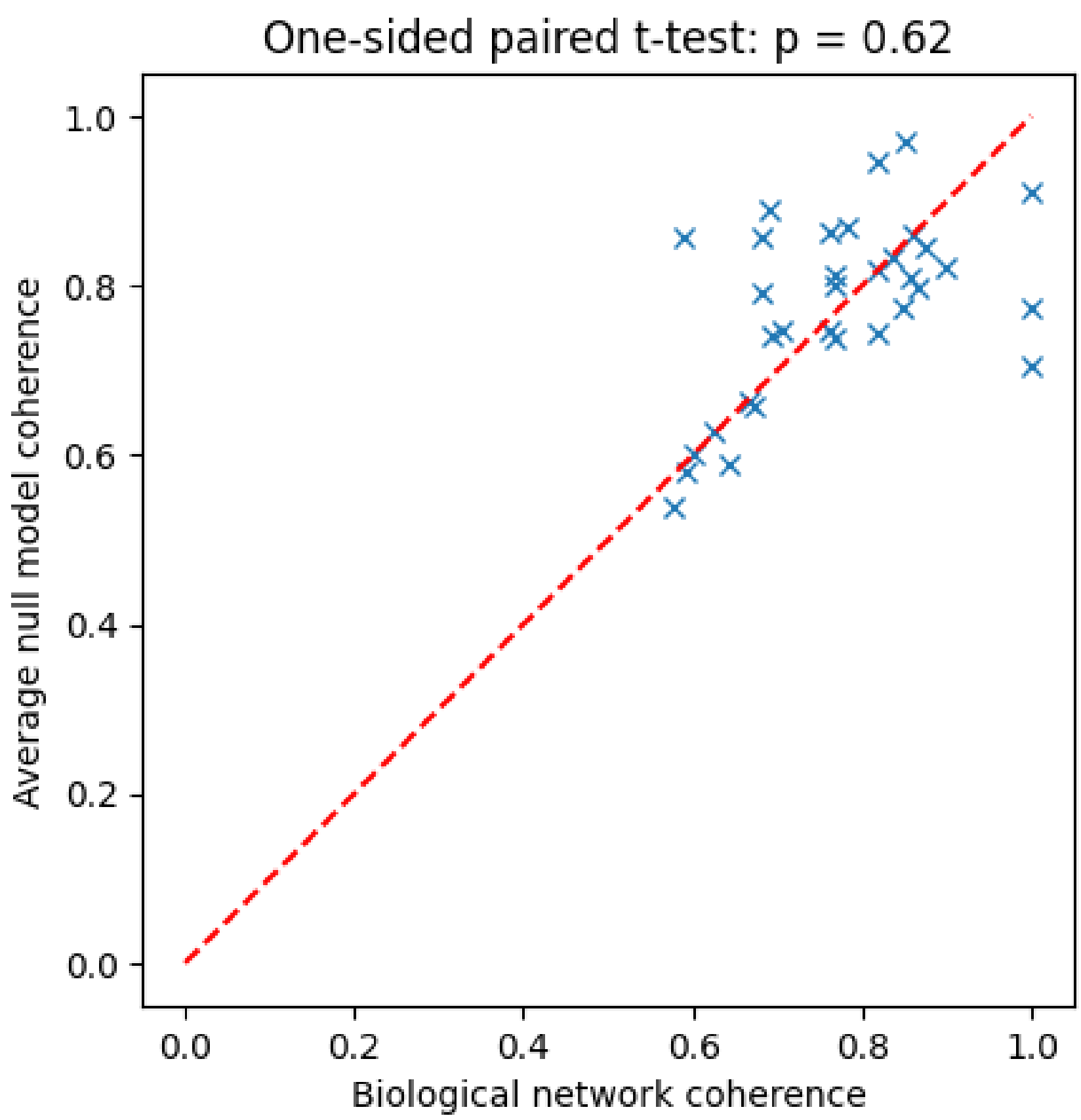

We observe that matching canalizing depth and bias (or just one of them, try it!) suffices to eliminate the significant difference in coherence between biological networks and their null models.

This illustrates how controlled null models can reveal which structural properties of biological regulatory logic are responsible for observed dynamical behavior.

11.7 Summary

In this tutorial, we introduced *null models for Boolean networks* and demonstrated how BoolForge can generate randomized networks while preserving selected structural properties. Such null models provide a statistical baseline that helps determine whether observed structural or dynamical properties of a Boolean network are unusual or simply typical for networks with similar characteristics.

We considered two main classes of null models:

- *Wiring diagram randomization*, where the regulatory graph is modified while preserving invariants such as node in-degrees or both in- and out-degrees.
- *Update function randomization*, where Boolean update rules are replaced by new functions that preserve properties such as bias or canalizing depth.

In addition, we demonstrated how Boolean network models can be loaded from biological model repositories and analyzed using the same structural and dynamical tools provided by BoolForge. This enables systematic

investigation of curated regulatory network models and comparison with appropriate null models.

Together, these capabilities allow researchers to study how structural features of regulatory networks influence their dynamical behavior and to place biological models in the broader context of ensembles of randomized networks.

References

Venkata Sai Narayana Bavisetty, Matthew Wheeler, Reinhard Laubenbacher, and Claus Kadelka. Upper bound for the stability of Boolean networks. *Physical Review E*, 112(4):044310, 2025.

Venkata Sai Narayana Bavisetty, Matthew Wheeler, and Claus Kadelka. Attractors are less stable than their basins: Canalization creates a coherence gap in gene regulatory networks. *PRX Life*, 4(2):023019, 2026.

Enrico Borriello and Bryan C Daniels. The basis of easy controllability in Boolean networks. *Nature communications*, 12(1):5227, 2021.

Karthik Chandrasekhar, Claus Kadelka, Reinhard Laubenbacher, and David Murrugarra. Stability of linear Boolean networks. *Physica D: Nonlinear Phenomena*, 451:133775, 2023.

Bryan C Daniels, Hyunju Kim, Douglas Moore, Siyu Zhou, Harrison B Smith, Bradley Karas, Stuart A Kauffman, and Sara I Walker. Criticality distinguishes the ensemble of biological regulatory networks. *Physical review letters*, 121(13):138102, 2018.

Bernard Derrida and Yves Pomeau. Random networks of automata: a simple annealed approximation. *Europhysics letters*, 1(2):45, 1986.

Elena Dimitrova, Brandilyn Stigler, Claus Kadelka, and David Murrugarra. Revealing the canalizing structure of Boolean functions: Algorithms and applications. *Automatica*, 146:110630, 2022.

Michael B Elowitz and Stanislas Leibler. A synthetic oscillatory network of transcriptional regulators. *Nature*, 403(6767):335–338, 2000.

Alexander J Gates, Rion Brattig Correia, Xuan Wang, and Luis M Rocha. The effective graph reveals redundancy, canalization, and control pathways in biochemical regulation and signaling. *Proceedings of the National Academy of Sciences*, 118(12):e2022598118, 2021.

Qijun He and Matthew Macauley. Stratification and enumeration of Boolean functions by canalizing depth. *Physica D: Nonlinear Phenomena*, 314:1–8, 2016.

Claus Kadelka. Canalization as a stabilizing principle of gene regulatory networks: a discrete dynamical systems perspective. *npj Systems Biology and Applications*, 2026.

Claus Kadelka and Benjamin Coberly. BoolForge: Random generation and analysis of Boolean functions and networks in Python. *arXiv preprint arXiv:2509.02496*, 2025.

Claus Kadelka and David Murrugarra. Canalization reduces the nonlinearity of regulation in biological networks. *npj Systems Biology and Applications*, 10(1):67, 2024.

Claus Kadelka, Jack Kuipers, and Reinhard Laubenbacher. The influence of canalization on the robustness of Boolean networks. *Physica D: Nonlinear Phenomena*, 353:39–47, 2017.

Claus Kadelka, Benjamin Keilty, and Reinhard Laubenbacher. Collectively canalizing Boolean functions. *Advances in Applied Mathematics*, 145:102475, 2023.

Claus Kadelka, Taras-Michael Butrie, Evan Hilton, Jack Kinseth, Addison Schmidt, and Haris Serdarevic. A meta-analysis of Boolean network models reveals design principles of gene regulatory networks. *Science Advances*, 10(2):eadj0822, 2024.

Stuart Kauffman. Metabolic stability and epigenesis in randomly constructed genetic nets. *Journal of Theoretical Biology*, 22(3):437–467, 1969.

Stuart A Kauffman. The large scale structure and dynamics of gene control circuits: an ensemble approach. *Journal of Theoretical Biology*, 44(1):167–190, 1974.

Lori Layne, Elena Dimitrova, and Matthew Macauley. Nested canalyzing depth and network stability. *Bulletin of mathematical biology*, 74(2):422–433, 2012.

Shmoolik Mangan and Uri Alon. Structure and function of the feed-forward loop network motif. *Proceedings of the National Academy of Sciences*, 100(21):11980–11985, 2003.

Austin M Marcus, Jordan Rozum, Herbert Sizek, and Luis M Rocha. CANA v1.0.0: efficient quantification of canalization in automata networks. *Bioinformatics*, page btaf461, 08 2025.

David Murrugarra, Alan Veliz-Cuba, Boris Aguilar, and Reinhard Laubenbacher. Identification of control targets in Boolean molecular network models via computational algebra. *BMC systems biology*, 10(1):94, 2016.

Kyu Hyong Park, Felipe Xavier Costa, Luis M Rocha, Réka Albert, and Jordan C Rozum. Models of cell processes are far from the edge of chaos. *PRX life*, 1(2):023009, 2023.

CJ Olson Reichhardt and Kevin E Bassler. Canalization and symmetry in boolean models for genetic regulatory networks. *Journal of Physics A: Mathematical and Theoretical*, 40(16):4339–4350, 2007.

Jordan C Rozum, Dávid Deritei, Kyu Hyong Park, Jorge Gómez Tejeda Zañudo, and Réka Albert. pystablemotifs: Python library for attractor identification and control in Boolean networks. *Bioinformatics*, 38(5): 1465–1466, 2022.

Ilya Shmulevich and Stuart A Kauffman. Activities and sensitivities in Boolean network models. *Physical review letters*, 93(4):048701, 2004.

Conrad H Waddington. Canalization of development and the inheritance of acquired characters. *Nature*, 150 (3811):563–565, 1942.

Kai Willadsen, Jochen Triesch, and Janet Wiles. Understanding robustness in random Boolean networks. In *Proceedings of the Eleventh International Conference on the Simulation and Synthesis of Living Systems (ALife XI)*, Winchester, U.K., August 2008. MIT Press.
